# Soil and leaf litter ants from the Amazon Region offer new distribution records for Colombia

**DOI:** 10.3897/BDJ.13.e142813

**Published:** 2025-05-05

**Authors:** Brandon S. Arredondo, Yennifer Carreño-Guevara, Yenifer Gutierrez-Villanueva, Ervin Humprey Duran-Bautista, Jean Gamboa-Tabares, Roberto J. Guerrero

**Affiliations:** 1 Laboratorio de Ecología del Suelo, Universidad de la Amazonia, Florencia, Caquetá, Colombia Laboratorio de Ecología del Suelo, Universidad de la Amazonia Florencia, Caquetá Colombia; 2 Laboratorio de Entomología Universidad de la Amazonia (LEUA), Universidad de la Amazonia, Florencia, Caquetá, Colombia Laboratorio de Entomología Universidad de la Amazonia (LEUA), Universidad de la Amazonia Florencia, Caquetá Colombia; 3 Universidad del Magdalena, Santa Marta, Colombia Universidad del Magdalena Santa Marta Colombia

**Keywords:** Caquetá Department, distributional range, diversity, formicidae, morphology

## Abstract

**Background:**

The Colombian Amazon is a region of remarkable biodiversity; however, several biological groups and their microhabitats remain poorly explored. Recent studies on soil ant diversity have provided new records and insights into their biology. Sampling techniques focused on exploring the soil interior (monoliths) and leaf litter have led to numerous new ant records for Colombia, as well as range expansions for many species previously known from other regions.

**New information:**

Seven new species records are reported for the country and the distribution of 14 species is extended within the Department of Caquetá. These 21 records belong to five subfamilies and sixteen genera. For the first time, the genera *Lenomyrmex* (Fernández & Palacio), *Myrmelachista* (Roger), *Oxyepoecus* (Santschi) and *Stegomyrmex* (Emery) are recorded for the Colombian Amazon. Additionally, the first case of an ergatoid queen in *Probolomyrmexkelleri* (Oliveira & Feitosa) is documented. The northernmost records of *Adelomyrmexstriatus* (Fernández) and *Centromyrmexgigas* (Forel) are also reported. Morphological observations, distribution data and images of all recorded species are included. The specimens were collected using the Tropical Soil Biology and Fertility (TSBF) and Winkler extraction methodologies. Furthermore, we present a checklist of ants from Caquetá, listing **321** species for the Department. Finally, we update the known ant diversity of Colombia, reporting a total of **1.280** species and **110** genera.

## Introduction

Colombia is a megadiverse country (Rodríguez-Zapata and Ruíz-Agudelo 2021). In fact, the number of species of birds, orchids and butterflies in Colombia is higher than in any other country in the world (Arbelaez-Cortes 2013). This high biodiversity is concentrated in three areas or hotspots: the Amazon, the Andes and the Chocó-Darién. These areas are home to a large number of endemic species, but are threatened by various human activities (e.g. mining, illicit crops, armed conflict), which have led to habitat degradation and transformation (Dávalos et al. 2016). These hotspots have received considerable attention in research on plants, vertebrates and certain charismatic insect groups, such as butterflies. However, the situation is different for ants, which have been studied systematically, but on a relatively smaller geographic scale (e.g. the Sierra Nevada de Santa Marta by Guerrero and Sarmiento (2010)). Different studies suggest that forests in the Colombian Amazon, the Andes and the Chocó-Darién show high species diversity and elevated levels of ant endemism (Guénard et al. 2012, Kass et al. 2022). Kass et al. (2022) estimated that various areas of Colombia should be systematically explored to better understand ant diversity and its potential use in conservation and biogeography studies. In this context, specific ecosystems such as the tropical dry forests found in the inter-Andean valleys, the eastern and Caribbean regions of Colombia (Pizano et al. 2014), as well as the Chocó-Darién moist forests, the Andean foothills, the Amazon rainforest and the transition zones connecting the Amazon to the Andes (i.e. the Andean-Amazonian transition, AATZ) should be prioritised in ant studies due to their potential to yield new records and species for Colombia.

The intermediate or transition zones, with variations in altitude, precipitation and topography, provide ideal conditions for harbouring many species (Ghazoul and Sheil 2010). In the Neotropics, this diversity of microclimates and microhabitats fosters the co-existence of a high diversity of ant species (Longino and Colwell 1997), primarily those living in the soil, both above and below ground. In this context, subterranean ants may represent the “final frontier” in the study of ant biodiversity, as soil is home to at least one-quarter of all extant ant species (MacKay and Vinson 1989, Belshaw and Bolton 1994, Delabie and Fowler 1995, Yamaguchi and Hasegawa 1996, Fowler et al. 2000, Weissflog et al. 2000, Berghoff et al. 2003a, Berghoff et al. 2003b, Silva and Silvestre 2004, Ryder et al. 2007, Ryder et al. 2010a, Ryder et al. 2010b, Turbé et al. 2010). The soil ant community of the Colombian Andean-Amazonian transition has been poorly studied, although there are preliminary lists that include them (Castro et al. 2018), which suggest the Amazon transition zone is an area of unique species concentration (i.e. high species turnover). Despite the limited studies, the foothills of the Colombian Amazon have been identified as a refuge for the ground-dwelling ant community (Chacón de Ulloa and Abadía 2014). However, more data are needed to support this hypothesis and, thus, there is a need to significantly increase sampling efforts using combined methodologies to capture ants at different soil depths, including those that live in leaf litter as the Tropical Soil Biology and Fertility method works.

The Tropical Soil Biology and Fertility (TSBF) monolith method (Anderson and Ingram 1993) has enabled sampling across different soil strata, generating valuable information on ants with subterranean habits (Castro et al. 2018, Castro et al. 2023). Earlier studies in the Colombian Andean-Amazonian transition have yielded important records, such as the first record for northern South America of *Gracilidrispombero* Wild and Cuezzo, 2006 (Guerrero and Sanabria 2011), as well as four new records of ants for Colombia (Castro et al. 2018) and ecological information on army ants (Sanabria-Blandón and Achury 2011). These results suggest that the foothills of the Colombian Amazon could harbour a veritable treasure trove of ant species, many of which are still not fully documented (Fragoso et al. 2001, Hättenschwiler et al. 2005, Ryder et al. 2007). To contribute to the knowledge of soil and sub-soil ant diversity in an Andean-Amazonian transition zone (AATZ), we sampled ants from ground level (including leaf litter) to 30 cm below ground, using Winkler sacs and the TSBF monolith method. We present seven new ant records for Colombia and extend the distribution range of fourteen species previously known from other regions of the country. Additionally, we expand the distribution of three genera previously unknown for the Colombian Amazon region (e.g. *Stegomyrmex*). Information on the biology and distribution of some of the recorded species is provided. The results are compared with previous studies in both the transition zone and other areas of the Amazon rainforest.

## Materials and methods

**Figure 1.**
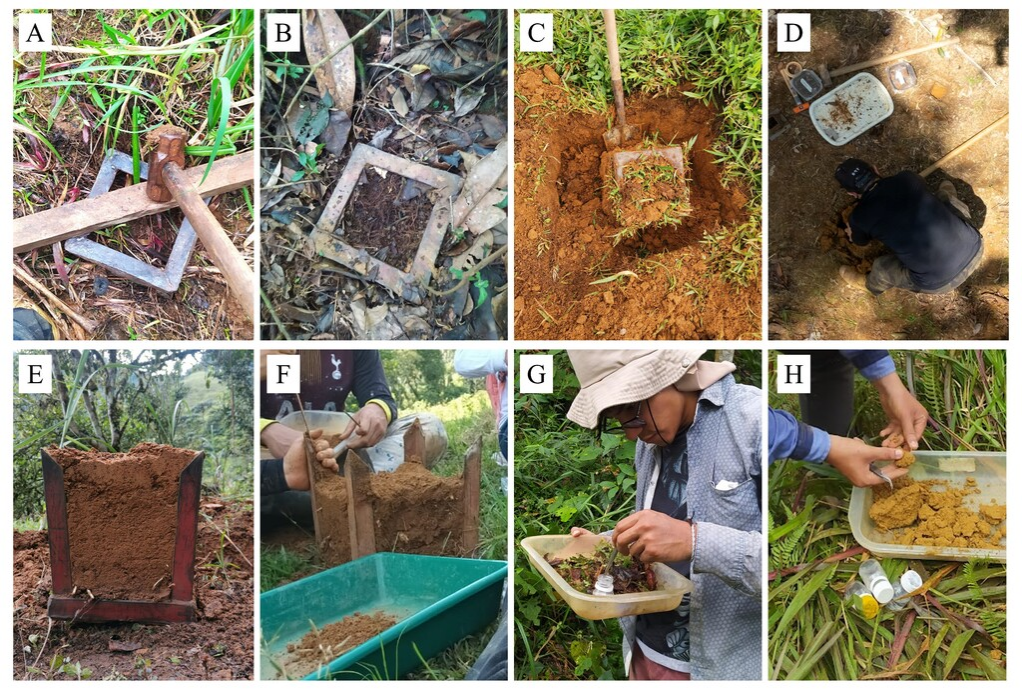
Methodology TSBF. **A** Introduction of the monolite into the soil by mechanical action; **B** Leaf litter removal for extraction with Winkler bags; **C** Organisation of the area for monolite extraction; **D** Extraction of the monolite; **E** Cleaning and organisation of the monolite **F** Stratification of the monolite where the deepest stratum (20-30 cm) was removed; **G** Manual revision of the superficial stratum (= A); **H** Manual revision of the stratum B.

Ants were collected using the TSBF methodology (Anderson and Ingram 1993, ISO 2024), using a 25 × 25 × 30 cm (length, width and depth, respectively) soil block (including the surface stratum that was composed of leaf litter), which was divided into four strata (Fig. 1): leaf litter stratum was separated and processed with Winkler sacs (Bestelmeyer et al. 2000) and the depths 0-10 cm (= A), 10-20 cm (= B) and 20-30 cm (= C) which were checked in the field, extracting the ants by manual means. All ants recorded here were deposited at the Laboratory of Entomology of the University of Amazonia (LEUA), Florencia, Caquetá, Colombia.

### Photomicrographs and SEM

High resolution images were generated with a Leica digital high-resolution camera (Type DFC450), attached to a LEICA M205 A automated stereomicroscope. Stacked images were processed with LAS montage module Leica®. SEM micrographs were made using the scanning electron microscope HITACHI Tabletop Microscope (TM4000Plus).

### Taxonomic identification

Taxonomic revisions were used for the identification of the specimens. Additionally, comparisons were made with high-resolution images (AntWeb 2024) and original descriptions of the species (Emery 1890, Fernández 2003a, Albuquerque and Brandão 2004). The following taxonomic keys were used: *Wheeler (1911)* for *Mycetomoellerius*, *Albuquerque and Brandão (2004)* for *Oxyepoecus*, *Feitosa et al. (2008)* for *Lachnomyrmex*, Feitosa et al. (2008) for *Stegomyrmex*, *Delsinne and Fernández (2011)* for *Lenomyrmex*, Lattke (2011) for *Leptogenys*, Ortíz and Fernández (2011) for *Dolichoderus*, Longino (2012) for *Adelomyrmex*, *Fernández et al. (2014)* for *Kempfidris*, Franco and Feitosa (2018) for *Centromyrmex*, *Fernández and Guerrero (2019)* for *Thaumatomyrmex*, *Oliveira and Feitosa (2019)* for *Probolomyrmex*, Pérez-Pedraza and Fernández (2019)for *Strumigenys*, Branstetter and Longino (2022) for *Wadeura*, and *Probst and Brandão (2022)* for *Basiceros*.

### Specimen information and distribution maps

The information associated with each specimen was transcribed directly from its labels. The distribution maps were generated with Quantum GIS v.3.2. (QGIS Development Team 2024).

## Taxon treatments

### Dolichoderus debilis

Emery, 1890

5EE1093E-F952-5C54-A4B4-E08CD2FD7E27

#### Materials

- **Type status:** Other material. **Occurrence:** catalogNumber: LEUA-00000061181; recordedBy: Kenna Martinez; individualCount: 1; sex: female; occurrenceID: 5756BD00-C458-5C94-86AD-E5527B198C60; **Taxon:** scientificName: Dolichoderusdebilis; kingdom: Animalia; phylum: Arthropoda; class: Insecta; order: Hymenoptera; family: Formicidae; genus: Dolichoderus; specificEpithet: debilis; scientificNameAuthorship: Emery, 1890; **Location:** continent: South America; country: Colombia; countryCode: CO; stateProvince: Caquetá; county: Florencia; locality: Vda. San Luis, Fca. El Carmen 1; verbatimElevation: 433 m; locationRemarks: Collected in leaf Litter; verbatimCoordinates: 01°40'34.2"N 75°37'39.2"W; verbatimCoordinateSystem: WGS84; **Event:** samplingProtocol: Monolite / TSBF; eventDate: 2022-12-20; **Record Level:** language: es; collectionID: RNC:270; institutionCode: Universidad de la Amazonia (UDLA); basisOfRecord: PreservedSpecimen
- **Type status:** Other material. **Occurrence:** catalogNumber: LEUA-00000061182; recordedBy: Kenna Martinez; individualCount: 1; sex: female; occurrenceID: 1F001B36-5B17-50C0-96B1-BA7398D2ABA5; **Taxon:** scientificName: Dolichoderusdebilis; kingdom: Animalia; phylum: Arthropoda; class: Insecta; order: Hymenoptera; family: Formicidae; genus: Dolichoderus; specificEpithet: debilis; scientificNameAuthorship: Emery, 1890; **Location:** continent: South America; country: Colombia; countryCode: CO; stateProvince: Caquetá; county: Florencia; locality: Vda. San Luis, Fca. El Carmen 1; verbatimElevation: 433 m; locationRemarks: Collected in leaf Litter; verbatimCoordinates: 01°40'34.2"N 75°37'39.2"W; verbatimCoordinateSystem: WGS84; **Event:** samplingProtocol: Monolite / TSBF; eventDate: 2022-12-20; **Record Level:** language: es; collectionID: RNC:270; institutionCode: Universidad de la Amazonia (UDLA); basisOfRecord: PreservedSpecimen

#### Diagnosis

**Figure 2.**
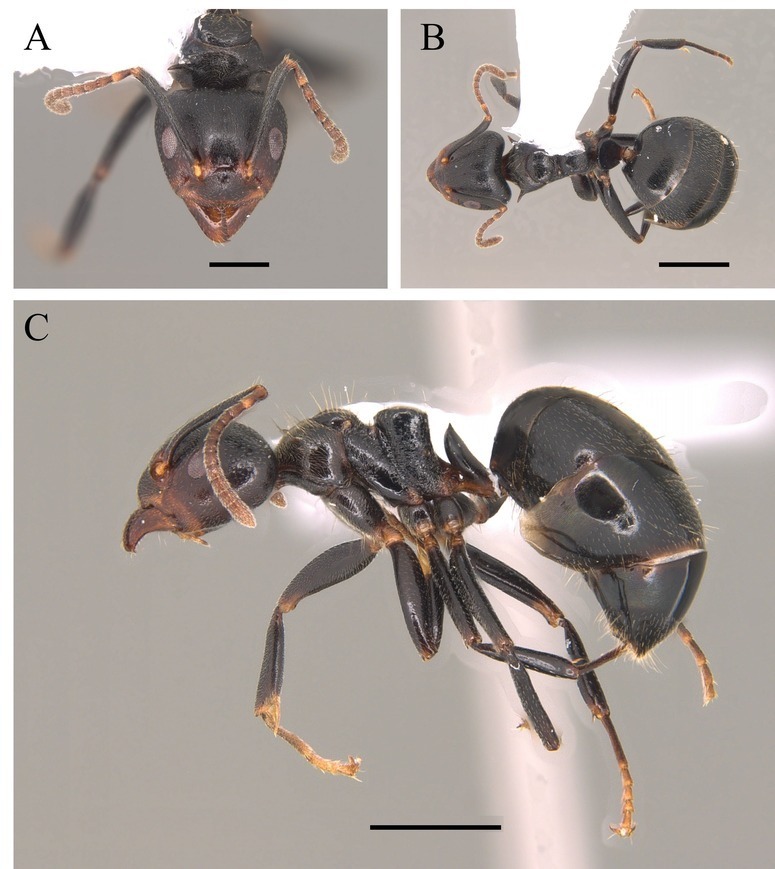
*Dolichoderusdebilis* worker (LEUA-00000061181) **A** head in frontal view; **B** body in dorsal view; **C** body in lateral view. Scale bars: 0.5 mm (A); 1.0 (B, C).

This species can be recognised by the absence of erect hairs on the scapes (small white erect hairs may be present), the presence of spines on the anterolateral angles of the pronotum (humeral angles) and the spine or tooth-shaped apical projection on the dorsal surface of the petiolar node (Fig. 2).

#### Distribution

*Dolichoderusdebilis* has a Neotropical distribution. In Colombia, it has been recorded in the Departments of Antioquia, Bolívar, Guajira, Magdalena and Santander (Fernández et al. 2019), as well as in the Departments of Amazonas, Meta and Putumayo in the Amazon Region (Ortíz and Fernández 2011). It is reported here for the first time in the Department of Caquetá. In the Neotropical Region, the species has also been recorded in Bolivia, Brazil, Peru, Guyana, Suriname, French Guiana, Panama, Costa Rica, Guatemala and Mexico (Kempf 1959, Fernandez and Sendoya 2004).

#### Biology

The specimens were collected in an Andean Amazonian transition zone (Amazonian foothills) in the cloud forest. It has been recorded from the rainforest (Mackay 1993). Some observations suggest that *D.debilis* is associated with the ant genus *Crematogaster* and with termites of the genus *Nasutitermes* (Forel 1898, Wheeler 1936).

### Dolichoderus rugosus

(Smith, 1858)

CBB767D9-7E0F-54FB-ACF6-DDC5CE3791AF

#### Materials

- **Type status:** Other material. **Occurrence:** catalogNumber: LEUA-00000061174; recordedBy: Brandon S. Arredondo; individualCount: 1; sex: female; occurrenceID: D3CDDED9-C277-59B2-9424-D334DFFC9313; **Taxon:** scientificName: Dolichoderusrugosus; kingdom: Animalia; phylum: Arthropoda; class: Insecta; order: Hymenoptera; family: Formicidae; genus: Dolichoderus; specificEpithet: rugosus; scientificNameAuthorship: (Smith, 1858); **Location:** continent: South America; country: Colombia; countryCode: CO; stateProvince: Caquetá; county: Florencia; locality: Vda. La Paz, Fca. El Mirador; verbatimElevation: 590 m; locationRemarks: Collected in leaf Litter; verbatimCoordinates: 01°43'48.1"N 75°38'33.3"W; verbatimCoordinateSystem: WGS84; **Event:** samplingProtocol: Monolite / TSBF; eventDate: 2023-02-22; **Record Level:** language: es; collectionID: RNC:270; institutionCode: Universidad de la Amazonia (UDLA); basisOfRecord: PreservedSpecimen
- **Type status:** Other material. **Occurrence:** catalogNumber: LEUA-00000061178; recordedBy: Brandon S. Arredondo; individualCount: 1; sex: female; occurrenceID: 1E152BB3-7CF8-5F4B-85BD-641F83A095A4; **Taxon:** scientificName: Dolichoderusrugosus; kingdom: Animalia; phylum: Arthropoda; class: Insecta; order: Hymenoptera; family: Formicidae; genus: Dolichoderus; specificEpithet: rugosus; scientificNameAuthorship: (Smith, 1858); **Location:** continent: South America; country: Colombia; countryCode: CO; stateProvince: Caquetá; county: Florencia; locality: Vda. La Paz, Fca. El Mirador; verbatimElevation: 590 m; locationRemarks: Collected in leaf Litter; verbatimCoordinates: 01°43'48.1"N 75°38'33.3"W; verbatimCoordinateSystem: WGS84; **Event:** samplingProtocol: Monolite / TSBF; eventDate: 2023-02-22; **Record Level:** language: es; collectionID: RNC:270; institutionCode: Universidad de la Amazonia (UDLA); basisOfRecord: PreservedSpecimen

#### Diagnosis

**Figure 3.**
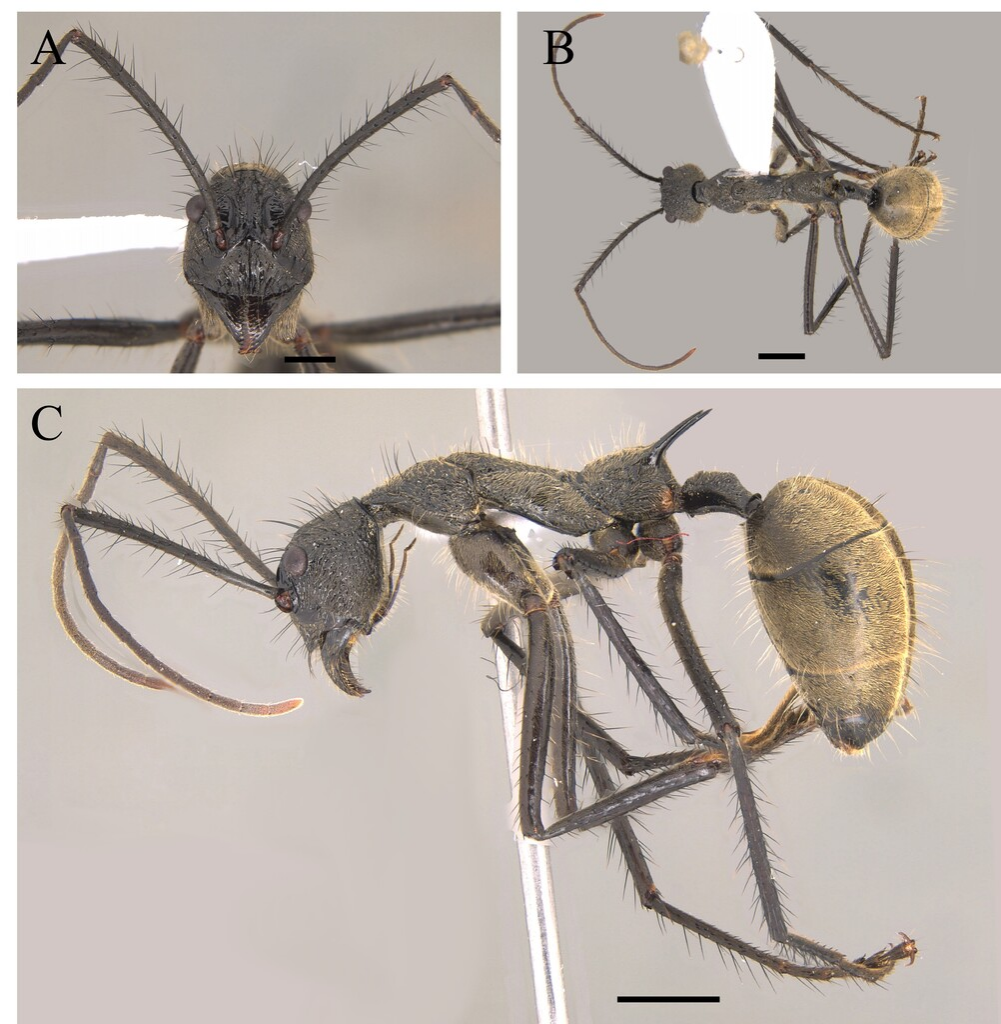
*Dolichoderusrugosus* worker (LEUA-00000062446) **A** head in frontal view; **B** body in dorsal view; **C** body in lateral view. Scale bars: 1.0 mm (A); 2.0 (B, C).

This species is one of the largest of the genus, recognised by the length of the maxillary palps which extend to the *foramen magnum*; furthermore, these ants have mesosoma extremely long and narrow, with the meso and metapleural longer than wide (Fig. 3).

#### Distribution

This species shows a Neotropical distribution with records only for South America, in the Amazon rainforest (Ortíz and Fernández 2011). It is reported here for the first time in the Department of Caquetá. In Colombia, it also has a record outside the Amazon Region, in the Department of Antioquia (Ortíz and Fernández 2011). In the Neotropical Region, the species has also been recorded in Bolivia, Brazil, Ecuador, Peru, Guyana, Suriname and French Guiana (Kempf 1969, Fernandez and Sendoya 2004).

#### Biology

Specimens record here were found in the leaf litter in the Rio Hacha Basin, being the predominant ant species in this highly conserved area (Andean-Amazonian transition zone in the Department of Caquetá).

### Myrmelachista schumanni

Emery, 1890

F8663D42-D6EB-5A4C-BAC9-27C8CB757D19

#### Materials

- **Type status:** Other material. **Occurrence:** catalogNumber: LEUA-00000066482; recordedBy: Brandon S. Arredondo; individualCount: 1; sex: female; occurrenceID: 5B1742BA-8699-5FE1-8D1B-472D1F07DD61; **Taxon:** scientificName: Myrmelachistaschumanni; kingdom: Animalia; phylum: Arthropoda; class: Insecta; order: Hymenoptera; family: Formicidae; genus: Myrmelachista; specificEpithet: schumanni; scientificNameAuthorship: Emery, 1890; **Location:** continent: South America; country: Colombia; countryCode: CO; stateProvince: Caquetá; county: Florencia; locality: Vía Florencia-Guadalupe; verbatimElevation: 923 m; locationRemarks: Collected in leaf Litter; verbatimCoordinates: 01°46'29.8"N 75°39'9.5"W; verbatimCoordinateSystem: WGS84; **Event:** samplingProtocol: Winkler; eventDate: 2023-08-12; **Record Level:** language: es; collectionID: RNC:270; institutionCode: Universidad de la Amazonia (UDLA); basisOfRecord: PreservedSpecimen

#### Diagnosis

**Figure 4.**
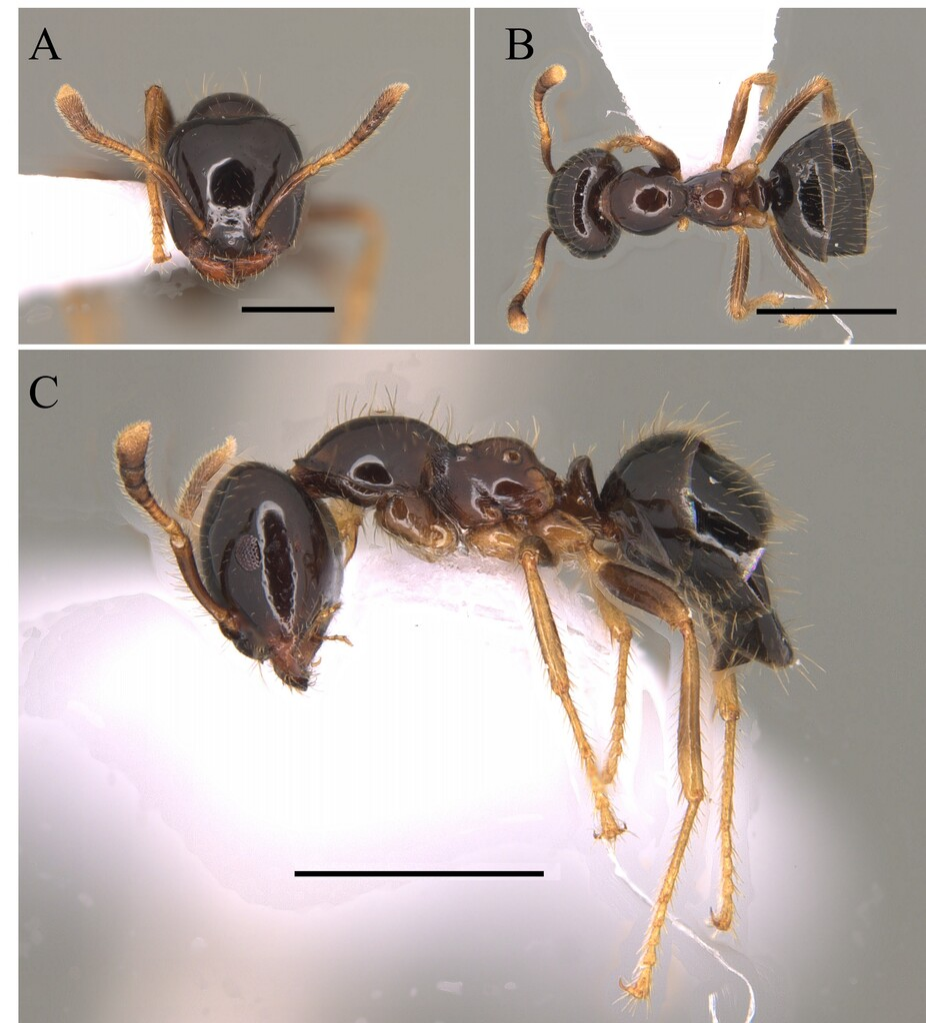
*Myrmelachistaschumanni* worker (LEUA-00000066482) **A** head in frontal view; **B** body in dorsal view; **C** body in lateral view. Scale bars: 0.5 mm (A); 1.0 (B, C).

The genus *Myrmelachista* is recognised by its five-toothed mandible, 9 or 10 segmented antennae, with a 3 or 4 segmented antennal club and a wedge-shaped, erect, prominent and exposed petiole (Fig. 4).

#### Distribution

Information on this species is scarce. In Colombia, it was known to be present, but without specific locality data (Fernández et al. 2019). It is reported here for the first time in the Department of Caquetá, in the Colombian Amazon Region. In the Neotropical Region, the species has also been recorded in Ecuador, Peru, Guyana and Venezuela (Kusnezov 1953, Frederickson 2005a, Salazar et al. 2015).

#### Biology

*M.schumanni* is recognised for its specific relationship with some plant species, forming the so-called Devil's gardens. This mutualistic interaction has been the subject of several studies (Frederickson et al. 2005, Frederickson 2005a, Frederickson 2005b, Frederickson and Gordon 2007), demonstrating the impact of this relationship in tropical rainforests. The collected specimen was sampled by leaf-litter sifting in a preserved area from a cloud forest. The presence of this species in leaf litter can be incidental due to the species' foraging behaviour on vegetation, which can result in its falling to the forest floor.

### Adelomyrmex striatus

Fernández, 2003

5973BDE1-1863-50AF-A856-CC2952E8D0F7

#### Materials

- **Type status:** Other material. **Occurrence:** catalogNumber: LEUA-00000066483; recordedBy: Fernando Celis; individualCount: 1; sex: female; occurrenceID: 6EE48905-53B7-560D-B507-09E3E930A680; **Taxon:** scientificName: Adelomyrmexstriatus; kingdom: Animalia; phylum: Arthropoda; class: Insecta; order: Hymenoptera; family: Formicidae; genus: Adelomyrmex; specificEpithet: striatus; scientificNameAuthorship: Fernández, 2003; **Location:** continent: South America; country: Colombia; countryCode: CO; stateProvince: Caquetá; county: Florencia; locality: Vda. Sucre, Fca. La Fortaleza; verbatimElevation: 1266 m; locationRemarks: Collected in leaf Litter; verbatimCoordinates: 01°48'22.1"N 75°39'12.7"W; verbatimCoordinateSystem: WGS84; **Event:** samplingProtocol: Winkler; eventDate: 2023-08-12; **Record Level:** language: es; collectionID: RNC:270; institutionCode: Universidad de la Amazonia (UDLA); basisOfRecord: PreservedSpecimen
- **Type status:** Other material. **Occurrence:** catalogNumber: LEUA-00000066484; recordedBy: Fernando Celis; individualCount: 1; sex: female; occurrenceID: 82AA0EC0-0A98-54BF-BA65-13AEBD3A82B4; **Taxon:** scientificName: Adelomyrmexstriatus; kingdom: Animalia; phylum: Arthropoda; class: Insecta; order: Hymenoptera; family: Formicidae; genus: Adelomyrmex; specificEpithet: striatus; scientificNameAuthorship: Fernández, 2003; **Location:** continent: South America; country: Colombia; countryCode: CO; stateProvince: Caquetá; county: Florencia; locality: Vda. Sucre, Fca. La Fortaleza; verbatimElevation: 1266 m; locationRemarks: Collected in leaf Litter; verbatimCoordinates: 01°48'22.1"N 75°39'12.7"W; verbatimCoordinateSystem: WGS84; **Event:** samplingProtocol: Winkler; eventDate: 2023-08-12; **Record Level:** language: es; collectionID: RNC:270; institutionCode: Universidad de la Amazonia (UDLA); basisOfRecord: PreservedSpecimen
- **Type status:** Other material. **Occurrence:** catalogNumber: LEUA-00000066485; recordedBy: Fernando Celis; individualCount: 1; sex: female; occurrenceID: B955E2A6-628A-5E83-B5B6-17CD517F32A4; **Taxon:** scientificName: Adelomyrmexstriatus; kingdom: Animalia; phylum: Arthropoda; class: Insecta; order: Hymenoptera; family: Formicidae; genus: Adelomyrmex; specificEpithet: striatus; scientificNameAuthorship: Fernández, 2003; **Location:** continent: South America; country: Colombia; countryCode: CO; stateProvince: Caquetá; county: Florencia; locality: Vda. Sucre, Fca. La Fortaleza; verbatimElevation: 1266 m; locationRemarks: Collected in leaf Litter; verbatimCoordinates: 01°48'22.1"N 75°39'12.7"W; verbatimCoordinateSystem: WGS84; **Event:** samplingProtocol: Winkler; eventDate: 2023-08-12; **Record Level:** language: es; collectionID: RNC:270; institutionCode: Universidad de la Amazonia (UDLA); basisOfRecord: PreservedSpecimen
- **Type status:** Other material. **Occurrence:** catalogNumber: LEUA-00000066486; recordedBy: Yenifer Gutierrez; individualCount: 1; sex: female; occurrenceID: 71FE94C8-DD46-58AF-B344-15BA8BD19B3E; **Taxon:** scientificName: Adelomyrmexstriatus; kingdom: Animalia; phylum: Arthropoda; class: Insecta; order: Hymenoptera; family: Formicidae; genus: Adelomyrmex; specificEpithet: striatus; scientificNameAuthorship: Fernández, 2003; **Location:** continent: South America; country: Colombia; countryCode: CO; stateProvince: Caquetá; county: Florencia; locality: Av.Caraño, Vía Florencia-Suaza; verbatimElevation: 1523 m; locationRemarks: Collected in leaf Litter; verbatimCoordinates: 01°43'10.7"N 75°43'10.5"W; verbatimCoordinateSystem: WGS84; **Event:** samplingProtocol: Winkler; eventDate: 2023-09-30; **Record Level:** language: es; collectionID: RNC:270; institutionCode: Universidad de la Amazonia (UDLA); basisOfRecord: PreservedSpecimen

#### Diagnosis

**Figure 5.**
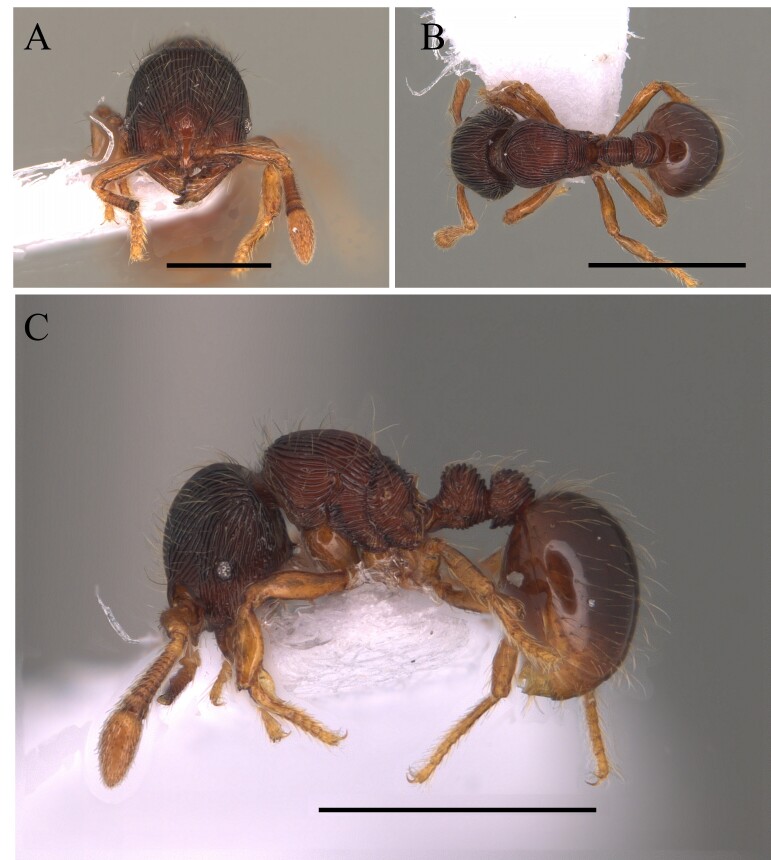
*Adelomyrmexstriatus* worker (LEUA-00000066483) **A** head in frontal view; **B** body in dorsal view; **C** body in lateral view. Scale bars: 0.5 mm (A); 1.0 mm (B, C).

This species is easily distinguished by sharp spiniform teeth on the median clypeal lobe and regular longitudinal striate sculpture on the head, pronotum, mesonotum, sides of mesosoma and petiole (Fig. 5).

#### Distribution

This record in the Andean-Amazonian transition zone of the Department of Caquetá represents the second record of the species in Colombia, suggesting a possible distributional restriction for the Amazon rainforest (Guerrero et al. 2018). In the Neotropical Region, the species has also been recorded in Ecuador, Peru and Brazil (Longino 2012).

#### Biology

Slow-moving ants difficult to distinguish from the substrate. All specimens were extracted from sifted leaf litter from the humid forest above 1200 m in the Andean-Amazonian transition zone studied.

#### Notes

The Colombian specimen presents yellowish, long and flexuous hairs on the body, but shorter than in the populations from Manaus (Amazonas, Brazil). A population from Peru presents variation in the teeth of the medial clypeal lobe, as well as finer sculpting in the frontal area of the head (Longino 2012).

### Basiceros conjugans

Brown, 1974

2D2DC3A7-21C4-5D08-A016-8989EDDC8F80

#### Materials

- **Type status:** Other material. **Occurrence:** catalogNumber: LEUA-00000061876; recordedBy: Kenna Martinez; individualCount: 1; sex: female; occurrenceID: 3B19C356-7EE5-5CC2-A416-A90295DCA2B1; **Taxon:** scientificName: Basicerosconjugans; kingdom: Animalia; phylum: Arthropoda; class: Insecta; order: Hymenoptera; family: Formicidae; genus: Basiceros; specificEpithet: conjugans; scientificNameAuthorship: Brown, 1974; **Location:** continent: South America; country: Colombia; countryCode: CO; stateProvince: Caquetá; county: Florencia; locality: Vda. La Paz, Fca. El Jardín; verbatimElevation: 640 m; locationRemarks: Collected in leaf Litter; verbatimCoordinates: 01°43'39.7"N 75°37'11.5"W; verbatimCoordinateSystem: WGS84; **Event:** samplingProtocol: Monolite / TSBF; eventDate: 2023-02-20; **Record Level:** language: es; collectionID: RNC:270; institutionCode: Universidad de la Amazonia (UDLA); basisOfRecord: PreservedSpecimen

#### Diagnosis

**Figure 6.**
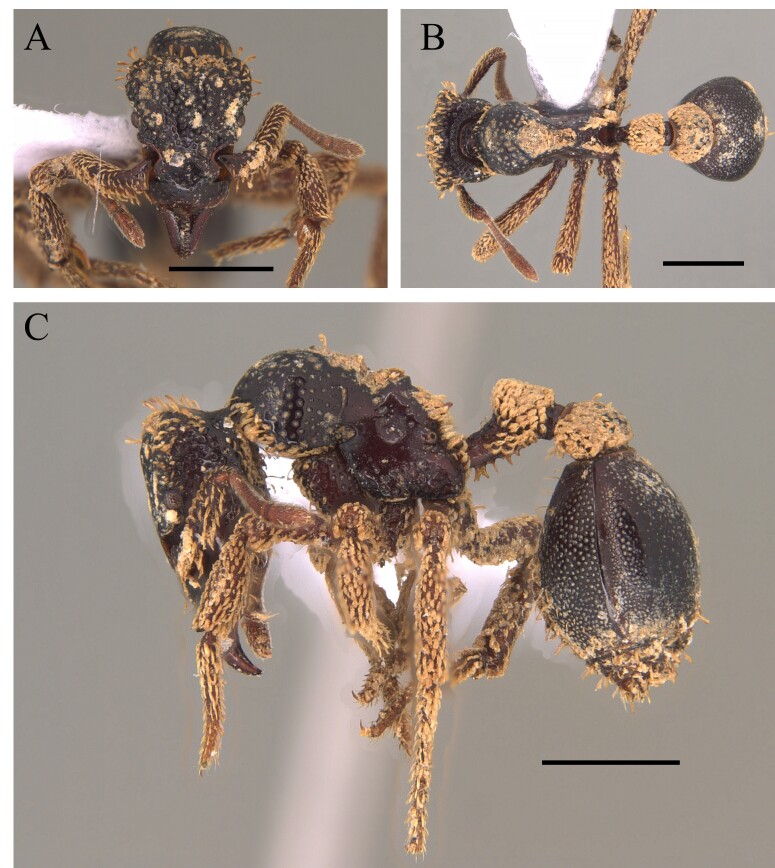
*Basicerosconjugans* worker (LEUA-00000061876) **A** head in frontal view; **B** body in dorsal view; **C** body in lateral view. Scale bars: 1.0 mm (A, B, C).

This species is recognised within the genus *Basiceros* Schulz, 1906 by the following characteristics: head almost as broad as long, occipital lobes rounded with the relatively straight with a narrow mesal concavity, without presence of continuous crest; petiolar node and postpetiole completely covered with dense pilosity; petiolar node well developed and, in dorsal view, sub-rectangular; petiole with ventral carina with many developed teeth, of different shapes; first gastral tergite with few specialised hairs on its most posterior margin, those longer than basilar pilosity (Fig. 6).

#### Distribution

This species has few records in the country, limited to the Departments of Amazonas, Meta and Nariño (Probst and Brandão 2022). It is recorded here for the first time in the Department of Caquetá. In the Neotropical Region, the species has also been recorded in Ecuador, Peru and Brazil (Probst and Brandão 2022).

#### Biology

This species was collected in leaf litter in the Río Hacha Basin. Additionally, it was collected on a log in leaf litter in cloud forest, although only one worker was observed. Probst and Brandão (2022) suggest that *B.conjugans* feeds on dead arthropods. Furthermore, it is thought that they may prey on termites, a behaviour observed in other species of the genus.

### Basiceros disciger

(Mayr, 1887)

56F3F3EF-1EB5-5749-89B9-BF058FC310EB

#### Materials

- **Type status:** Other material. **Occurrence:** catalogNumber: LEUA-00000066499; recordedBy: Brandon S. Arredondo; individualCount: 1; sex: female; occurrenceID: 19B846E0-CFAC-5490-B216-E3D819CC71FD; **Taxon:** scientificName: Basicerosdisciger; kingdom: Animalia; phylum: Arthropoda; class: Insecta; order: Hymenoptera; family: Formicidae; genus: Basiceros ; specificEpithet: disciger; scientificNameAuthorship: (Mayr, 1887); **Location:** continent: South America; country: Colombia; countryCode: CO; stateProvince: Caquetá; county: Florencia; locality: Vda. Sucre, Fca. Carlos Endo; verbatimElevation: 1018 m; locationRemarks: Collected in leaf Litter; verbatimCoordinates: 01°47'33.8"N 75°39'01.3"W; verbatimCoordinateSystem: WGS84; **Event:** samplingProtocol: Winkler; eventDate: 2023-02-09; **Record Level:** language: es; collectionID: RNC:270; institutionCode: Universidad de la Amazonia (UDLA); basisOfRecord: PreservedSpecimen

#### Diagnosis

**Figure 7.**
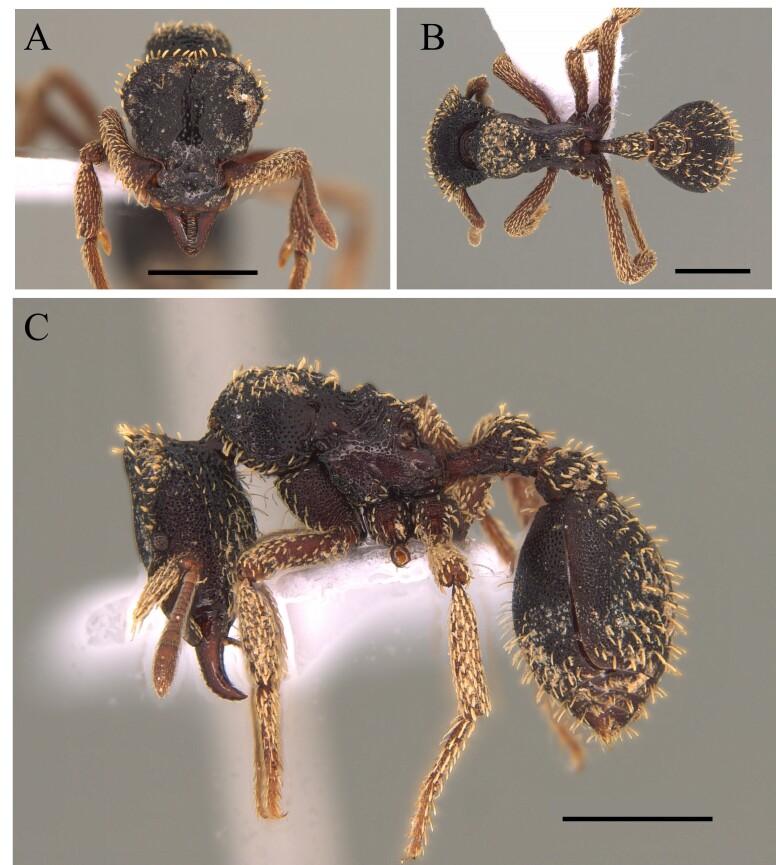
*Basicerosdisciger* worker (LEUA-00000066499) **A** head in frontal view; **B** body in dorsal view; **C** body in lateral view. Scale bars: 1.0 mm (A, B, C).

This species is recognised within the genus *Basiceros* by the following characteristics: rounded occipital margin forming a continuous elevated crest, posterior crest of the vertex emarginate medially which meets the medial convexity of the vertex (Fig. 7).

#### Distribution

This species is rarely collected. In Colombia, it has been recorded in the Departments of Meta and Norte de Santander (Probst and Brandão 2022). It is reported here for the first time in the Department of Caquetá. In the Neotropical Region, the species has also been recorded in Argentina, Brazil, Ecuador, Paraguay, Peru and Venezuela (Probst and Brandão 2022).

#### Biology

*B.disciger* is a common species in leaf-litter samples and widely distributed. A distinctive characteristic of this species is it presence in both highly conserved and degraded areas, unlike other species of the genus (Probst and Brandão 2022).

### Basiceros scambognathus

(Brown, 1949)

2E820A04-9E75-557F-8E7D-7D69D4C479CC

#### Materials

- **Type status:** Other material. **Occurrence:** catalogNumber: LEUA-00000066497; recordedBy: Brandon S. Arredondo; individualCount: 1; sex: female; occurrenceID: 06C73F41-B04B-59CF-96CB-514B60B5F3F5; **Taxon:** scientificName: Basicerosscambognathus; kingdom: Animalia; phylum: Arthropoda; class: Insecta; order: Hymenoptera; family: Formicidae; genus: Basiceros; specificEpithet: scambognathus; scientificNameAuthorship: (Brown, 1949); **Location:** continent: South America; country: Colombia; countryCode: CO; stateProvince: Caquetá; county: Florencia; locality: Universidad de la Amazonia, sede centro; verbatimElevation: 249 m; locationRemarks: Collected in leaf Litter; verbatimCoordinates: 01°36'20"N 75°36'17"W; verbatimCoordinateSystem: WGS84; **Event:** samplingProtocol: Winkler; eventDate: 2023-08-18; **Record Level:** language: es; collectionID: RNC:270; institutionCode: Universidad de la Amazonia (UDLA); basisOfRecord: PreservedSpecimen
- **Type status:** Other material. **Occurrence:** catalogNumber: LEUA-00000066498; recordedBy: Brandon S. Arredondo; individualCount: 1; sex: female; occurrenceID: D7E977A5-D3AD-5CEB-AA07-2E0ABA414D91; **Taxon:** scientificName: Basicerosscambognathus; kingdom: Animalia; phylum: Arthropoda; class: Insecta; order: Hymenoptera; family: Formicidae; genus: Basiceros; specificEpithet: scambognathus; scientificNameAuthorship: (Brown, 1949); **Location:** continent: South America; country: Colombia; countryCode: CO; stateProvince: Caquetá; county: Florencia; locality: Universidad de la Amazonia, sede centro; verbatimElevation: 249 m; locationRemarks: Collected in leaf Litter; verbatimCoordinates: 01°36'20"N 75°36'17"W; verbatimCoordinateSystem: WGS84; **Event:** samplingProtocol: Winkler; eventDate: 2023-08-18; **Record Level:** language: es; collectionID: RNC:270; institutionCode: Universidad de la Amazonia (UDLA); basisOfRecord: PreservedSpecimen

#### Diagnosis

**Figure 8.**
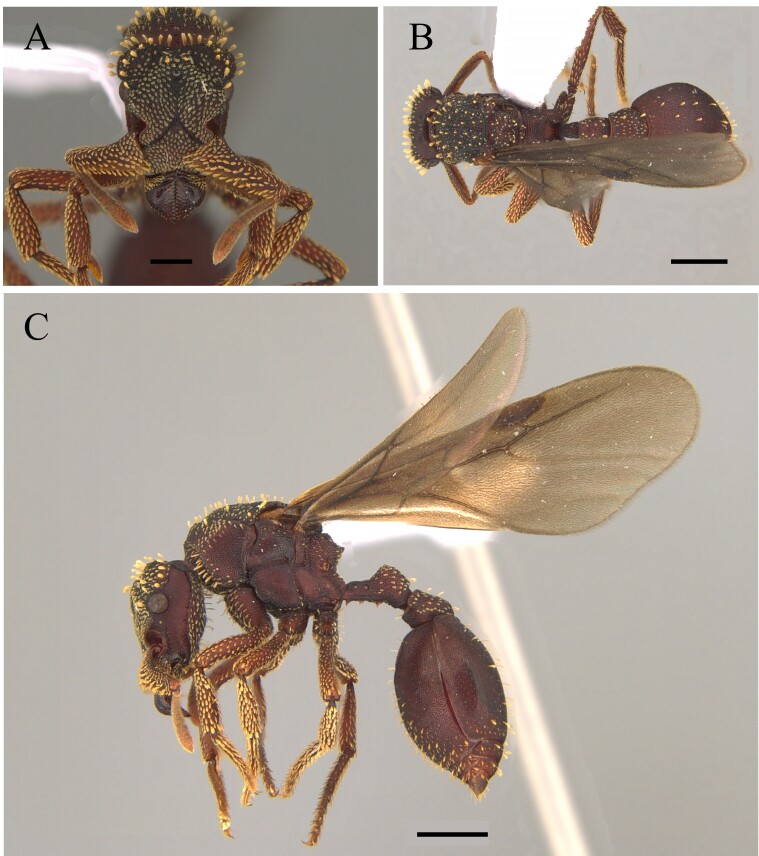
*Basicerosscambognathus* queen (LEUA-00000066497) **A** head in frontal view; **B** body in dorsal view; **C** body in lateral view. Scale bars: 1.0 mm (A, B, C).

This species is recognised within the genus *Basiceros* by the following characteristics: occipital margin strongly emarginate generating two occipital lobes, mandible subtriangular, strongly bent ventrally, outer edges straight basally; mesoanapisternum strongly depressed (Fig. 8).

#### Distribution

This species was previously recorded in the Department of Amazonas (Fernández and Palacio 1995). It is reported here for the first time in the Department of Caquetá. In the Neotropical Region, the species has also been recorded in Brazil, Peru and Venezuela (Probst and Brandão 2022).

#### Biology

According to Probst and Brandão (2022), the unusual morphology of the mandibles of the species suggests specific predation conditions. However, records on its biology are limited to those obtained by Feitosa et al. (2007); a queen was observed preying on termites under artificial conditions.

### Kempfidris inusualis

(Fernández, 2007)

BA6EC412-64F6-5E21-A358-D858CFD3CC23

#### Materials

- **Type status:** Other material. **Occurrence:** catalogNumber: LEUA-00000066487; recordedBy: Luz Albenis Villaquiran; individualCount: 1; sex: female; occurrenceID: BB3475BB-75C4-5FB1-8CC0-7F54063948B8; **Taxon:** scientificName: Kempfidrisinusualis; kingdom: Animalia; phylum: Arthropoda; class: Insecta; order: Hymenoptera; family: Formicidae; genus: Kempfidris; specificEpithet: inusualis; scientificNameAuthorship: (Fernández, 2007); **Location:** continent: South America; country: Colombia; countryCode: CO; stateProvince: Caquetá; county: Florencia; locality: Vda. La Viciosa, CIMAZ Macagual; verbatimElevation: 320 m; locationRemarks: Collected in leaf Litter; verbatimCoordinates: 01°29'46"N 75°39'19"W; verbatimCoordinateSystem: WGS84; **Event:** samplingProtocol: Monolite / TSBF; eventDate: 2023-09-12; **Record Level:** language: es; collectionID: RNC:270; institutionCode: Universidad de la Amazonia (UDLA); basisOfRecord: PreservedSpecimen

#### Diagnosis

**Figure 9.**
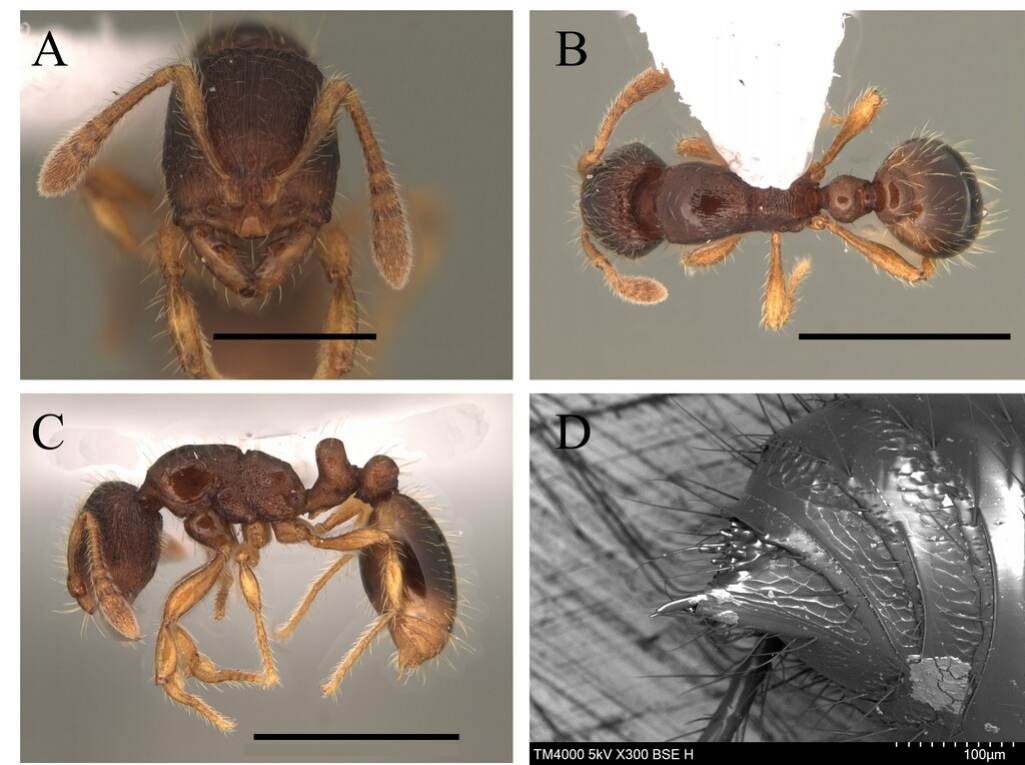
*Kempfidrisinusualis* worker (LEUA-00000066487) **A** head in frontal view; **B** body in dorsal view; **C** body in lateral view; **D** Electron microscopy of micro-cylindrical pins. Scale bars: 0.5 mm (A); 1.0 (B, C).

The genus and species can be distinguished from any other Myrmicine ant by a posteromedian portion of abdominal tergum VI and anteromedian portion of abdominal tergum VII with several minute, cylindrical micro-pegs, each bearing a hair on apex (Fig. 9) (Fernández et al. 2014).

#### Distribution

*Kempfidrisinusualis* is a Neotropical species restricted to the Amazon rainforest. In Colombia, it is only known from the Department of Amazonas (Castro et al. 2018). It is reported here for the first time in the Department of Caquetá. In the Neotropical Region, the species has also been recorded in Ecuador, Peru, Brazil and Venezuela (Fernández et al. 2014, Camacho and Feitosa 2016).

#### Biology

This species was collected in the leaf litter in secondary forest near a fallen log in an Andean-Amazonian transition zone in the foothills of Amazonia.

#### Notes

Fernández (2007) mentions that the morphological variability of the species is low, finding a variation in colouration, with more than one difference in the dorsal face of the propodeum and in the propodeal ridges, attributing this variation to the geographical distribution of the species.

### Lachnomyrmex pilosus

(Weber, 1950)

419BD0AE-6E24-5994-8B50-885BAC38E740

#### Materials

- **Type status:** Other material. **Occurrence:** catalogNumber: LEUA-00000066489; recordedBy: Fernando Celis; individualCount: 1; sex: female; occurrenceID: BBDB39C8-D45C-573F-B971-66C47CB996AE; **Taxon:** scientificName: Lachnomyrmexpilosus; kingdom: Animalia; phylum: Arthropoda; class: Insecta; order: Hymenoptera; family: Formicidae; genus: Lachnomyrmex; specificEpithet: pilosus; scientificNameAuthorship: (Weber, 1950); **Location:** continent: South America; country: Colombia; countryCode: CO; stateProvince: Caquetá; county: Florencia; locality: Vda. Sucre, Fca. La Fortaleza; verbatimElevation: 1266 m; locationRemarks: Collected in leaf Litter; verbatimCoordinates: 01°48'22.1"N 75°39'12.7"W; verbatimCoordinateSystem: WGS84; **Event:** samplingProtocol: Winkler; eventDate: 2023-08-12; **Record Level:** language: es; collectionID: RNC:270; institutionCode: Universidad de la Amazonia (UDLA); basisOfRecord: PreservedSpecimen
- **Type status:** Other material. **Occurrence:** catalogNumber: LEUA-00000066490; recordedBy: Fernando Celis; individualCount: 1; sex: female; occurrenceID: 2E423463-C27D-5D18-9517-C51E6789FAB1; **Taxon:** scientificName: Lachnomyrmexpilosus; kingdom: Animalia; phylum: Arthropoda; class: Insecta; order: Hymenoptera; family: Formicidae; genus: Lachnomyrmex; specificEpithet: pilosus; scientificNameAuthorship: (Weber, 1950); **Location:** continent: South America; country: Colombia; countryCode: CO; stateProvince: Caquetá; county: Florencia; locality: Vda. Sucre, Fca. La Fortaleza; verbatimElevation: 1266 m; locationRemarks: Collected in leaf Litter; verbatimCoordinates: 01°48'22.1"N 75°39'12.7"W; verbatimCoordinateSystem: WGS84; **Event:** samplingProtocol: Winkler; eventDate: 2023-08-12; **Record Level:** language: es; collectionID: RNC:270; institutionCode: Universidad de la Amazonia (UDLA); basisOfRecord: PreservedSpecimen
- **Type status:** Other material. **Occurrence:** catalogNumber: LEUA-00000066488; recordedBy: Yenifer Gutierrez; individualCount: 1; sex: female; occurrenceID: C7D8443B-C1D1-5884-9010-081D0C6F5090; **Taxon:** scientificName: Lachnomyrmexpilosus; kingdom: Animalia; phylum: Arthropoda; class: Insecta; order: Hymenoptera; family: Formicidae; genus: Lachnomyrmex; specificEpithet: pilosus; scientificNameAuthorship: (Weber, 1950); **Location:** continent: South America; country: Colombia; countryCode: CO; stateProvince: Caquetá; county: Florencia; locality: Av.Caraño, Vía Florencia-Suaza; verbatimElevation: 1579 m; locationRemarks: Collected in leaf Litter; verbatimCoordinates: 01°43'24.4"N 75°43'10"W; verbatimCoordinateSystem: WGS84; **Event:** samplingProtocol: Winkler; eventDate: 2023-09-30; **Record Level:** language: es; collectionID: RNC:270; institutionCode: Universidad de la Amazonia (UDLA); basisOfRecord: PreservedSpecimen

#### Diagnosis

**Figure 10.**
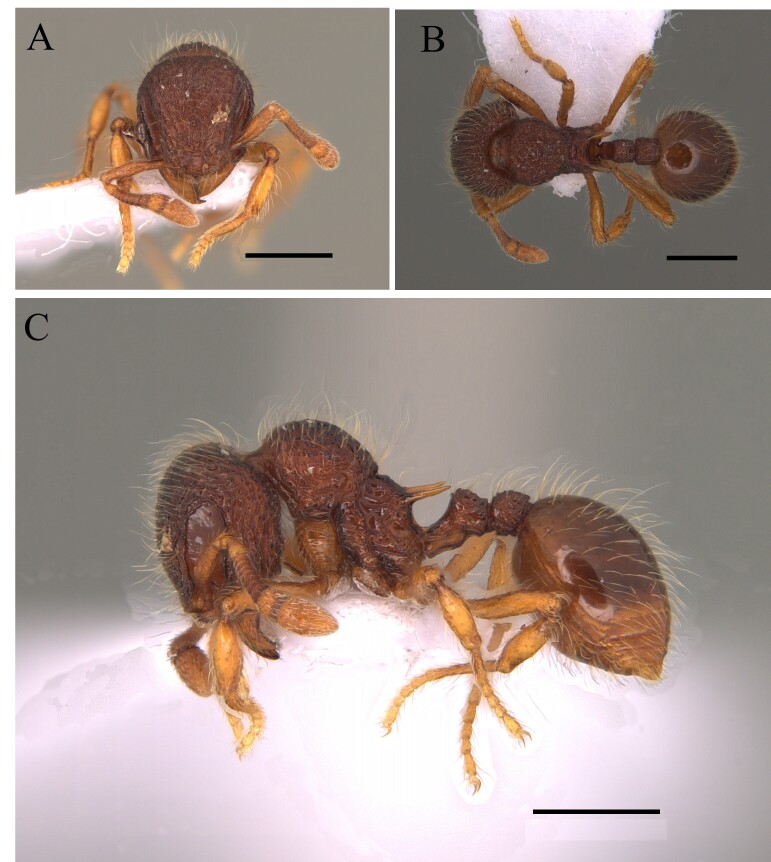
*Lachnomyrmexpilosus* worker (LEUA-00000066488) **A** head in frontal view; **B** body in dorsal view; **C** body in lateral view. Scale bars: 0.5 mm (A, B, C).

This uncommon species is recognised by the following characteristics: inner surface of antennal scrobes and first gastral tergum smooth and shiny; body covered by long, flexible hairs; mesosoma covered fine, short striation; in lateral view, promesonotum strongly convex, superior to level of propodeum; propodeal spines less than twice as long as distance between their bases. The specimens collected in the eastern Amazon Basin are smaller than the Brazilian populations (Fig. 10) (Feitosa and Brandão 2008).

#### Distribution

This is a Neotropical species reported from Brazil, Ecuador, Peru and Venezuela and is little known in Colombia with records from Amazonas, Meta and Nariño (Feitosa and Brandão 2008). It is reported here for the first time in the Department of Caquetá.

#### Biology

Species common in the leaf litter of humid forests, distributed from 200 to 1400 m elev.

### Lenomyrmex inusitatus

(Fernández, 2001)

1458DB6F-94EB-501C-A48C-7355C8BD23B8

#### Materials

- **Type status:** Other material. **Occurrence:** catalogNumber: LEUA-00000066496; recordedBy: Yenifer Gutierrez; individualCount: 1; sex: female; occurrenceID: C6C8D14D-651B-577D-990C-B7F436758C3D; **Taxon:** scientificName: Lenomyrmexinusitatus; kingdom: Animalia; phylum: Arthropoda; class: Insecta; order: Hymenoptera; family: Formicidae; genus: Lenomyrmex; specificEpithet: inusitatus; scientificNameAuthorship: (Fernández, 2001); **Location:** continent: South America; country: Colombia; countryCode: CO; stateProvince: Caquetá; county: Florencia; locality: Av.Caraño, Vía Florencia-Suaza; verbatimElevation: 1375 m; locationRemarks: Collected in leaf Litter; verbatimCoordinates: 01°42'34.3"N 75°43'5.9"W; verbatimCoordinateSystem: WGS84; **Event:** samplingProtocol: Winkler; eventDate: 2023-09-30; **Record Level:** language: es; collectionID: RNC:270; institutionCode: Universidad de la Amazonia (UDLA); basisOfRecord: PreservedSpecimen

#### Diagnosis

**Figure 11.**
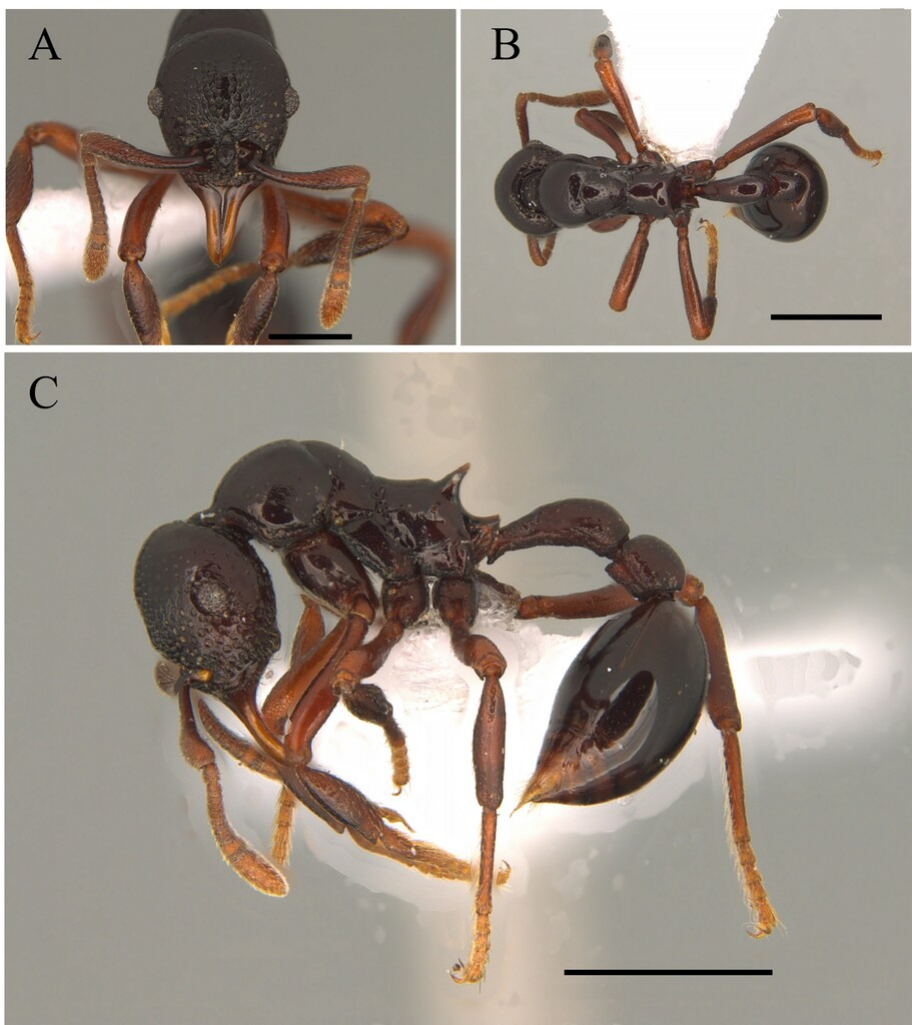
*Lenomyrmexinusitatus* worker (LEUA-00000066496) **A** head in frontal view; **B** body in dorsal view; **C** body in lateral view. Scale bars: 0.5 mm (A); 1.0 (B, C).

This species can be recognised by its mesosoma smooth and shiny, with no erect hairs; propodeum with a pair of acute and well-defined spines; head foveolate, with median longitudinal striae (Fig. 11) (Fernández and Serna 2019).

#### Distribution

This is the first record of the genus and species for the Amazon Region. The species was previously known from southern Colombia (Delsinne and Fernández 2011) and Ecuador (Salazar et al. 2015). This finding highlights the importance of studying ants in transition zones, as valuable information can be obtained about the distribution of species considered primarily Andean.

#### Biology

Unusual species, rarely collected. According to Delsinne and Fernández (2011), *L.inusitatus* inhabits mainly leaf litter and modified mandibles may be related to their being specialised hunter ants, although their prey is not known.

### Mycetomoellerius farinosus

(Emery, 1894)

81079C65-E1A1-5F6D-8340-A58B2E50D266

#### Materials

- **Type status:** Other material. **Occurrence:** catalogNumber: LEUA-00000061931; recordedBy: Brandon S. Arredondo; individualCount: 1; sex: female; occurrenceID: 45CD220C-00A3-5480-ACA4-3AF8CD70C8E0; **Taxon:** scientificName: Mycetomoelleriusfarinosus; kingdom: Animalia; phylum: Arthropoda; class: Insecta; order: Hymenoptera; family: Formicidae; genus: Mycetomoellerius; specificEpithet: farinosus; scientificNameAuthorship: (Emery, 1894); **Location:** continent: South America; country: Colombia; countryCode: CO; stateProvince: Caquetá; county: Florencia; locality: Vda. Villaraz, Fca. El Triunfo; verbatimElevation: 806 m; locationRemarks: From 0 cm to 10 cm deep in the ground; verbatimCoordinates: 01°43'18.3"N 75°40'06.5"W; verbatimCoordinateSystem: WGS84; **Event:** samplingProtocol: Monolite / TSBF; eventDate: 2023-03-14; **Record Level:** language: es; collectionID: RNC:270; institutionCode: Universidad de la Amazonia (UDLA); basisOfRecord: PreservedSpecimen
- **Type status:** Other material. **Occurrence:** catalogNumber: LEUA-00000061965; recordedBy: Brandon S. Arredondo; individualCount: 1; sex: female; occurrenceID: 20A1D378-29BA-5ACA-A802-FD17DED0D453; **Taxon:** scientificName: Mycetomoelleriusfarinosus; kingdom: Animalia; phylum: Arthropoda; class: Insecta; order: Hymenoptera; family: Formicidae; genus: Mycetomoellerius; specificEpithet: farinosus; scientificNameAuthorship: (Emery, 1894); **Location:** continent: South America; country: Colombia; countryCode: CO; stateProvince: Caquetá; county: Florencia; locality: Vda. Villaraz, Fca. El Triunfo; verbatimElevation: 806 m; locationRemarks: Collected in leaf Litter; verbatimCoordinates: 01°43'18.3"N 75°40'06.5"W; verbatimCoordinateSystem: WGS84; **Event:** samplingProtocol: Monolite / TSBF; eventDate: 2023-03-14; **Record Level:** language: es; collectionID: RNC:270; institutionCode: Universidad de la Amazonia (UDLA); basisOfRecord: PreservedSpecimen
- **Type status:** Other material. **Occurrence:** catalogNumber: LEUA-00000061972; recordedBy: Brandon S. Arredondo; individualCount: 1; sex: female; occurrenceID: 83673E1E-C0EA-5E46-B69F-992AEE4E4919; **Taxon:** scientificName: Mycetomoelleriusfarinosus; kingdom: Animalia; phylum: Arthropoda; class: Insecta; order: Hymenoptera; family: Formicidae; genus: Mycetomoellerius; specificEpithet: farinosus; scientificNameAuthorship: (Emery, 1894); **Location:** continent: South America; country: Colombia; countryCode: CO; stateProvince: Caquetá; county: Florencia; locality: Vda. Villaraz, Fca. El Triunfo; verbatimElevation: 806 m; locationRemarks: From 20 cm to 30 cm deep in the ground; verbatimCoordinates: 01°43'18.3"N 75°40'06.5"W; verbatimCoordinateSystem: WGS84; **Event:** samplingProtocol: Monolite / TSBF; eventDate: 2023-03-14; **Record Level:** language: es; collectionID: RNC:270; institutionCode: Universidad de la Amazonia (UDLA); basisOfRecord: PreservedSpecimen
- **Type status:** Other material. **Occurrence:** catalogNumber: LEUA-00000061973; recordedBy: Brandon S. Arredondo; individualCount: 1; sex: female; occurrenceID: 40C151DA-F972-577D-A956-D9AD55EF32BD; **Taxon:** scientificName: Mycetomoelleriusfarinosus; kingdom: Animalia; phylum: Arthropoda; class: Insecta; order: Hymenoptera; family: Formicidae; genus: Mycetomoellerius; specificEpithet: farinosus; scientificNameAuthorship: (Emery, 1894); **Location:** continent: South America; country: Colombia; countryCode: CO; stateProvince: Caquetá; county: Florencia; locality: Vda. Villaraz, Fca. El Triunfo; verbatimElevation: 806 m; locationRemarks: From 20 cm to 30 cm deep in the ground; verbatimCoordinates: 01°43'18.3"N 75°40'06.5"W; verbatimCoordinateSystem: WGS84; **Event:** samplingProtocol: Monolite / TSBF; eventDate: 2023-03-14; **Record Level:** language: es; collectionID: RNC:270; institutionCode: Universidad de la Amazonia (UDLA); basisOfRecord: PreservedSpecimen
- **Type status:** Other material. **Occurrence:** catalogNumber: LEUA-00000061980; recordedBy: Brandon S. Arredondo; individualCount: 1; sex: female; occurrenceID: 45D4F552-CA7E-547B-AD68-82CF10AEA6EA; **Taxon:** scientificName: Mycetomoelleriusfarinosus; kingdom: Animalia; phylum: Arthropoda; class: Insecta; order: Hymenoptera; family: Formicidae; genus: Mycetomoellerius; specificEpithet: farinosus; scientificNameAuthorship: (Emery, 1894); **Location:** continent: South America; country: Colombia; countryCode: CO; stateProvince: Caquetá; county: Florencia; locality: Vda. Villaraz, Fca. El Triunfo; verbatimElevation: 806 m; locationRemarks: Collected in leaf Litter; verbatimCoordinates: 01°43'18.3"N 75°40'06.5"W; verbatimCoordinateSystem: WGS84; **Event:** samplingProtocol: Monolite / TSBF; eventDate: 2023-03-14; **Record Level:** language: es; collectionID: RNC:270; institutionCode: Universidad de la Amazonia (UDLA); basisOfRecord: PreservedSpecimen
- **Type status:** Other material. **Occurrence:** catalogNumber: LEUA-00000062142; recordedBy: Brandon S. Arredondo; individualCount: 1; sex: female; occurrenceID: 3979AAE2-8CC2-5D33-B0E1-EB151C2388FD; **Taxon:** scientificName: Mycetomoelleriusfarinosus; kingdom: Animalia; phylum: Arthropoda; class: Insecta; order: Hymenoptera; family: Formicidae; genus: Mycetomoellerius; specificEpithet: farinosus; scientificNameAuthorship: (Emery, 1894); **Location:** continent: South America; country: Colombia; countryCode: CO; stateProvince: Caquetá; county: Florencia; locality: Vda. Villaraz, Fca. El Triunfo; verbatimElevation: 806 m; locationRemarks: From 10 cm to 20 cm deep in the ground; verbatimCoordinates: 01°43'18.3"N 75°40'06.5"W; verbatimCoordinateSystem: WGS84; **Event:** samplingProtocol: Monolite / TSBF; eventDate: 2023-03-14; **Record Level:** language: es; collectionID: RNC:270; institutionCode: Universidad de la Amazonia (UDLA); basisOfRecord: PreservedSpecimen
- **Type status:** Other material. **Occurrence:** catalogNumber: LEUA-00000062143; recordedBy: Brandon S. Arredondo; individualCount: 1; sex: female; occurrenceID: 72E7280F-501C-5502-B7B9-868028082309; **Taxon:** scientificName: Mycetomoelleriusfarinosus; kingdom: Animalia; phylum: Arthropoda; class: Insecta; order: Hymenoptera; family: Formicidae; genus: Mycetomoellerius; specificEpithet: farinosus; scientificNameAuthorship: (Emery, 1894); **Location:** continent: South America; country: Colombia; countryCode: CO; stateProvince: Caquetá; county: Florencia; locality: Vda. Villaraz, Fca. El Triunfo; verbatimElevation: 806 m; locationRemarks: From 10 cm to 20 cm deep in the ground; verbatimCoordinates: 01°43'18.3"N 75°40'06.5"W; verbatimCoordinateSystem: WGS84; **Event:** samplingProtocol: Monolite / TSBF; eventDate: 2023-03-14; **Record Level:** language: es; collectionID: RNC:270; institutionCode: Universidad de la Amazonia (UDLA); basisOfRecord: PreservedSpecimen
- **Type status:** Other material. **Occurrence:** catalogNumber: LEUA-00000062144; recordedBy: Brandon S. Arredondo; individualCount: 1; sex: female; occurrenceID: E1E84BFE-4823-590C-B182-FC1AA6277E4B; **Taxon:** scientificName: Mycetomoelleriusfarinosus; kingdom: Animalia; phylum: Arthropoda; class: Insecta; order: Hymenoptera; family: Formicidae; genus: Mycetomoellerius; specificEpithet: farinosus; scientificNameAuthorship: (Emery, 1894); **Location:** continent: South America; country: Colombia; countryCode: CO; stateProvince: Caquetá; county: Florencia; locality: Vda. Villaraz, Fca. El Triunfo; verbatimElevation: 806 m; locationRemarks: From 10 cm to 20 cm deep in the ground; verbatimCoordinates: 01°43'18.3"N 75°40'06.5"W; verbatimCoordinateSystem: WGS84; **Event:** samplingProtocol: Monolite / TSBF; eventDate: 2023-03-14; **Record Level:** language: es; collectionID: RNC:270; institutionCode: Universidad de la Amazonia (UDLA); basisOfRecord: PreservedSpecimen
- **Type status:** Other material. **Occurrence:** catalogNumber: LEUA-00000062145; recordedBy: Brandon S. Arredondo; individualCount: 1; sex: female; occurrenceID: 11BCAA12-5675-5A07-BDC8-F0E281D3217C; **Taxon:** scientificName: Mycetomoelleriusfarinosus; kingdom: Animalia; phylum: Arthropoda; class: Insecta; order: Hymenoptera; family: Formicidae; genus: Mycetomoellerius; specificEpithet: farinosus; scientificNameAuthorship: (Emery, 1894); **Location:** continent: South America; country: Colombia; countryCode: CO; stateProvince: Caquetá; county: Florencia; locality: Vda. Villaraz, Fca. El Triunfo; verbatimElevation: 806 m; locationRemarks: From 10 cm to 20 cm deep in the ground; verbatimCoordinates: 01°43'18.3"N 75°40'06.5"W; verbatimCoordinateSystem: WGS84; **Event:** samplingProtocol: Monolite / TSBF; eventDate: 2023-03-14; **Record Level:** language: es; collectionID: RNC:270; institutionCode: Universidad de la Amazonia (UDLA); basisOfRecord: PreservedSpecimen

#### Diagnosis

**Figure 12.**
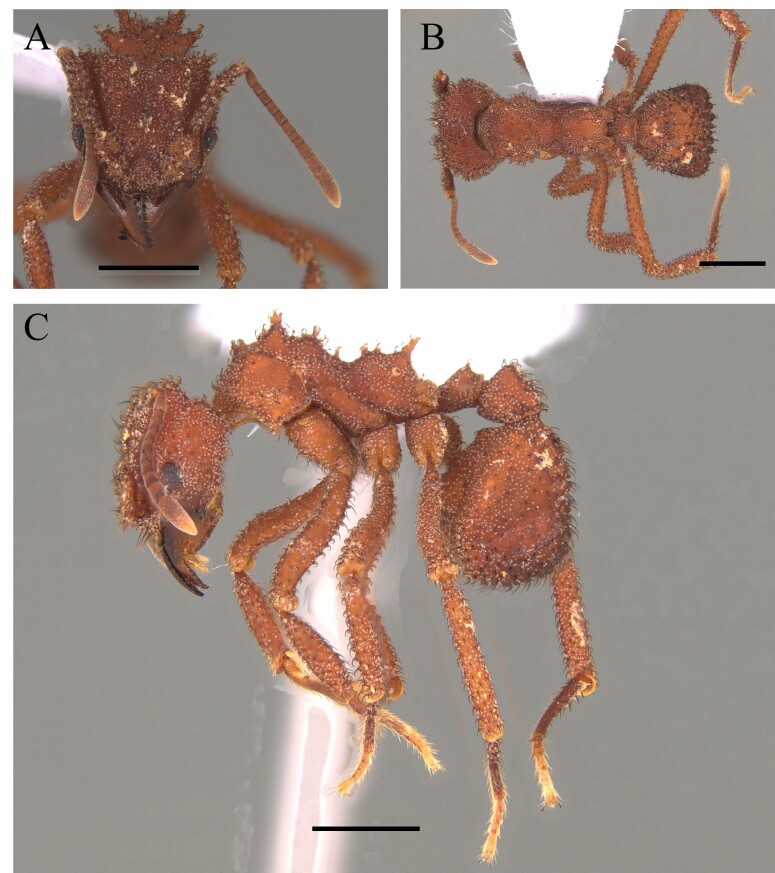
*Mycetomoelleriusfarinosus* worker (LEUA-00000061972) **A** head in frontal view; **B** body in dorsal view; **C** body in lateral view. Scale bars: 1.0 mm (A, B, C).

This species is known for having the first gastral tergite depressed above, compressed on the sides, sub-truncated and gibbous behind and body covered with white scales along the hooked hairs (Fig. 12).

#### Distribution

*Mycetomoelleriusfarinosus* is a Neotropical species distributed in the Amazon rainforest (Ryder et al. 2010b). This is the first record of the species in Colombia. In the Neotropical Region, the species has also been recorded in Brazil, Ecuador, Peru, Guyana, Suriname, French Guiana and Venezuela (Mayhe-Nunes and Jaffe 1998, Silva et al. 2004, Youngsteadt et al. 2009)

#### Biology

*Mycetomoelleriusfarinosus* is a species commonly present in leaf litter in the Andean-Amazonian transition zone of the Hacha River Basin (Caquetá).

### Oxyepoecus ephippiatus

Albuquerque & Brandão, 2004

9A2B62F7-D971-5427-9519-DE2F11A111BC

#### Materials

- **Type status:** Other material. **Occurrence:** catalogNumber: LEUA-00000066491; recordedBy: Kenna Martinez; individualCount: 1; sex: female; occurrenceID: 68EE000B-4A46-5F36-9032-80084D733F2D; **Taxon:** scientificName: Oxyepoecusephippiatus; kingdom: Animalia; phylum: Arthropoda; class: Insecta; order: Hymenoptera; family: Formicidae; genus: Oxyepoecus ; specificEpithet: ephippiatus; scientificNameAuthorship: Albuquerque & Brandão, 2004; **Location:** continent: South America; country: Colombia; countryCode: CO; stateProvince: Caquetá; county: Florencia; locality: Vda. San Luis, Fca. El Carmen 1; verbatimElevation: 433 m; locationRemarks: Collected in leaf Litter; verbatimCoordinates: 01°40'34.2"N 75°37'39.2"W; verbatimCoordinateSystem: WGS84; **Event:** samplingProtocol: Monolite / TSBF; eventDate: 2022-12-20; **Record Level:** language: es; collectionID: RNC:270; institutionCode: Universidad de la Amazonia (UDLA); basisOfRecord: PreservedSpecimen

#### Diagnosis

**Figure 13.**
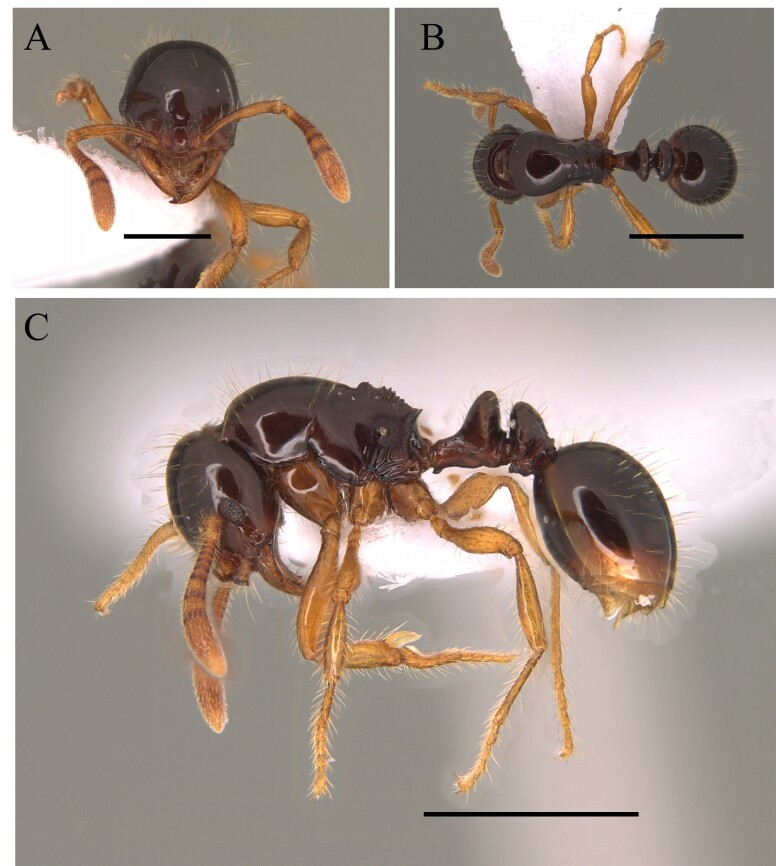
*Oxyepoecusephippiatus* worker (LEUA-00000066491) **A** head in frontal view; **B** body in dorsal view; **C** body in lateral view. Scale bars: 0.5 mm (A); 1.0 (B, C).

This species is recognised by the following characteristics: dorsum of mesosoma smooth and shiny, irregularly reticulate sculpture absent; length of head not clearly greater than width; pronotum convex and rounded above, propodeum and metapleuron with well-marked costulae; propodeum saddle-shaped; posterior border of the katepisternum and posterior face of the postpetiole smooth and shining; petiole anteroposteriorly compressed, scale-like (Fig. 13) (Albuquerque and Brandão 2004).

#### Distribution

This species is rarely collected, with only a few records from the Amazon rainforest (Albuquerque and Brandão 2004). This is the first report of the species in Colombia and the second report of the genus in the country, which was previously known from only a single record in the Department of Meta. In the Neotropical Region, the species has also been recorded in Brazil, Ecuador and French Guiana (Albuquerque and Brandão 2004, Salazar et al. 2015, Franco et al. 2019).

#### Biology

The only specimen of *Oxyepoecusephippiatus* was collected in leaf litter in the Andean-Amazonian transition zone, in the lower cloud forest of the Hacha River Basin.

### Stegomyrmex manni

Smith, 1946

02A25171-FCF6-5F6D-B302-39071A8B975A

#### Materials

- **Type status:** Other material. **Occurrence:** catalogNumber: LEUA-00000066492; recordedBy: Kenna Martinez; individualCount: 1; sex: female; occurrenceID: D985C1CE-1A4C-5664-A67E-69FCF2D58527; **Taxon:** scientificName: Stegomyrmexmanni; kingdom: Animalia; phylum: Arthropoda; class: Insecta; order: Hymenoptera; family: Formicidae; genus: Stegomyrmex; specificEpithet: manni; scientificNameAuthorship: Smith, 1946; **Location:** continent: South America; country: Colombia; countryCode: CO; stateProvince: Caquetá; county: Florencia; locality: Vda. El Quindío, Fca. La Esperanza; verbatimElevation: 556 m; locationRemarks: Collected in leaf Litter; verbatimCoordinates: 01°42'23.9"N 75°38'40.0"W; verbatimCoordinateSystem: WGS84; **Event:** samplingProtocol: Monolite / TSBF; eventDate: 2023-05-09; **Record Level:** language: es; collectionID: RNC:270; institutionCode: Universidad de la Amazonia (UDLA); basisOfRecord: PreservedSpecimen

#### Diagnosis

**Figure 14.**
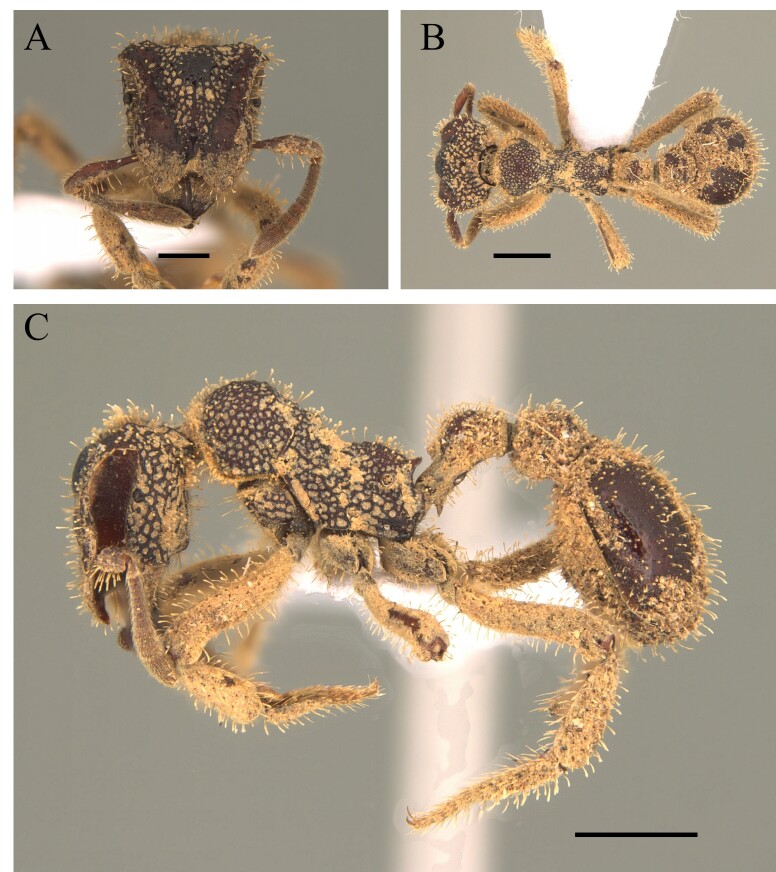
*Stegomyrmexmanni* worker (LEUA-00000066492) **A** head in frontal view; **B** body in dorsal view; **C** body in lateral view. Scale bars: 0.5 mm (A); 1.0 mm (B, C).

This species is recognised by the following characteristics: Head subtrapezoidal with angulated occipital corners; frontal lobes markedly expanded laterally and anteriorly, projecting over the clypeus and part of the mandibles; antennal scrobes deep, completely covered by lateral expansions of frontal lobes; promesonotum continuously rounded in profile; metanotal groove wide and shallow; propodeal spines short and acute; tegument hard with sculpture and coarse pilosity (Feitosa et al. 2008) (Fig. 14).

#### Distribution

*Stegomyrmexmanni* is a Neotropical species widely distributed in Mesoamerica, but with scarce records in South America (Feitosa et al. 2008). This is the first report of the species in Caquetá and the second report of this species in the country (Serna 2002), marking the first record of the species for the Amazon rainforest region. In the Neotropical Region, the species has also been recorded in Ecuador, French Guiana, Honduras, Nicaragua, Costa Rica and Panama (Smith 1946, Kempf 1972, Ryder et al. 2010b, Franco et al. 2019).

#### Biology

The specimen was collected using the TSBF methodology, between 0 and 10 cm depth, which would suggest that this species inhabits the soil. This record complements the observations made by Feitosa et al. (2008), where they mentioned that *S.manni* is a soil inhabitant and is collected infrequently using Winkler bags. Here, we report, for the first time, the hypogeic habits in this species.

### Strumigenys incuba

Bolton, 2000

9D4E03E0-7DB4-5D2E-B143-75DDF5F883FA

#### Materials

- **Type status:** Other material. **Occurrence:** catalogNumber: LEUA-00000061667; recordedBy: Brahyam Quimbaya; individualCount: 1; sex: female; occurrenceID: 037519A0-93C4-5100-BF63-DC459E0C0886; **Taxon:** scientificName: Strumigenysincuba; kingdom: Animalia; phylum: Arthropoda; class: Insecta; order: Hymenoptera; family: Formicidae; genus: Strumigenys; specificEpithet: incuba; scientificNameAuthorship: Bolton, 2000; **Location:** continent: South America; country: Colombia; countryCode: CO; stateProvince: Caquetá; county: Florencia; locality: Vda. Av. Caraño, Fca. El Chorro; verbatimElevation: 739 m; locationRemarks: From 0 cm to 10 cm deep in the ground; verbatimCoordinates: 01°44'27.2"N 75°39'12.3"W; verbatimCoordinateSystem: WGS84; **Event:** samplingProtocol: Monolite / TSBF; eventDate: 2022-12-08; **Record Level:** language: es; collectionID: RNC:270; institutionCode: Universidad de la Amazonia (UDLA); basisOfRecord: PreservedSpecimen
- **Type status:** Other material. **Occurrence:** catalogNumber: LEUA-00000061668; recordedBy: Brahyam Quimbaya; individualCount: 1; sex: female; occurrenceID: C06EDC30-76A7-525C-A8EE-F601C7277272; **Taxon:** scientificName: Strumigenysincuba; kingdom: Animalia; phylum: Arthropoda; class: Insecta; order: Hymenoptera; family: Formicidae; genus: Strumigenys; specificEpithet: incuba; scientificNameAuthorship: Bolton, 2000; **Location:** continent: South America; country: Colombia; countryCode: CO; stateProvince: Caquetá; county: Florencia; locality: Vda. Av. Caraño, Fca. El Chorro; verbatimElevation: 739 m; locationRemarks: From 0 cm to 10 cm deep in the ground; verbatimCoordinates: 01°44'27.2"N 75°39'12.3"W; verbatimCoordinateSystem: WGS84; **Event:** samplingProtocol: Monolite / TSBF; eventDate: 2022-12-08; **Record Level:** language: es; collectionID: RNC:270; institutionCode: Universidad de la Amazonia (UDLA); basisOfRecord: PreservedSpecimen
- **Type status:** Other material. **Occurrence:** catalogNumber: LEUA-00000061669; recordedBy: Brahyam Quimbaya; individualCount: 1; sex: female; occurrenceID: BA418CB0-F8F8-56FB-BDDC-4F2B7EF2C128; **Taxon:** scientificName: Strumigenysincuba; kingdom: Animalia; phylum: Arthropoda; class: Insecta; order: Hymenoptera; family: Formicidae; genus: Strumigenys; specificEpithet: incuba; scientificNameAuthorship: Bolton, 2000; **Location:** continent: South America; country: Colombia; countryCode: CO; stateProvince: Caquetá; county: Florencia; locality: Vda. Av. Caraño, Fca. El Chorro; verbatimElevation: 739 m; locationRemarks: From 0 cm to 10 cm deep in the ground; verbatimCoordinates: 01°44'27.2"N 75°39'12.3"W; verbatimCoordinateSystem: WGS84; **Event:** samplingProtocol: Monolite / TSBF; eventDate: 2022-12-08; **Record Level:** language: es; collectionID: RNC:270; institutionCode: Universidad de la Amazonia (UDLA); basisOfRecord: PreservedSpecimen

#### Diagnosis

**Figure 15.**
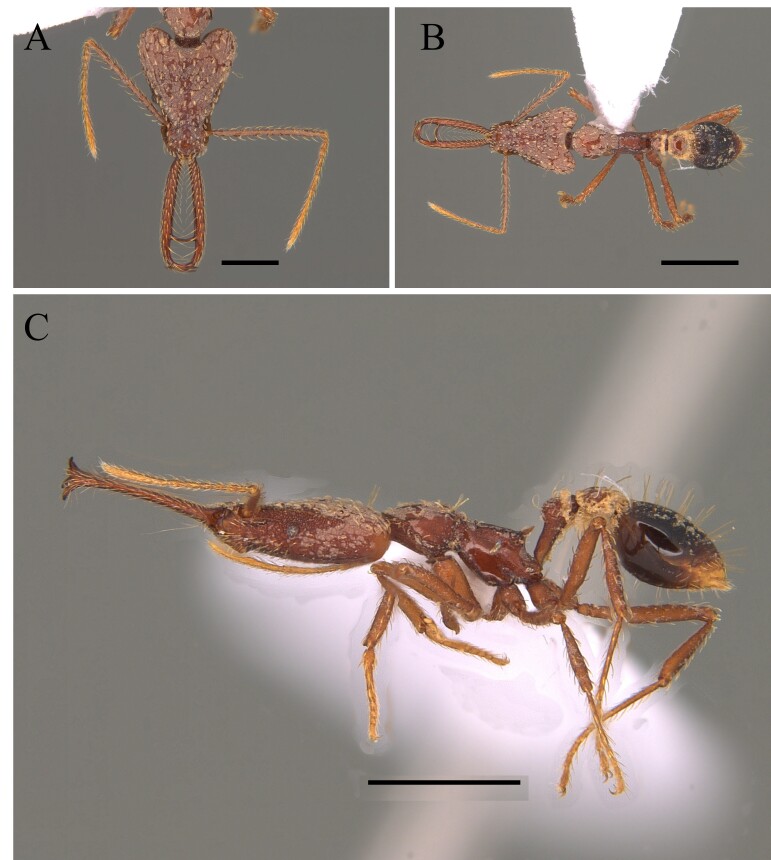
*Strumigenysincuba* worker (LEUA-00000062105) **A** head in frontal view; **B** body in dorsal view; **C** body in lateral view. Scale bars: 0.5 mm (A); 1.0 mm (B, C).

This species is recognised by having a mandible located in the middle of the anterior margin of the clypeus, without intercalary teeth, with a single proximal pre-apical tooth located near the mid-length; propodeal declivity with a narrow carina; a disc of the postpetiole and dorsum of first gastral tergite partially smooth with the presence of the basigastral carina; hairs of the first gastral tergite rigid; dorsum of the pronotum strongly rugose longitudinally; metapleural and lateral region of the propodeum smooth (Fig. 15).

#### Distribution

This species has a restricted distribution, being recorded only from Ecuador and Colombia (Ryder et al. 2010b). In Colombia, it is known from the Departments of Cauca and Putumayo (Fernández et al. 2019). It is reported here for the first time in the Department of Caquetá.

#### Biology

The specimens were collected 0 to 10 cm deep in the soil in an Andean-Amazonian transition zone.

### Strumigenys prospiciens

Emery, 1906

A2B8507F-18A3-5212-97F8-0F4CD779DC6F

#### Materials

- **Type status:** Other material. **Occurrence:** catalogNumber: LEUA-00000062105; recordedBy: Kenna Martinez; individualCount: 1; sex: female; occurrenceID: A2501D90-71C9-550A-9BAE-6F3B560DF9B6; **Taxon:** scientificName: Strumigenysprospiciens; kingdom: Animalia; phylum: Arthropoda; class: Insecta; order: Hymenoptera; family: Formicidae; genus: Strumigenys ; specificEpithet: prospiciens; scientificNameAuthorship: Emery, 1906; **Location:** continent: South America; country: Colombia; countryCode: CO; stateProvince: Caquetá; county: Florencia; locality: Vda. Av. Caraño, Fca. Buenos Aires 1; verbatimElevation: 1095 m; locationRemarks: From 0 cm to 10 cm deep in the ground; verbatimCoordinates: 01°44'06.6"N 75°40'24.6"W; verbatimCoordinateSystem: WGS84; **Event:** samplingProtocol: Monolite / TSBF; eventDate: 2023-06-01; **Record Level:** language: es; collectionID: RNC:270; institutionCode: Universidad de la Amazonia (UDLA); basisOfRecord: PreservedSpecimen

#### Diagnosis

**Figure 16.**
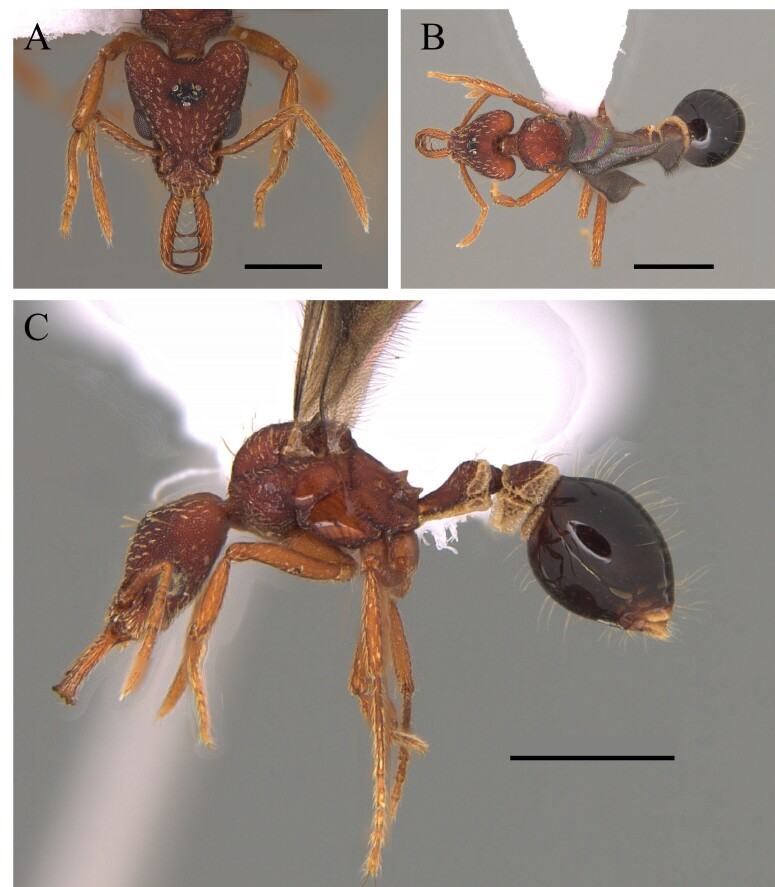
*Strumigenysprospiciens* queen (LEUA-00000062105) **A** head in frontal view; **B** body in dorsal view; **C** body in lateral view. Scale bars: 0.5 mm (A); 1.0 mm (B, C).

This species is recognised by the following characteristics: in frontal view, mandibles arising from the middle of the anterior margin of the clypeus; cephalic dorsum with a single pair of erect hairs, located posteromedially; Mesosoma in profile, propodeal declivity concave, upper and lower propodeal teeth narrowly proximal, joined by a short, broad lamella; bulla of femoral gland located dorsally in apical quarter of segment length, usually with faint oval patch-like appearance; first gastral tergite with numerous flagellate, suberect to erect hairs arising over entire tergite (Fig. 16) (Emery 1906, Bolton 2000).

#### Distribution

*Strumigenysprospiciens* is widely distributed in South America (Bolton 2000). It is reported here for the first time in Colombia. In the Neotropical Region, the species has also been recorded in Brazil, Bolivia, Ecuador, Peru, Paraguay, French Guiana and Venezuela (Lattke and Goitía 1997, Bolton 2000, Wild 2007).

#### Biology

The queen was collected 0 to 10 cm deep in the soil in an Andean-Amazonian transition zone, using TSBF sampling methodology.

### Centromyrmex gigas

Forel, 1911

BA7FAED8-6A52-5B51-8193-D53C4F761E9E

#### Materials

- **Type status:** Other material. **Occurrence:** catalogNumber: LEUA-00000061955; recordedBy: Brahyam Quimbaya; individualCount: 1; sex: female; occurrenceID: BE32A364-4DC5-566E-9014-024D0601658C; **Taxon:** scientificName: Centromyrmexgigas; kingdom: Animalia; phylum: Arthropoda; class: Insecta; order: Hymenoptera; family: Formicidae; genus: Centromyrmex; specificEpithet: gigas; scientificNameAuthorship: Forel, 1911; **Location:** continent: South America; country: Colombia; countryCode: CO; stateProvince: Caquetá; county: Florencia; locality: Vda. Av. Caraño, Fca. La Esperanza; verbatimElevation: 604 m; locationRemarks: From 10 cm to 20 cm deep in the ground; verbatimCoordinates: 01°43'45.2"N 75°38'57.2"W; verbatimCoordinateSystem: WGS84; **Event:** samplingProtocol: Monolite / TSBF; eventDate: 2023-03-22; **Record Level:** language: es; collectionID: RNC:270; institutionCode: Universidad de la Amazonia (UDLA); basisOfRecord: PreservedSpecimen
- **Type status:** Other material. **Occurrence:** catalogNumber: LEUA-00000061956; recordedBy: Brahyam Quimbaya; individualCount: 1; sex: female; occurrenceID: A7562812-544D-55F5-883E-46ED36B479B2; **Taxon:** scientificName: Centromyrmexgigas; kingdom: Animalia; phylum: Arthropoda; class: Insecta; order: Hymenoptera; family: Formicidae; genus: Centromyrmex; specificEpithet: gigas; scientificNameAuthorship: Forel, 1911; **Location:** continent: South America; country: Colombia; countryCode: CO; stateProvince: Caquetá; county: Florencia; locality: Vda. Av. Caraño, Fca. La Esperanza; verbatimElevation: 604 m; locationRemarks: From 10 cm to 20 cm deep in the ground; verbatimCoordinates: 01°43'45.2"N 75°38'57.2"W; verbatimCoordinateSystem: WGS84; **Event:** samplingProtocol: Monolite / TSBF; eventDate: 2023-03-22; **Record Level:** language: es; collectionID: RNC:270; institutionCode: Universidad de la Amazonia (UDLA); basisOfRecord: PreservedSpecimen
- **Type status:** Other material. **Occurrence:** catalogNumber: LEUA-00000061957; recordedBy: Brahyam Quimbaya; individualCount: 1; sex: female; occurrenceID: 602014AE-F9A7-58B6-A9AE-660AC2DC431E; **Taxon:** scientificName: Centromyrmexgigas; kingdom: Animalia; phylum: Arthropoda; class: Insecta; order: Hymenoptera; family: Formicidae; genus: Centromyrmex; specificEpithet: gigas; scientificNameAuthorship: Forel, 1911; **Location:** continent: South America; country: Colombia; countryCode: CO; stateProvince: Caquetá; county: Florencia; locality: Vda. Av. Caraño, Fca. La Esperanza; verbatimElevation: 604 m; locationRemarks: From 10 cm to 20 cm deep in the ground; verbatimCoordinates: 01°43'45.2"N 75°38'57.2"W; verbatimCoordinateSystem: WGS84; **Event:** samplingProtocol: Monolite / TSBF; eventDate: 2023-03-22; **Record Level:** language: es; collectionID: RNC:270; institutionCode: Universidad de la Amazonia (UDLA); basisOfRecord: PreservedSpecimen

#### Diagnosis

**Figure 17.**
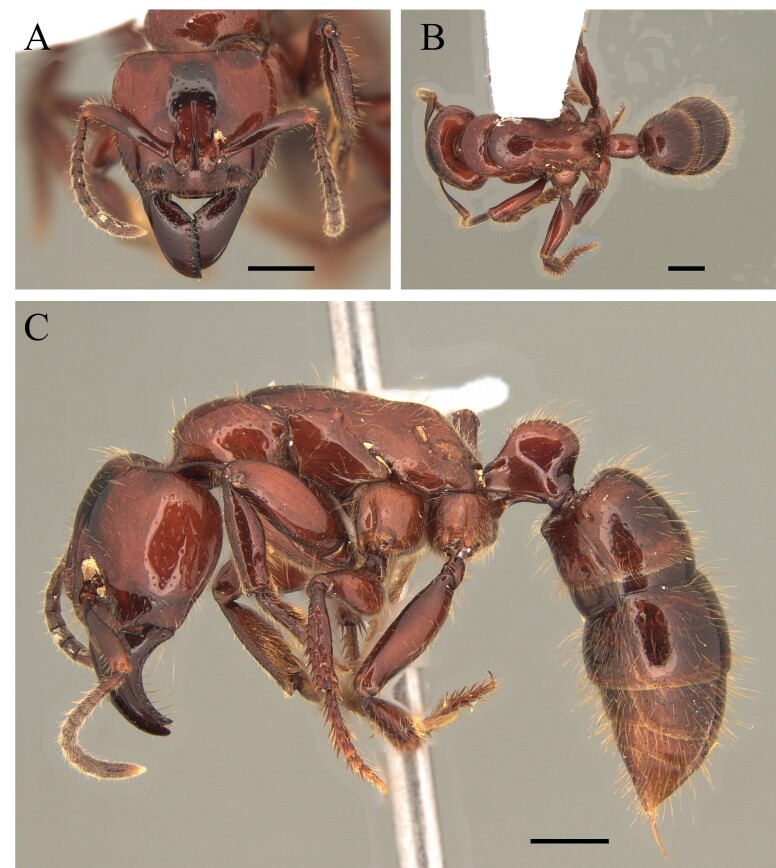
*Centromyrmexgigas* worker (LEUA-00000061955) **A** head in frontal view; **B** body in dorsal view; **C** body in lateral view. Scale bars: 1.0 mm (A, B, C).

This species is recognised by being one of the largest of the genus with clypeus without a disc mound; basal third of mandible serrated, masticatory margin of mandible forming an obtuse angle with its basal margin; petiole distinctly longer than wide, anterior face perpendicular to dorsal face of petiolar node, which is an elongate and straight petiole with a prominent subpetiolar process apically rounded to subtruncate (Fig. 17) (Forel 1911, Franco and Feitosa 2018).

#### Distribution

This is the first record of *C.gigas* in Colombia. It has been previously reported from Brazil (De Oliveira et al. 2009) and French Guiana (Franco and Feitosa 2018). This record represents one of the northernmost occurrences of this species (Souza et al. 2012, Pereira Souza et al. 2015).

#### Biology

All specimens were collected at a depth of 10 to 20 cm in cloud forest, in the Andean-Amazonian transition zone of Caquetá.

#### Notes

The collected *C.gigas* specimens varied in pilosity concerning the type specimen (CASENT0907212). Some specimens were with dispersed pilosity, but no dense areas on the head and dorsum of the mesosoma, smooth and shiny scapes with erect pilosity restricted to the apex.

### Leptogenys unistimulosa

Roger, 1863

1FAC5CA1-3D3B-5EBF-9DFB-241DF0EC6770

#### Materials

- **Type status:** Other material. **Occurrence:** catalogNumber: LEUA-00000061893; recordedBy: Brahyam Quimbaya; individualCount: 1; sex: female; occurrenceID: CEA44053-0EC6-5C8D-924E-89F0127FD28D; **Taxon:** scientificName: Leptogenysunistimulosa; kingdom: Animalia; phylum: Arthropoda; class: Insecta; order: Hymenoptera; family: Formicidae; genus: Leptogenys; specificEpithet: unistimulosa; scientificNameAuthorship: Roger, 1863; **Location:** continent: South America; country: Colombia; countryCode: CO; stateProvince: Caquetá; county: Florencia; locality: Vda. Sucre, Fca. Vista Hermosa; verbatimElevation: 1207 m; locationRemarks: Collected in leaf Litter; verbatimCoordinates: 01°48'42.4"N 75°39'48.0"W; verbatimCoordinateSystem: WGS84; **Event:** samplingProtocol: Monolite / TSBF; eventDate: 2023-03-13; **Record Level:** language: es; collectionID: RNC:270; institutionCode: Universidad de la Amazonia (UDLA); basisOfRecord: PreservedSpecimen
- **Type status:** Other material. **Occurrence:** catalogNumber: LEUA-00000061894; recordedBy: Brahyam Quimbaya; individualCount: 1; sex: female; occurrenceID: D854D143-D5EC-5962-8F14-E9DA450465B1; **Taxon:** scientificName: Leptogenysunistimulosa; kingdom: Animalia; phylum: Arthropoda; class: Insecta; order: Hymenoptera; family: Formicidae; genus: Leptogenys; specificEpithet: unistimulosa; scientificNameAuthorship: Roger, 1863; **Location:** continent: South America; country: Colombia; countryCode: CO; stateProvince: Caquetá; county: Florencia; locality: Vda. Sucre, Fca. Vista Hermosa; verbatimElevation: 1207 m; locationRemarks: Collected in leaf Litter; verbatimCoordinates: 01°48'42.4"N 75°39'48.0"W; verbatimCoordinateSystem: WGS84; **Event:** samplingProtocol: Monolite / TSBF; eventDate: 2023-03-13; **Record Level:** language: es; collectionID: RNC:270; institutionCode: Universidad de la Amazonia (UDLA); basisOfRecord: PreservedSpecimen
- **Type status:** Other material. **Occurrence:** catalogNumber: LEUA-00000061895; recordedBy: Brahyam Quimbaya; individualCount: 1; sex: female; occurrenceID: 456C2B3B-75D3-5795-899F-CC0129732487; **Taxon:** scientificName: Leptogenysunistimulosa; kingdom: Animalia; phylum: Arthropoda; class: Insecta; order: Hymenoptera; family: Formicidae; genus: Leptogenys; specificEpithet: unistimulosa; scientificNameAuthorship: Roger, 1863; **Location:** continent: South America; country: Colombia; countryCode: CO; stateProvince: Caquetá; county: Florencia; locality: Vda. Sucre, Fca. Vista Hermosa; verbatimElevation: 1207 m; locationRemarks: Collected in leaf Litter; verbatimCoordinates: 01°48'42.4"N 75°39'48.0"W; verbatimCoordinateSystem: WGS84; **Event:** samplingProtocol: Monolite / TSBF; eventDate: 2023-03-13; **Record Level:** language: es; collectionID: RNC:270; institutionCode: Universidad de la Amazonia (UDLA); basisOfRecord: PreservedSpecimen
- **Type status:** Other material. **Occurrence:** catalogNumber: LEUA-00000061896; recordedBy: Brahyam Quimbaya; individualCount: 1; sex: female; occurrenceID: 4F927D89-2564-555F-AA41-73AA7936FEAF; **Taxon:** scientificName: Leptogenysunistimulosa; kingdom: Animalia; phylum: Arthropoda; class: Insecta; order: Hymenoptera; family: Formicidae; genus: Leptogenys; specificEpithet: unistimulosa; scientificNameAuthorship: Roger, 1863; **Location:** continent: South America; country: Colombia; countryCode: CO; stateProvince: Caquetá; county: Florencia; locality: Vda. Sucre, Fca. Vista Hermosa; verbatimElevation: 1207 m; locationRemarks: Collected in leaf Litter; verbatimCoordinates: 01°48'42.4"N 75°39'48.0"W; verbatimCoordinateSystem: WGS84; **Event:** samplingProtocol: Monolite / TSBF; eventDate: 2023-03-13; **Record Level:** language: es; collectionID: RNC:270; institutionCode: Universidad de la Amazonia (UDLA); basisOfRecord: PreservedSpecimen
- **Type status:** Other material. **Occurrence:** catalogNumber: LEUA-00000061897; recordedBy: Brahyam Quimbaya; individualCount: 1; sex: female; occurrenceID: F6400497-61AD-52C3-869D-54C7D95758BF; **Taxon:** scientificName: Leptogenysunistimulosa; kingdom: Animalia; phylum: Arthropoda; class: Insecta; order: Hymenoptera; family: Formicidae; genus: Leptogenys; specificEpithet: unistimulosa; scientificNameAuthorship: Roger, 1863; **Location:** continent: South America; country: Colombia; countryCode: CO; stateProvince: Caquetá; county: Florencia; locality: Vda. Sucre, Fca. Vista Hermosa; verbatimElevation: 1207 m; locationRemarks: Collected in leaf Litter; verbatimCoordinates: 01°48'42.4"N 75°39'48.0"W; verbatimCoordinateSystem: WGS84; **Event:** samplingProtocol: Monolite / TSBF; eventDate: 2023-03-13; **Record Level:** language: es; collectionID: RNC:270; institutionCode: Universidad de la Amazonia (UDLA); basisOfRecord: PreservedSpecimen
- **Type status:** Other material. **Occurrence:** catalogNumber: LEUA-00000061898; recordedBy: Brahyam Quimbaya; individualCount: 1; sex: female; occurrenceID: 519F2CB2-3C0E-5115-8ECC-EC94B0BD4771; **Taxon:** scientificName: Leptogenysunistimulosa; kingdom: Animalia; phylum: Arthropoda; class: Insecta; order: Hymenoptera; family: Formicidae; genus: Leptogenys; specificEpithet: unistimulosa; scientificNameAuthorship: Roger, 1863; **Location:** continent: South America; country: Colombia; countryCode: CO; stateProvince: Caquetá; county: Florencia; locality: Vda. Sucre, Fca. Vista Hermosa; verbatimElevation: 1207 m; locationRemarks: Collected in leaf Litter; verbatimCoordinates: 01°48'42.4"N 75°39'48.0"W; verbatimCoordinateSystem: WGS84; **Event:** samplingProtocol: Monolite / TSBF; eventDate: 2023-03-13; **Record Level:** language: es; collectionID: RNC:270; institutionCode: Universidad de la Amazonia (UDLA); basisOfRecord: PreservedSpecimen
- **Type status:** Other material. **Occurrence:** catalogNumber: LEUA-00000061899; recordedBy: Brahyam Quimbaya; individualCount: 1; sex: female; occurrenceID: B310EF03-6282-586A-896D-F96A8851A02D; **Taxon:** scientificName: Leptogenysunistimulosa; kingdom: Animalia; phylum: Arthropoda; class: Insecta; order: Hymenoptera; family: Formicidae; genus: Leptogenys; specificEpithet: unistimulosa; scientificNameAuthorship: Roger, 1863; **Location:** continent: South America; country: Colombia; countryCode: CO; stateProvince: Caquetá; county: Florencia; locality: Vda. Sucre, Fca. Vista Hermosa; verbatimElevation: 1207 m; locationRemarks: Collected in leaf Litter; verbatimCoordinates: 01°48'42.4"N 75°39'48.0"W; verbatimCoordinateSystem: WGS84; **Event:** samplingProtocol: Monolite / TSBF; eventDate: 2023-03-13; **Record Level:** language: es; collectionID: RNC:270; institutionCode: Universidad de la Amazonia (UDLA); basisOfRecord: PreservedSpecimen
- **Type status:** Other material. **Occurrence:** catalogNumber: LEUA-00000061900; recordedBy: Brahyam Quimbaya; individualCount: 1; sex: female; occurrenceID: CA3294BF-CF52-53E7-B188-548A3EC83C48; **Taxon:** scientificName: Leptogenysunistimulosa; kingdom: Animalia; phylum: Arthropoda; class: Insecta; order: Hymenoptera; family: Formicidae; genus: Leptogenys; specificEpithet: unistimulosa; scientificNameAuthorship: Roger, 1863; **Location:** continent: South America; country: Colombia; countryCode: CO; stateProvince: Caquetá; county: Florencia; locality: Vda. Sucre, Fca. Vista Hermosa; verbatimElevation: 1207 m; locationRemarks: Collected in leaf Litter; verbatimCoordinates: 01°48'42.4"N 75°39'48.0"W; verbatimCoordinateSystem: WGS84; **Event:** samplingProtocol: Monolite / TSBF; eventDate: 2023-03-13; **Record Level:** language: es; collectionID: RNC:270; institutionCode: Universidad de la Amazonia (UDLA); basisOfRecord: PreservedSpecimen
- **Type status:** Other material. **Occurrence:** catalogNumber: LEUA-00000061901; recordedBy: Brahyam Quimbaya; individualCount: 1; sex: female; occurrenceID: 449EB475-E82D-5D35-9EAE-6E6429A6450F; **Taxon:** scientificName: Leptogenysunistimulosa; kingdom: Animalia; phylum: Arthropoda; class: Insecta; order: Hymenoptera; family: Formicidae; genus: Leptogenys; specificEpithet: unistimulosa; scientificNameAuthorship: Roger, 1863; **Location:** continent: South America; country: Colombia; countryCode: CO; stateProvince: Caquetá; county: Florencia; locality: Vda. Sucre, Fca. Vista Hermosa; verbatimElevation: 1207 m; locationRemarks: Collected in leaf Litter; verbatimCoordinates: 01°48'42.4"N 75°39'48.0"W; verbatimCoordinateSystem: WGS84; **Event:** samplingProtocol: Monolite / TSBF; eventDate: 2023-03-13; **Record Level:** language: es; collectionID: RNC:270; institutionCode: Universidad de la Amazonia (UDLA); basisOfRecord: PreservedSpecimen
- **Type status:** Other material. **Occurrence:** catalogNumber: LEUA-00000061902; recordedBy: Brahyam Quimbaya; individualCount: 1; sex: female; occurrenceID: E8860E3F-A096-596A-A5A9-77A84BDD0C11; **Taxon:** scientificName: Leptogenysunistimulosa; kingdom: Animalia; phylum: Arthropoda; class: Insecta; order: Hymenoptera; family: Formicidae; genus: Leptogenys; specificEpithet: unistimulosa; scientificNameAuthorship: Roger, 1863; **Location:** continent: South America; country: Colombia; countryCode: CO; stateProvince: Caquetá; county: Florencia; locality: Vda. Sucre, Fca. Vista Hermosa; verbatimElevation: 1207 m; locationRemarks: Collected in leaf Litter; verbatimCoordinates: 01°48'42.4"N 75°39'48.0"W; verbatimCoordinateSystem: WGS84; **Event:** samplingProtocol: Monolite / TSBF; eventDate: 2023-03-13; **Record Level:** language: es; collectionID: RNC:270; institutionCode: Universidad de la Amazonia (UDLA); basisOfRecord: PreservedSpecimen

#### Diagnosis

**Figure 18.**
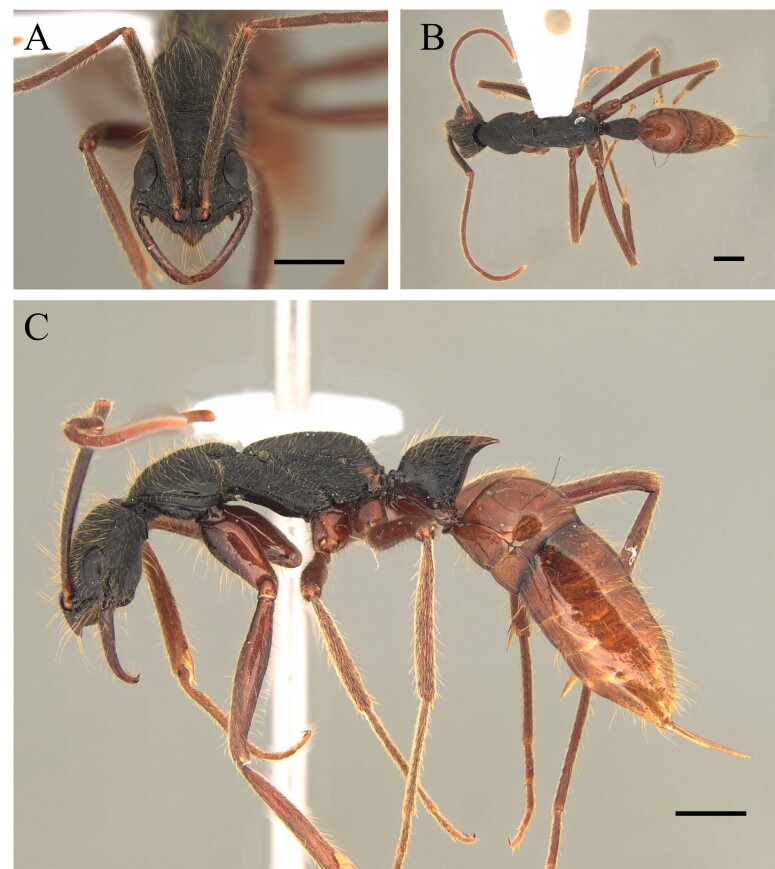
*Leptogenysunistimulosa* worker (LEUA-00000061897) **A** head in frontal view; **B** body in dorsal view; **C** body in lateral view. Scale bars: 1.0 mm (A, B, C).

This species is recognised by having, in frontal view, inner basal margin of mandible markedly separated from anterior margin of clypeus (closed mandibles); hypostomal tooth visible in whole or in part; in lateral view, petiole with sharp apical tooth (Fig. 18).

#### Distribution

This species is widely distributed in the Tropics of South America (Lattke 2011). In Colombia, it is known from the Departments of Cundinamarca and Meta (Fernández et al. 2019). It is reported here for the first time in the Department of Caquetá. In the Neotropical Region, the species has also been recorded in Bolivia, Brazil, Ecuador, Peru, Guyana, Suriname, French Guiana and Venezuela (Lattke 2011).

#### Biology

Specimens were collected from a log on the ground, using the TSBF sampling method in an Andean-Amazonian transition zone.

### Thaumatomyrmex atrox

Weber, 1939

CE11FB52-8654-5E21-A9F4-59A8B3CC019D

#### Materials

- **Type status:** Other material. **Occurrence:** catalogNumber: LEUA-00000066493; recordedBy: Brandon S. Arredondo; individualCount: 1; sex: female; occurrenceID: B9C3CFFA-47CB-5C66-BE57-8CE85C4D678E; **Taxon:** scientificName: Thaumatomyrmexatrox; kingdom: Animalia; phylum: Arthropoda; class: Insecta; order: Hymenoptera; family: Formicidae; genus: Thaumatomyrmex; specificEpithet: atrox; scientificNameAuthorship: Weber, 1939; **Location:** continent: South America; country: Colombia; countryCode: CO; stateProvince: Caquetá; county: Florencia; locality: Av.Caraño, Vía Florencia-Suaza; verbatimElevation: 919 m; locationRemarks: Collected in leaf Litter; verbatimCoordinates: 01°44'4.5"N 75°40'9.7"W; verbatimCoordinateSystem: WGS84; **Event:** samplingProtocol: Winkler; eventDate: 2023-09-30; **Record Level:** language: es; collectionID: RNC:270; institutionCode: Universidad de la Amazonia (UDLA); basisOfRecord: PreservedSpecimen

#### Diagnosis

**Figure 19.**
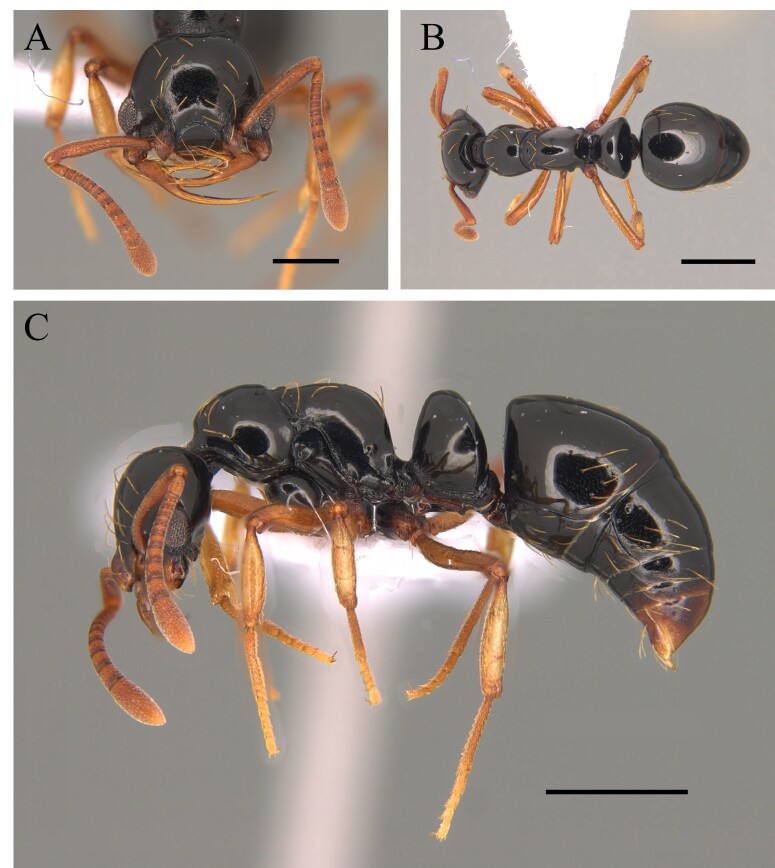
*Thaumatomyrmexatrox* worker (LEUA-00000066493) **A** head in frontal view; **B** body in dorsal view; **C** body in lateral view. Scale bars: 0.5 mm (A); 1.0 mm (B, C).

This species is recognised by the fact that, in frontal view, the apex of the apical tooth of the mandible clearly exceeds the lateral margin of the head and the external margin of the eye (Fig. 19).

#### Distribution

*Thaumatomyrmexatrox* is widely distributed in the Neotropical Region (Brandão 1991, Rodríguez and Lattke 2012, Albuquerque et al. 2021). In Colombia, *T.atrox* is widely distributed in the Andean and Caribbean Regions (Vergara-Navarro and Serna 2013), with a single record from the Department of Amazonas (Jahyny et al. 2008). It is reported here for the first time in the Department of Caquetá.

#### Biology

The specimen was collected in leaf litter from the lowest cloud forest in they Andean-Amazonian transition zone.

### Wadeura holmgrenita

Branstetter & Longino, 2022

2496C2A9-76FD-5993-800B-F6537C92929E

#### Materials

- **Type status:** Other material. **Occurrence:** catalogNumber: LEUA-00000061750; recordedBy: Brahyam Quimbaya; individualCount: 1; sex: female; occurrenceID: FA2782AD-E3D0-580A-86C4-038761961125; **Taxon:** scientificName: Wadeuraholmgrenita; kingdom: Animalia; phylum: Arthropoda; class: Insecta; order: Hymenoptera; family: Formicidae; genus: Wadeura; specificEpithet: holmgrenita; scientificNameAuthorship: Branstetter & Longino, 2022; **Location:** continent: South America; country: Colombia; countryCode: CO; stateProvince: Caquetá; county: Florencia; locality: Vda. Av. Caraño, Fca. El Chorro; verbatimElevation: 739 m; locationRemarks: From 10 cm to 20 cm deep in the ground; verbatimCoordinates: 01°44'27.2"N 75°39'12.3"W; verbatimCoordinateSystem: WGS84; **Event:** samplingProtocol: Monolite / TSBF; eventDate: 2022-12-08; **Record Level:** language: es; collectionID: RNC:270; institutionCode: Universidad de la Amazonia (UDLA); basisOfRecord: PreservedSpecimen
- **Type status:** Other material. **Occurrence:** catalogNumber: LEUA-00000061752; recordedBy: Brandon S. Arredondo; individualCount: 1; sex: female; occurrenceID: 5F6ECA96-0084-5FCC-8385-B7FE200C341D; **Taxon:** scientificName: Wadeuraholmgrenita; kingdom: Animalia; phylum: Arthropoda; class: Insecta; order: Hymenoptera; family: Formicidae; genus: Wadeura; specificEpithet: holmgrenita; scientificNameAuthorship: Branstetter & Longino, 2022; **Location:** continent: South America; country: Colombia; countryCode: CO; stateProvince: Caquetá; county: Florencia; locality: Vda. San Luis, Fca. El Triunfo; verbatimElevation: 700 m; locationRemarks: From 10 cm to 20 cm deep in the ground; verbatimCoordinates: 01°41'53.2"N 75°37'36.1"W; verbatimCoordinateSystem: WGS84; **Event:** samplingProtocol: Monolite / TSBF; eventDate: 2022-12-09; **Record Level:** language: es; collectionID: RNC:270; institutionCode: Universidad de la Amazonia (UDLA); basisOfRecord: PreservedSpecimen
- **Type status:** Other material. **Occurrence:** catalogNumber: LEUA-00000061755; recordedBy: Brandon S. Arredondo; individualCount: 1; sex: female; occurrenceID: F6C04C00-C85F-5742-9A87-C4E705174915; **Taxon:** scientificName: Wadeuraholmgrenita; kingdom: Animalia; phylum: Arthropoda; class: Insecta; order: Hymenoptera; family: Formicidae; genus: Wadeura; specificEpithet: holmgrenita; scientificNameAuthorship: Branstetter & Longino, 2022; **Location:** continent: South America; country: Colombia; countryCode: CO; stateProvince: Caquetá; county: Florencia; locality: Vda. San Luis, Fca. El Triunfo; verbatimElevation: 700 m; locationRemarks: From 10 cm to 20 cm deep in the groundo; verbatimCoordinates: 01°41'53.2"N 75°37'36.1"W; verbatimCoordinateSystem: WGS84; **Event:** samplingProtocol: Monolite / TSBF; eventDate: 2022-12-09; **Record Level:** language: es; collectionID: RNC:270; institutionCode: Universidad de la Amazonia (UDLA); basisOfRecord: PreservedSpecimen
- **Type status:** Other material. **Occurrence:** catalogNumber: LEUA-00000061756; recordedBy: Brandon S. Arredondo; individualCount: 1; sex: female; occurrenceID: 87966962-B7CA-5B4B-BE23-CD84039EC096; **Taxon:** scientificName: Wadeuraholmgrenita; kingdom: Animalia; phylum: Arthropoda; class: Insecta; order: Hymenoptera; family: Formicidae; genus: Wadeura; specificEpithet: holmgrenita; scientificNameAuthorship: Branstetter & Longino, 2022; **Location:** continent: South America; country: Colombia; countryCode: CO; stateProvince: Caquetá; county: Florencia; locality: Vda. La Paz, Fca. Patio Bonito; verbatimElevation: 663 m; locationRemarks: From 0 cm to 10 cm deep in the ground; verbatimCoordinates: 01°43'41.6"N 75°37'48.1"W; verbatimCoordinateSystem: WGS84; **Event:** samplingProtocol: Monolite / TSBF; eventDate: 2023-02-14; **Record Level:** language: es; collectionID: RNC:270; institutionCode: Universidad de la Amazonia (UDLA); basisOfRecord: PreservedSpecimen
- **Type status:** Other material. **Occurrence:** catalogNumber: LEUA-00000061757; recordedBy: Brandon S. Arredondo; individualCount: 1; sex: female; occurrenceID: D62E35AC-5135-55B2-9978-881BA9C8C65E; **Taxon:** scientificName: Wadeuraholmgrenita; kingdom: Animalia; phylum: Arthropoda; class: Insecta; order: Hymenoptera; family: Formicidae; genus: Wadeura ; specificEpithet: holmgrenita; scientificNameAuthorship: Branstetter & Longino, 2022; **Location:** continent: South America; country: Colombia; countryCode: CO; stateProvince: Caquetá; county: Florencia; locality: Vda. Sucre, Fca. San Antonio; verbatimElevation: 1409 m; locationRemarks: From 0 cm to 10 cm deep in the ground; verbatimCoordinates: 01°49'34.3"N 75°40'10.5"W; verbatimCoordinateSystem: WGS84; **Event:** samplingProtocol: Monolite / TSBF; eventDate: 2023-02-17; **Record Level:** language: es; collectionID: RNC:270; institutionCode: Universidad de la Amazonia (UDLA); basisOfRecord: PreservedSpecimen
- **Type status:** Other material. **Occurrence:** catalogNumber: LEUA-00000061772; recordedBy: Kenna Martinez; individualCount: 1; sex: female; occurrenceID: 9915C171-7AB1-57DE-A186-C5152A872822; **Taxon:** scientificName: Wadeuraholmgrenita; kingdom: Animalia; phylum: Arthropoda; class: Insecta; order: Hymenoptera; family: Formicidae; genus: Wadeura ; specificEpithet: holmgrenita; scientificNameAuthorship: Branstetter & Longino, 2022; **Location:** continent: South America; country: Colombia; countryCode: CO; stateProvince: Caquetá; county: Florencia; locality: Vda. Sucre, Fca. La Fortaleza; verbatimElevation: 1235 m; locationRemarks: From 10 cm to 20 cm deep in the ground; verbatimCoordinates: 01°48'18.5"N 75°39'14.0"W; verbatimCoordinateSystem: WGS84; **Event:** samplingProtocol: Monolite / TSBF; eventDate: 2023-02-10; **Record Level:** language: es; collectionID: RNC:270; institutionCode: Universidad de la Amazonia (UDLA); basisOfRecord: PreservedSpecimen
- **Type status:** Other material. **Occurrence:** catalogNumber: LEUA-00000061775; recordedBy: Brandon S. Arredondo; individualCount: 1; sex: female; occurrenceID: E224FABF-DF67-53D4-9748-3AD3A6ECEA86; **Taxon:** scientificName: Wadeuraholmgrenita; kingdom: Animalia; phylum: Arthropoda; class: Insecta; order: Hymenoptera; family: Formicidae; genus: Wadeura ; specificEpithet: holmgrenita; scientificNameAuthorship: Branstetter & Longino, 2022; **Location:** continent: South America; country: Colombia; countryCode: CO; stateProvince: Caquetá; county: Florencia; locality: Vda. La Paz, Fca. Patio Bonito; verbatimElevation: 663 m; locationRemarks: From 10 cm to 20 cm deep in the ground; verbatimCoordinates: 01°43'41.6"N 75°37'48.1"W; verbatimCoordinateSystem: WGS84; **Event:** samplingProtocol: Monolite / TSBF; eventDate: 2023-02-14; **Record Level:** language: es; collectionID: RNC:270; institutionCode: Universidad de la Amazonia (UDLA); basisOfRecord: PreservedSpecimen
- **Type status:** Other material. **Occurrence:** catalogNumber: LEUA-00000061776; recordedBy: Brandon S. Arredondo; individualCount: 1; sex: female; occurrenceID: D6CCBEB7-CCD5-5952-9BCB-D24878D25482; **Taxon:** scientificName: Wadeuraholmgrenita; kingdom: Animalia; phylum: Arthropoda; class: Insecta; order: Hymenoptera; family: Formicidae; genus: Wadeura ; specificEpithet: holmgrenita; scientificNameAuthorship: Branstetter & Longino, 2022; **Location:** continent: South America; country: Colombia; countryCode: CO; stateProvince: Caquetá; county: Florencia; locality: Vda. La Paz, Fca. Patio Bonito; verbatimElevation: 663 m; locationRemarks: From 10 cm to 20 cm deep in the ground; verbatimCoordinates: 01°43'41.6"N 75°37'48.1"W; verbatimCoordinateSystem: WGS84; **Event:** samplingProtocol: Monolite / TSBF; eventDate: 2023-02-14; **Record Level:** language: es; collectionID: RNC:270; institutionCode: Universidad de la Amazonia (UDLA); basisOfRecord: PreservedSpecimen
- **Type status:** Other material. **Occurrence:** catalogNumber: LEUA-00000061777; recordedBy: Brandon S. Arredondo; individualCount: 1; sex: female; occurrenceID: C7E81D31-708A-5BB5-A1B7-57BBD7F0D9BE; **Taxon:** scientificName: Wadeuraholmgrenita; kingdom: Animalia; phylum: Arthropoda; class: Insecta; order: Hymenoptera; family: Formicidae; genus: Wadeura ; specificEpithet: holmgrenita; scientificNameAuthorship: Branstetter & Longino, 2022; **Location:** continent: South America; country: Colombia; countryCode: CO; stateProvince: Caquetá; county: Florencia; locality: Vda. La Paz, Fca. Patio Bonito; verbatimElevation: 663 m; locationRemarks: From 10 cm to 20 cm deep in the ground; verbatimCoordinates: 01°43'41.6"N 75°37'48.1"W; verbatimCoordinateSystem: WGS84; **Event:** samplingProtocol: Monolite / TSBF; eventDate: 2023-02-14; **Record Level:** language: es; collectionID: RNC:270; institutionCode: Universidad de la Amazonia (UDLA); basisOfRecord: PreservedSpecimen
- **Type status:** Other material. **Occurrence:** catalogNumber: LEUA-00000061778; recordedBy: Brandon S. Arredondo; individualCount: 1; sex: female; occurrenceID: 614421D4-5925-5B15-BD18-6057E87971E5; **Taxon:** scientificName: Wadeuraholmgrenita; kingdom: Animalia; phylum: Arthropoda; class: Insecta; order: Hymenoptera; family: Formicidae; genus: Wadeura ; specificEpithet: holmgrenita; scientificNameAuthorship: Branstetter & Longino, 2022; **Location:** continent: South America; country: Colombia; countryCode: CO; stateProvince: Caquetá; county: Florencia; locality: Vda. La Paz, Fca. Patio Bonito; verbatimElevation: 663 m; locationRemarks: From 10 cm to 20 cm deep in the ground; verbatimCoordinates: 01°43'41.6"N 75°37'48.1"W; verbatimCoordinateSystem: WGS84; **Event:** samplingProtocol: Monolite / TSBF; eventDate: 2023-02-14; **Record Level:** language: es; collectionID: RNC:270; institutionCode: Universidad de la Amazonia (UDLA); basisOfRecord: PreservedSpecimen
- **Type status:** Other material. **Occurrence:** catalogNumber: LEUA-00000061779; recordedBy: Kenna Martinez; individualCount: 1; sex: female; occurrenceID: AC14A467-A128-50C4-B6D8-A23807D747E7; **Taxon:** scientificName: Wadeuraholmgrenita; kingdom: Animalia; phylum: Arthropoda; class: Insecta; order: Hymenoptera; family: Formicidae; genus: Wadeura ; specificEpithet: holmgrenita; scientificNameAuthorship: Branstetter & Longino, 2022; **Location:** continent: South America; country: Colombia; countryCode: CO; stateProvince: Caquetá; county: Florencia; locality: Vda. La Paz, Fca. Las Mercedes; verbatimElevation: 618 m; locationRemarks: From 0 cm to 10 cm deep in the ground; verbatimCoordinates: 01°43'39.2"N 75°38'00.7"W; verbatimCoordinateSystem: WGS84; **Event:** samplingProtocol: Monolite / TSBF; eventDate: 2023-02-15; **Record Level:** language: es; collectionID: RNC:270; institutionCode: Universidad de la Amazonia (UDLA); basisOfRecord: PreservedSpecimen
- **Type status:** Other material. **Occurrence:** catalogNumber: LEUA-00000061810; recordedBy: Brahyam Quimbaya; individualCount: 1; sex: female; occurrenceID: 9FBA8FF6-CCAA-535A-B0DC-81711012B1F0; **Taxon:** scientificName: Wadeuraholmgrenita; kingdom: Animalia; phylum: Arthropoda; class: Insecta; order: Hymenoptera; family: Formicidae; genus: Wadeura; specificEpithet: holmgrenita; scientificNameAuthorship: Branstetter & Longino, 2022; **Location:** continent: South America; country: Colombia; countryCode: CO; stateProvince: Caquetá; county: Florencia; locality: Vda. La Paz, Fca. Esperanza del Ruiseñor; verbatimElevation: 631 m; locationRemarks: From 20 cm to 30 cm deep in the ground; verbatimCoordinates: 01°43'47.4"N 75°37'40.0"W; verbatimCoordinateSystem: WGS84; **Event:** samplingProtocol: Monolite / TSBF; eventDate: 2023-02-13; **Record Level:** language: es; collectionID: RNC:270; institutionCode: Universidad de la Amazonia (UDLA); basisOfRecord: PreservedSpecimen
- **Type status:** Other material. **Occurrence:** catalogNumber: LEUA-00000061940; recordedBy: Brandon S. Arredondo; individualCount: 1; sex: female; occurrenceID: 4D1F3B9F-CCEC-5D03-B3FD-762881629DDC; **Taxon:** scientificName: Wadeuraholmgrenita; kingdom: Animalia; phylum: Arthropoda; class: Insecta; order: Hymenoptera; family: Formicidae; genus: Wadeura; specificEpithet: holmgrenita; scientificNameAuthorship: Branstetter & Longino, 2022; **Location:** continent: South America; country: Colombia; countryCode: CO; stateProvince: Caquetá; county: Florencia; locality: Vda. La Paz, Fca. Patio Bonito; verbatimElevation: 663 m; locationRemarks: From 0 cm to 10 cm deep in the ground; verbatimCoordinates: 01°43'41.6"N 75°37'48.1"W; verbatimCoordinateSystem: WGS84; **Event:** samplingProtocol: Monolite / TSBF; eventDate: 2023-02-14; **Record Level:** language: es; collectionID: RNC:270; institutionCode: Universidad de la Amazonia (UDLA); basisOfRecord: PreservedSpecimen
- **Type status:** Other material. **Occurrence:** catalogNumber: LEUA-00000061941; recordedBy: Brandon S. Arredondo; individualCount: 1; sex: female; occurrenceID: 7F559F62-7E9B-5442-A43E-A6CC039AF368; **Taxon:** scientificName: Wadeuraholmgrenita; kingdom: Animalia; phylum: Arthropoda; class: Insecta; order: Hymenoptera; family: Formicidae; genus: Wadeura; specificEpithet: holmgrenita; scientificNameAuthorship: Branstetter & Longino, 2022; **Location:** continent: South America; country: Colombia; countryCode: CO; stateProvince: Caquetá; county: Florencia; locality: Vda. La Paz, Fca. Patio Bonito; verbatimElevation: 663 m; locationRemarks: From 0 cm to 10 cm deep in the ground; verbatimCoordinates: 01°43'41.6"N 75°37'48.1"W; verbatimCoordinateSystem: WGS84; **Event:** samplingProtocol: Monolite / TSBF; eventDate: 2023-02-14; **Record Level:** language: es; collectionID: RNC:270; institutionCode: Universidad de la Amazonia (UDLA); basisOfRecord: PreservedSpecimen
- **Type status:** Other material. **Occurrence:** catalogNumber: LEUA-00000061942; recordedBy: Brandon S. Arredondo; individualCount: 1; sex: female; occurrenceID: F61708A7-8A66-515C-A92F-2DA35FF32174; **Taxon:** scientificName: Wadeuraholmgrenita; kingdom: Animalia; phylum: Arthropoda; class: Insecta; order: Hymenoptera; family: Formicidae; genus: Wadeura; specificEpithet: holmgrenita; scientificNameAuthorship: Branstetter & Longino, 2022; **Location:** continent: South America; country: Colombia; countryCode: CO; stateProvince: Caquetá; county: Florencia; locality: Vda. La Paz, Fca. Patio Bonito; verbatimElevation: 663 m; locationRemarks: From 0 cm to 10 cm deep in the ground; verbatimCoordinates: 01°43'41.6"N 75°37'48.1"W; verbatimCoordinateSystem: WGS84; **Event:** samplingProtocol: Monolite / TSBF; eventDate: 2023-02-14; **Record Level:** language: es; collectionID: RNC:270; institutionCode: Universidad de la Amazonia (UDLA); basisOfRecord: PreservedSpecimen
- **Type status:** Other material. **Occurrence:** catalogNumber: LEUA-00000061959; recordedBy: Brahyam Quimbaya; individualCount: 1; sex: female; occurrenceID: F677B4C3-F4F9-5F12-8397-C5D8F7E9848C; **Taxon:** scientificName: Wadeuraholmgrenita; kingdom: Animalia; phylum: Arthropoda; class: Insecta; order: Hymenoptera; family: Formicidae; genus: Wadeura; specificEpithet: holmgrenita; scientificNameAuthorship: Branstetter & Longino, 2022; **Location:** continent: South America; country: Colombia; countryCode: CO; stateProvince: Caquetá; county: Florencia; locality: Vda. Av. Caraño, Fca. La Esperanza; verbatimElevation: 604 m; locationRemarks: From 10 cm to 20 cm deep in the ground; verbatimCoordinates: 01°43'45.2"N 75°38'57.2"W; verbatimCoordinateSystem: WGS84; **Event:** samplingProtocol: Monolite / TSBF; eventDate: 2023-03-22; **Record Level:** language: es; collectionID: RNC:270; institutionCode: Universidad de la Amazonia (UDLA); basisOfRecord: PreservedSpecimen
- **Type status:** Other material. **Occurrence:** catalogNumber: LEUA-00000062154; recordedBy: Brandon S. Arredondo; individualCount: 1; sex: female; occurrenceID: 0153C4B6-A589-5BBE-9D4F-CD3BD35E9F18; **Taxon:** scientificName: Wadeuraholmgrenita; kingdom: Animalia; phylum: Arthropoda; class: Insecta; order: Hymenoptera; family: Formicidae; genus: Wadeura; specificEpithet: holmgrenita; scientificNameAuthorship: Branstetter & Longino, 2022; **Location:** continent: South America; country: Colombia; countryCode: CO; stateProvince: Caquetá; county: Florencia; locality: Vda. La Paz, Fca. Patio Bonito; verbatimElevation: 663 m; locationRemarks: From 20 cm to 30 cm deep in the ground; verbatimCoordinates: 01°43'41.6"N 75°37'48.1"W; verbatimCoordinateSystem: WGS84; **Event:** samplingProtocol: Monolite / TSBF; eventDate: 2023-02-14; **Record Level:** language: es; collectionID: RNC:270; institutionCode: Universidad de la Amazonia (UDLA); basisOfRecord: PreservedSpecimen
- **Type status:** Other material. **Occurrence:** catalogNumber: LEUA-00000062155; recordedBy: Brandon S. Arredondo; individualCount: 1; sex: female; occurrenceID: 9C80F28B-DE72-5F11-84F2-2B1092A98FA9; **Taxon:** scientificName: Wadeuraholmgrenita; kingdom: Animalia; phylum: Arthropoda; class: Insecta; order: Hymenoptera; family: Formicidae; genus: Wadeura; specificEpithet: holmgrenita; scientificNameAuthorship: Branstetter & Longino, 2022; **Location:** continent: South America; country: Colombia; countryCode: CO; stateProvince: Caquetá; county: Florencia; locality: Vda. La Paz, Fca. Patio Bonito; verbatimElevation: 663 m; locationRemarks: From 20 cm to 30 cm deep in the ground; verbatimCoordinates: 01°43'41.6"N 75°37'48.1"W; verbatimCoordinateSystem: WGS84; **Event:** samplingProtocol: Monolite / TSBF; eventDate: 2023-02-14; **Record Level:** language: es; collectionID: RNC:270; institutionCode: Universidad de la Amazonia (UDLA); basisOfRecord: PreservedSpecimen
- **Type status:** Other material. **Occurrence:** catalogNumber: LEUA-00000062156; recordedBy: Brandon S. Arredondo; individualCount: 1; sex: female; occurrenceID: 147B4F3E-B428-5585-9C90-3B680399ADD5; **Taxon:** scientificName: Wadeuraholmgrenita; kingdom: Animalia; phylum: Arthropoda; class: Insecta; order: Hymenoptera; family: Formicidae; genus: Wadeura; specificEpithet: holmgrenita; scientificNameAuthorship: Branstetter & Longino, 2022; **Location:** continent: South America; country: Colombia; countryCode: CO; stateProvince: Caquetá; county: Florencia; locality: Vda. La Paz, Fca. Patio Bonito; verbatimElevation: 663 m; locationRemarks: From 20 cm to 30 cm deep in the ground; verbatimCoordinates: 01°43'41.6"N 75°37'48.1"W; verbatimCoordinateSystem: WGS84; **Event:** samplingProtocol: Monolite / TSBF; eventDate: 2023-02-14; **Record Level:** language: es; collectionID: RNC:270; institutionCode: Universidad de la Amazonia (UDLA); basisOfRecord: PreservedSpecimen
- **Type status:** Other material. **Occurrence:** catalogNumber: LEUA-00000062472; recordedBy: Brandon S. Arredondo; individualCount: 1; sex: female; occurrenceID: 339AAFF3-F3D1-5987-8217-4E9FF729310D; **Taxon:** scientificName: Wadeuraholmgrenita; kingdom: Animalia; phylum: Arthropoda; class: Insecta; order: Hymenoptera; family: Formicidae; genus: Wadeura; specificEpithet: holmgrenita; scientificNameAuthorship: Branstetter & Longino, 2022; **Location:** continent: South America; country: Colombia; countryCode: CO; stateProvince: Caquetá; county: Florencia; locality: Vda. La Carbona, Fca. La Florida; verbatimElevation: 543 m; locationRemarks: From 0 cm to 10 cm deep in the ground; verbatimCoordinates: 01°42'12.5"N 75°38'05.4"W; verbatimCoordinateSystem: WGS84; **Event:** samplingProtocol: Monolite / TSBF; eventDate: 2023-05-17; **Record Level:** language: es; collectionID: RNC:270; institutionCode: Universidad de la Amazonia (UDLA); basisOfRecord: PreservedSpecimen
- **Type status:** Other material. **Occurrence:** catalogNumber: LEUA-00000062473; recordedBy: Brandon S. Arredondo; individualCount: 1; sex: female; occurrenceID: DE8A174E-3CCF-5391-A57B-F2FA85284C47; **Taxon:** scientificName: Wadeuraholmgrenita; kingdom: Animalia; phylum: Arthropoda; class: Insecta; order: Hymenoptera; family: Formicidae; genus: Wadeura; specificEpithet: holmgrenita; scientificNameAuthorship: Branstetter & Longino, 2022; **Location:** continent: South America; country: Colombia; countryCode: CO; stateProvince: Caquetá; county: Florencia; locality: Vda. La Carbona, Fca. La Florida; verbatimElevation: 543 m; locationRemarks: From 0 cm to 10 cm deep in the ground; verbatimCoordinates: 01°42'12.5"N 75°38'05.4"W; verbatimCoordinateSystem: WGS84; **Event:** samplingProtocol: Monolite / TSBF; eventDate: 2023-05-17; **Record Level:** language: es; collectionID: RNC:270; institutionCode: Universidad de la Amazonia (UDLA); basisOfRecord: PreservedSpecimen

#### Diagnosis

**Figure 20.**
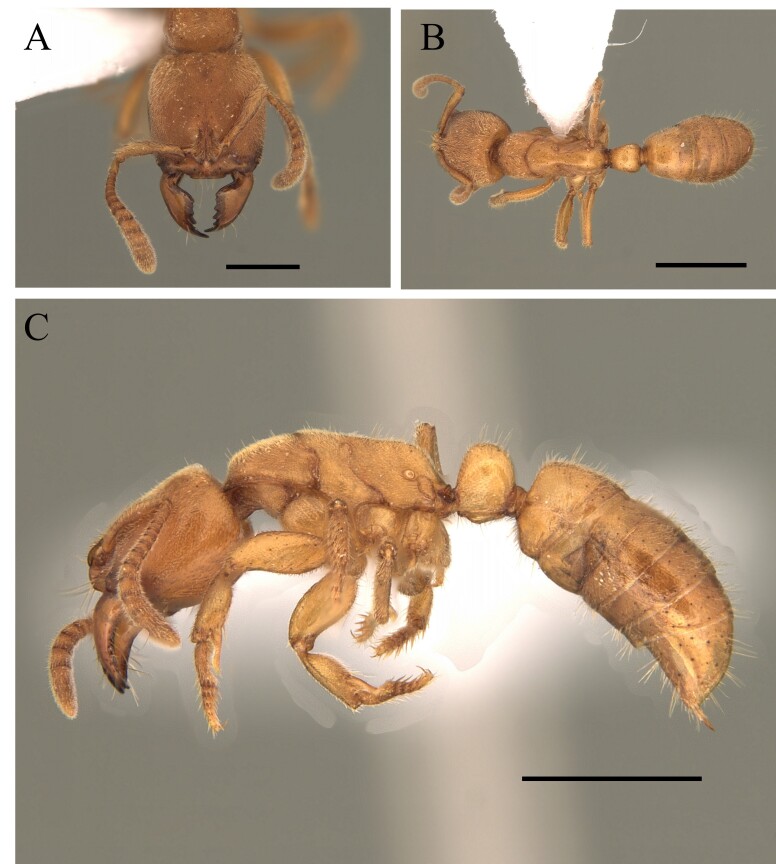
*Wadeuraholmgrenita* worker (LEUA-00000061779) **A** head in frontal view; **B** body in dorsal view; **C** body in lateral view. Scale bars: 0.5 mm (A); 1.0 (B, C).

This species is distinguished by a small triangular projection of the medial clypeus, not projecting beyond the outline of the clypeolabral junction; further, ventral margin of petiole is less symmetrical (Fig. 20).

#### Distribution

*Wadeuraholmgrenita* was previously known from Peru (Madre de Dios and Cusco) (Branstetter and Longino 2022). Although *W.holmgrenita* is considered a rarely collected species, our collection data suggest that it is widely distributed in the Basin of the Rio Hacha. This record represents the second known occurrence of the species in South America and the first record in Colombia.

#### Biology

The workers were collected 20 cm deep on the ground in the cloud forest of the Andean-Amazonian transition zone in Caquetá. Delabie et al. (2000) mentioned that *Wadeura* species could be specialised predators of soil termites.

#### Notes

The Colombian population of *W.holmgrenita* match largely with the type material of the species, at least in the shape of the subpeciolar process, mandibles and clypeus. The specimens from Caquetá present a slight variation in pilosity, having erect and suberect pilosity on the lateral margins of the head and generating an appearance of dense pilosity.

### Wadeura pauli

(Fernandes & Delabie, 2019)

0BF4B6C8-ABF6-574D-9114-FFBA489C427E

#### Materials

- **Type status:** Other material. **Occurrence:** catalogNumber: LEUA-00000061751; recordedBy: Brahyam Quimbaya; individualCount: 1; sex: female; occurrenceID: D22755A5-0B4F-516A-8D4B-5B5650603D66; **Taxon:** scientificName: Wadeurapauli; kingdom: Animalia; phylum: Arthropoda; class: Insecta; order: Hymenoptera; family: Formicidae; genus: Wadeura; specificEpithet: pauli; scientificNameAuthorship: (Fernandes & Delabie, 2019); **Location:** continent: South America; country: Colombia; countryCode: CO; stateProvince: Caquetá; county: Florencia; locality: Vda. Av. Caraño, Fca. El Chorro; verbatimElevation: 739 m; locationRemarks: From 0 cm to 10 cm deep in the ground; verbatimCoordinates: 01°44'28.9"N 75°39'12.8"W; verbatimCoordinateSystem: WGS84; **Event:** samplingProtocol: Monolite / TSBF; eventDate: 2022-12-08; **Record Level:** language: es; collectionID: RNC:270; institutionCode: Universidad de la Amazonia (UDLA); basisOfRecord: PreservedSpecimen
- **Type status:** Other material. **Occurrence:** catalogNumber: LEUA-00000061987; recordedBy: Brandon S. Arredondo; individualCount: 1; sex: female; occurrenceID: 72598922-4891-5DDD-ACB7-7787D7620C0A; **Taxon:** scientificName: Wadeurapauli; kingdom: Animalia; phylum: Arthropoda; class: Insecta; order: Hymenoptera; family: Formicidae; genus: Wadeura; specificEpithet: pauli; scientificNameAuthorship: (Fernandes & Delabie, 2019); **Location:** continent: South America; country: Colombia; countryCode: CO; stateProvince: Caquetá; county: Florencia; locality: Vda. Sucre, Fca. Carlos Endo; verbatimElevation: 998 m; locationRemarks: From 0 cm to 10 cm deep in the ground; verbatimCoordinates: 01°47'44.4"N 75°38'46.5"W; verbatimCoordinateSystem: WGS84; **Event:** samplingProtocol: Monolite / TSBF; eventDate: 2023-02-09; **Record Level:** language: es; collectionID: RNC:270; institutionCode: Universidad de la Amazonia (UDLA); basisOfRecord: PreservedSpecimen
- **Type status:** Other material. **Occurrence:** catalogNumber: LEUA-00000062000; recordedBy: Brandon S. Arredondo; individualCount: 1; sex: female; occurrenceID: 32D248E8-C736-5CE1-868B-9B75DAB5ABE2; **Taxon:** scientificName: Wadeurapauli; kingdom: Animalia; phylum: Arthropoda; class: Insecta; order: Hymenoptera; family: Formicidae; genus: Wadeura; specificEpithet: pauli; scientificNameAuthorship: (Fernandes & Delabie, 2019); **Location:** continent: South America; country: Colombia; countryCode: CO; stateProvince: Caquetá; county: Florencia; locality: Vda. San Francisco, Fca. La Esmeralda; verbatimElevation: 661 m; locationRemarks: From 10 cm to 20 cm deep in the ground; verbatimCoordinates: 01°43'00.6"N 75°35'37.7"W; verbatimCoordinateSystem: WGS84; **Event:** samplingProtocol: Monolite / TSBF; eventDate: 2023-04-19; **Record Level:** language: es; collectionID: RNC:270; institutionCode: Universidad de la Amazonia (UDLA); basisOfRecord: PreservedSpecimen

#### Diagnosis

**Figure 21.**
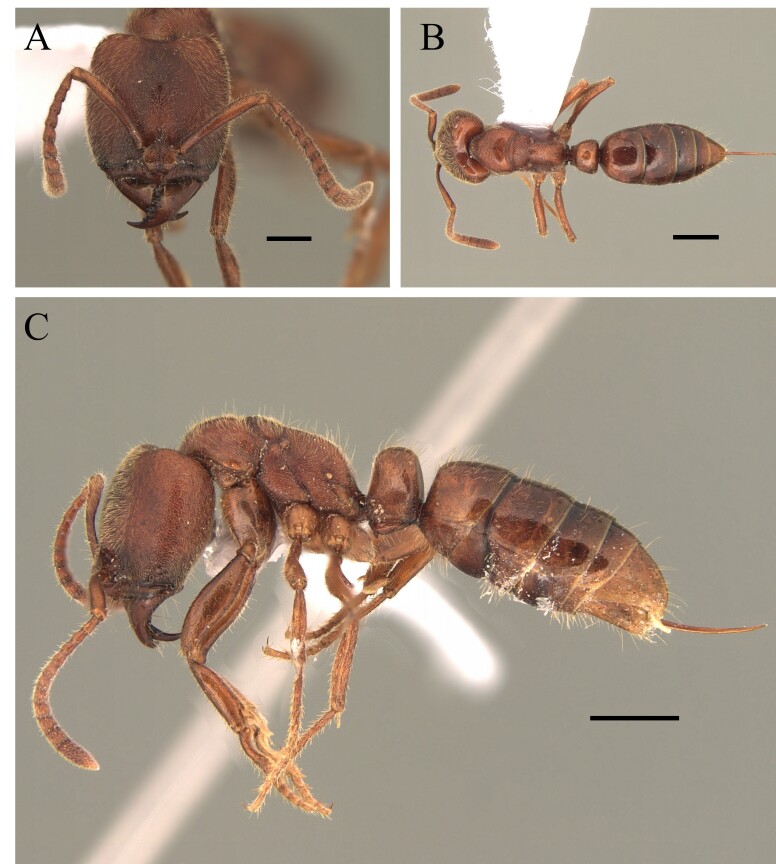
*Wadeurapauli* worker (LEUA-00000061987) **A** head in frontal view; **B** body in dorsal view; **C** body in lateral view. Scale bars: 0.5 mm (A); 1.0 (B, C).

This species is distinguished by the mandible triangular; ventral margin of petiole less symmetrical; ventral margin of petiole a triangular lobe with pronounced anterior tooth (Fig. 21).

#### Distribution

This is the first record of the species in Colombia, previously known from Guyana and Brazil, with records from only two localities, respectively (Branstetter and Longino 2022).

#### Biology

*W.pauli* was collected 20 cm deep on the ground, in the cloud forest of the Andean-Amazonian transition zone in Caquetá.

#### Notes

The population of *W.pauli* found in this study resemble morphologically the holotype, the shape of the subpeciolar process and dentition of the mandible matching the description of the species. Some specimens present a slight variation in pilosity, with areas of dense erect and suberect pilosity on the lateral margins of the head and dorsum of the mesosoma. In dorsal view, the petiole has a wider anterior face giving a trapezoidal shape to the petiole.

### Probolomyrmex kelleri

Oliveira & Feitosa, 2019

7A97666F-5D73-58B7-BA19-3B7EC953E631

#### Materials

- **Type status:** Other material. **Occurrence:** catalogNumber: LEUA-00000066494; recordedBy: Brandon S. Arredondo; individualCount: 1; sex: female; occurrenceID: B8724BBD-7DAB-50B8-A0C1-1540233729BD; **Taxon:** scientificName: Probolomyrmexkelleri; kingdom: Animalia; phylum: Arthropoda; class: Insecta; order: Hymenoptera; family: Formicidae; genus: Probolomyrmex ; specificEpithet: kelleri; scientificNameAuthorship: Oliveira & Feitosa, 2019; **Location:** continent: South America; country: Colombia; countryCode: CO; stateProvince: Caquetá; county: Florencia; locality: Vda. Las Doradas, Fca. Palmichal; verbatimElevation: 684 m; locationRemarks: Collected in leaf Litter; verbatimCoordinates: 01°43'01.3"N 75°40'24.2"W; verbatimCoordinateSystem: WGS84; **Event:** samplingProtocol: Winkler; eventDate: 2023-05-15; **Record Level:** language: es; collectionID: RNC:270; institutionCode: Universidad de la Amazonia (UDLA); basisOfRecord: PreservedSpecimen
- **Type status:** Other material. **Occurrence:** catalogNumber: LEUA-00000066495; recordedBy: Brandon S. Arredondo; individualCount: 1; sex: female; occurrenceID: A7C759B7-128B-5420-BD08-6A8D95ADEA42; **Taxon:** scientificName: Probolomyrmexkelleri; kingdom: Animalia; phylum: Arthropoda; class: Insecta; order: Hymenoptera; family: Formicidae; genus: Probolomyrmex ; specificEpithet: kelleri; scientificNameAuthorship: Oliveira & Feitosa, 2019; **Location:** continent: South America; country: Colombia; countryCode: CO; stateProvince: Caquetá; county: Florencia; locality: Vda. Las Doradas, Fca. Palmichal; verbatimElevation: 684 m; locationRemarks: Collected in leaf Litter; verbatimCoordinates: 01°43'01.3"N 75°40'24.2"W; verbatimCoordinateSystem: WGS84; **Event:** samplingProtocol: Winkler; eventDate: 2023-05-15; **Record Level:** language: es; collectionID: RNC:270; institutionCode: Universidad de la Amazonia (UDLA); basisOfRecord: PreservedSpecimen

#### Diagnosis

**Figure 22.**
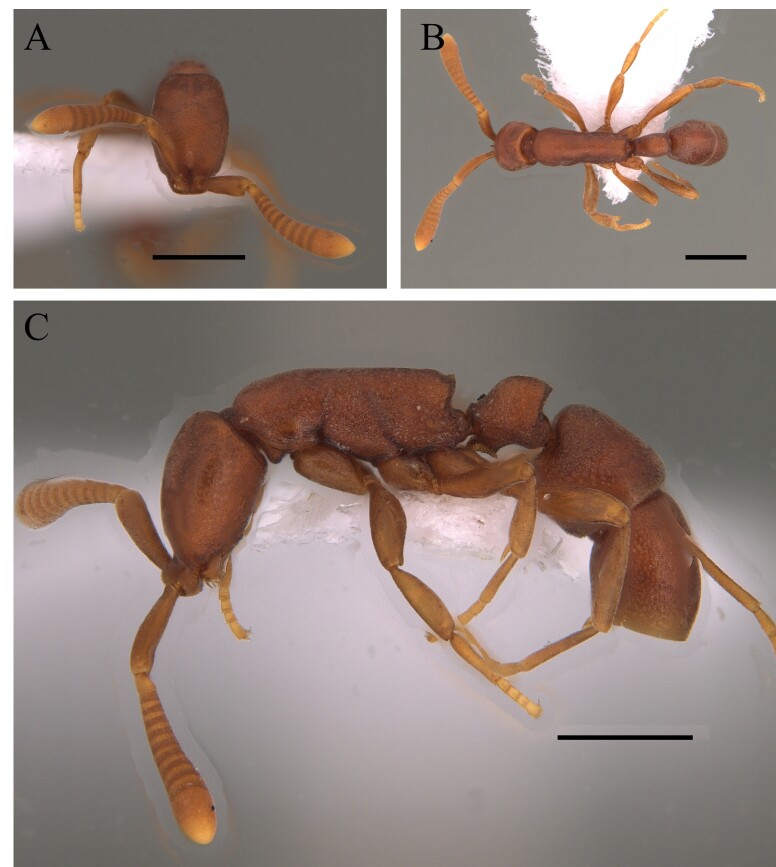
*Probolomyrmexkelleri* worker (LEUA-00000066494) **A** head in frontal view; **B** body in dorsal view; **C** body in lateral view. Scale bars: 0.5 mm (A, B, C).

This species is distinguished from the other *Probolomyrmex* species by the presence of subpetiolar process well developed, ventrally concave to sub-rectangular, with the postero-ventral angle acute and directed towards the gaster; prora and protuberance on the posterior region of the first gastral tergite present. (Fig. 22)

#### Distribution

This is the first record of the species for Colombia. Previously, this species had been recorded in Guyana, Peru and Venezuela. (Oliveira and Feitosa 2019).

#### Biology

The specimens were collected in leaf litter in the cloud forest in the Andean-Amazonian transition zone of Caquetá.

#### Notes

**Figure 23.**
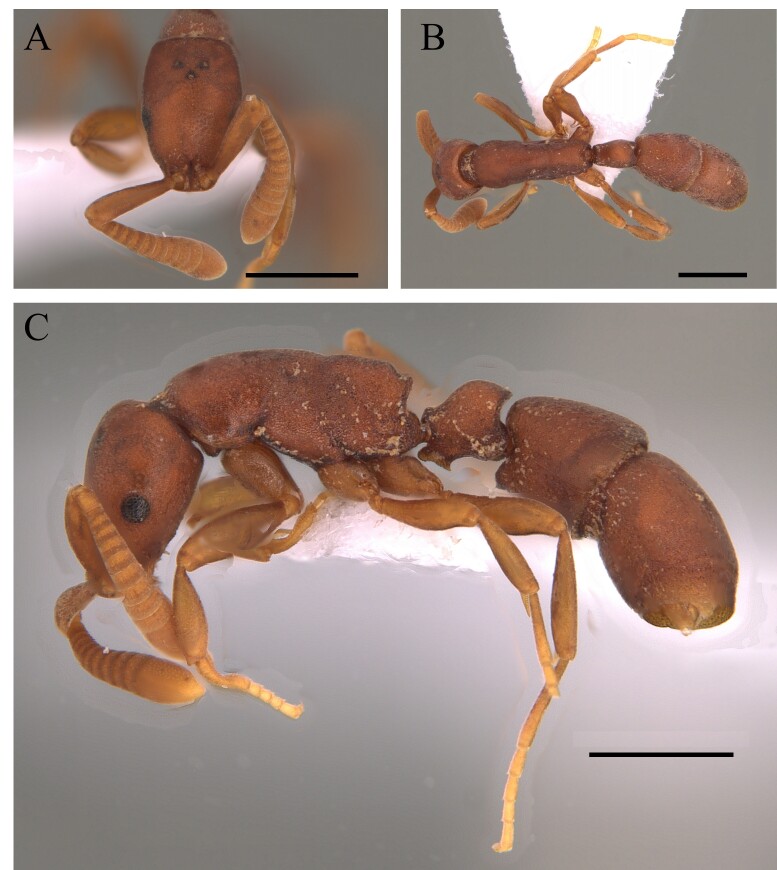
*Probolomyrmexkelleri* ergatoid (LEUA-00000066495) **A** head in frontal view; **B** body in dorsal view; **C** body in lateral view. Scale bars: 0.5 mm (A, B, C).

We document here the first case for the species of an ergatoid queen (Fig. 23). The ergatoid queen has developed compound eyes and ocelli; further, the mesosoma is a little wider and higher than workers. The shape of the subpetiolar process in the ergatoid queen and the worker is identical, this feature being important to separate *P.kelleri* from any other species of this genus.

## Checklists

### Checklist of ant species of Caquetá Department (Colombia)

#### Fulakora orizabana

(Brown, 1960)

DDE5CAC0-35F0-5956-B53C-7F56C3836B74

##### Distribution

Caquetá, Caldas, Cauca, Chocó, Quindío, Risaralda, Valle del Cauca (Fernández et al. 2019).

#### Prionopelta amabilis

Borgmeier, 1949

CA17EA42-A20F-5D6B-B91E-3C96E437D357

##### Distribution

Amazonas, Antioquia, Caquetá, Cauca, Meta, Nariño, Quindío, Santander, Valle del Cauca, Vaupés (Fernández et al. 2019, Ladino and Feitosa 2020).

#### Prionopelta antillana

Forel, 1909

4F95AE12-EB2F-5AFD-B7A6-73E17BE82F6D

##### Distribution

Amazonas, Caquetá, Caldas, Cauca, Magdalena, Nariño, Putumayo, Valle del Cauca (Chacón de Ulloa et al. 2012, Fernández et al. 2019).

#### Dolichoderus abruptus

(Smith, 1858)

DF0F2EEA-8B7B-5D25-9B40-2259F2DE06E6

##### Distribution

Amazonas, Antioquia, Caquetá, Guaviare, Meta (Fernández et al. 1996, Fernández et al. 2019).

#### Dolichoderus attelaboides

(Fabricius, 1775)

E82E68F0-52C7-5C4A-A1ED-356499FB6E82

##### Distribution

Amazonas, Antioquia, Caquetá, Guaviare, Meta, Putumayo, Valle del Cauca, Vaupés, Vichada (Fernández et al. 1996, Fernández et al. 2019).

#### Dolichoderus bidens

(Linnaeus, 1758)

E598165E-E4A3-58A0-8679-EDE0CC142183

##### Distribution

Amazonas, Boyacá, Caquetá, Chocó, Guaviare, Magdalena, Meta, Valle del Cauca (Fernández et al. 1996, Ortíz and Fernández 2011, Fernández et al. 2019).

#### Dolichoderus bispinosus

(Olivier, 1792)

1A839261-1BEF-5846-AB61-EFFB4D84C628

##### Distribution

Amazonas, Antioquia, Bolivar, Caldas, Caquetá, Chocó, Cundinamarca, Guaviare, La Guajira, Magdalena, Meta, Putumayo, Risaralda, Santander, Sucre, Valle del Cauca, Vichada (Fernández et al. 1996, Fernandez and Sendoya 2004, Ortíz and Fernández 2011, Fernández et al. 2019).

#### Dolichoderus cogitans

Forel, 1912

7448EA9D-55B8-574A-A8EC-FC63C98F13E8

##### Distribution

Amazonas, Antioquia, Boyacá, Caquetá, Meta (Fernández et al. 1996, Fernández et al. 2019).

#### Dolichoderus curvilobus

(Lattke, 1987)

B0D9657E-62E5-5DFA-B930-AFB2BA6DC416

##### Distribution

Amazonas, Antioquia, Boyacá, Caquetá, Chocó, Nariño, Santander, Valle del Cauca (Fernández et al. 1996, Ortíz and Fernández 2011, Fernández et al. 2019).

#### Dolichoderus debilis

Emery, 1890

48FF332A-20CF-5EC8-8785-3E18032A90BA

##### Distribution

Amazonas, Antioquia, Bolivar, Caquetá, Cauca, La Guajira, Magdalena, Meta, Putumayo (Ortíz and Fernández 2011, Fernández et al. 2019).

##### Notes

New record from Caquetá

#### Dolichoderus decollatus

Smith, 1858

CC84E5DC-7572-590D-8C44-B7E93FDB0473

##### Distribution

Amazonas, Antioquia, Caldas, Caquetá, Chocó, Cundinamarca, La Guajira, Magdalena, Meta, Putumayo, Risaralda, Vichada (Fernández et al. 2019).

#### Dolichoderus ferrugineus

Forel, 1903

A0EAF474-9C81-59AD-B2BA-BC44D1F6C5B5

##### Distribution

Amazonas, Caquetá, Putumayo, Vaupés (Fernández et al. 1996, Ortíz and Fernández 2011, Fernández et al. 2019).

#### Dolichoderus ghilianii

Emery, 1894

630D3308-0160-5205-994E-BC24D718A914

##### Distribution

Amazonas, Antioquia, Caquetá, Chocó (Fernández et al. 1996, Fernández et al. 2019).

#### Dolichoderus imitator

Emery, 1894

6716D611-320C-568A-95C2-B0F3A21D3012

##### Distribution

Amazonas, Antioquia, Caldas, Caquetá, Cundinamarca, Guainía, Guaviare, Magdalena, Meta, Putumayo, Vaupés, Valle del Cauca (Ortíz and Fernández 2011, Castro et al. 2018, Fernández et al. 2019).

#### Dolichoderus inpai

(Harada, 1987)

5F8E1313-F377-5452-917A-14777ADC1C37

##### Distribution

Amazonas, Caquetá, Meta (Fernández et al. 1996, Fernández et al. 2019).

#### Dolichoderus lamellosus

(Mayr, 1870)

238BAEA7-FEFD-5A9E-A6A8-946A9061BB3D

##### Distribution

Amazonas, Caquetá, Chocó, Magdalena, Meta, Putumayo (Fernández et al. 1996, Fernández et al. 2019).

#### Dolichoderus lutosus

(Smith, 1858)

F18D4DB0-1756-5909-A282-833218D0A15E

##### Distribution

Amazonas, Antioquia, Bolívar, Caquetá, Cauca, Cundinamarca, La Guajira, Magdalena, Meta, Valle del Cauca, Vaupés, Vichada (Chacón de Ulloa and Abadía 2014, Fernández et al. 2019).

#### Dolichoderus mucronifer

(Roger, 1862)

39613000-3E63-5C71-8CCC-143BADF9DC38

##### Distribution

Amazonas, Caquetá (Fernández et al. 1996, Fernández et al. 2019).

#### Dolichoderus quadridenticulatus

(Roger, 1862)

F179032D-55D4-541D-95C5-49AFEA5DD20B

##### Distribution

Amazonas, Antioquia, Caquetá, Chocó, Meta, Santander, Valle del Cauca (Fernández et al. 1996, Fernández et al. 2019).

#### Dolichoderus rugosus

(Smith, 1858)

53A09039-D233-55C0-AD70-47644AF32774

##### Distribution

Amazonas, Antioquia, Caquetá, Putumayo (Fernández et al. 2019).

##### Notes

New record from Caquetá

#### Dolichoderus schulzi

Emery, 1894

B3D909FA-6EDC-5A83-9B34-F64F2E29D998

##### Distribution

Amazonas, Antioquia, Caquetá (Fernández et al. 1996, Fernández et al. 2019).

#### Dorymyrmex brunneus

Forel, 1908

2C765E54-D6D4-5537-B1FD-D7E8776789EB

##### Distribution

Amazonas, Antioquia, Boyacá, Caldas, Caquetá, Cundinamarca, Nariño, Meta, Quindío, Santander, Tolima, Valle del Cauca, Vichada (Fernández et al. 2019).

#### Gracilidris pombero

Wild & Cuezzo, 2006

F8CAB43D-2A6E-55F3-B157-F65E4F3D1064

##### Distribution

Caquetá (Fernández et al. 2019).

#### Linepithema angulatum

(Emery, 1894)

28330CE4-EBF1-553D-B8CA-D8BFCFA8283A

##### Distribution

Boyacá, Caldas, Caquetá, Cauca, Cundinamarca, Huila, Magdalena, Meta, Nariño, Putumayo, Quindío, Risaralda, Santander, Tolima, Vichada, Valle del Cauca (Fernández et al. 2019).

#### Linepithema neotropicum

Wild, 2007

DE1C91E3-0D8B-5BBE-ABEF-98595FE00621

##### Distribution

Antioquia, Boyacá, Caldas, Caquetá, Cauca, Cundinamarca, La Guajira, Huila, Magdalena, Meta, Tolima, Nariño, Quindío, Risaralda, Valle del Cauca, Vichada (Escárraga and Guerrero 2016, Fernández et al. 2019).

#### Linepithema piliferum

(Mayr, 1870)

3991E80A-9D68-57FA-AD6E-A3912447E7A3

##### Distribution

Antioquia, Boyacá, Caldas, Caquetá, Cauca, Cundinamarca, Chocó, Huila, La Guajira, Nariño, Norte de Santandertander, Putumayo, Quindío, Risaralda, Tolima, Valle del Cauca (Fernández et al. 2019).

#### Tapinoma melanocephalum

(Fabricius, 1793)

DCF25BA1-06E8-5AFE-B522-C55ADDC25ADB

##### Distribution

Present throughout the country (Fernández et al. 2019).

#### Cheliomyrmex andicola

Emery, 1894

8164EE44-1214-59FC-9B62-5EC55D4359FD

##### Distribution

Amazonas, Antioquia, Caquetá, Cauca, Cundinamarca, Meta, Nariño, Risaralda, Tolima, Valle del Cauca (Sandoval and Zambrano 2007, Sanabria-Blandón and Achury 2011, Fernández et al. 2019).

#### Eciton burchellii

(Westwood, 1842)

E29AC699-8272-5EA3-85FD-CDA45EE4376A

##### Distribution

Amazonas, Antioquia, Bolivar, Caldas, Caquetá, Cauca, Casanare, Chocó, Cundinamarca, La Guajira, Guaviare, Huila, Magdalena, Meta, Nariño, Putumayo, Risaralda, Santander, Tolima, Vichada (Fernández et al. 1996, Fernández et al. 2019).

#### Eciton dulcium

Forel, 1912

92D84F99-2AC5-549B-BDF0-D6CE899E00E8

##### Distribution

Amazonas, Caquetá, Huila, Valle del Cauca (Sanabria-Blandón and Achury 2011, Fernández et al. 2019).

#### Eciton hamatum

(Fabricius, 1782)

9563ED0C-C758-5BCD-ABE8-D5DA150BDFD8

##### Distribution

Antioquia, Caquetá, Chocó, Cundinamarca, Guaviare, Huila, La Guajira, Magdalena, Meta, Nariño, Risaralda, Tolima, Valle del Cauca (Fernández et al. 1996, Fernández et al. 2019).

#### Eciton rapax

Smith, 1855

80EF22EA-8EA3-5D57-A82E-B6CAE67C64EA

##### Distribution

Amazonas, Caquetá, Guaviare, Meta, Putumayo (Fernández et al. 1996, Fernández et al. 2019).

#### Eciton vagans

(Olivier, 1792)

8A2B82EE-4317-570A-AE1B-B7217DAAE8F9

##### Distribution

Amazonas, Caldas, Caquetá, Cauca, Casanare, Chocó, Cundinamarca, Guaviare, Huila, Magdalena, Meta, Nariño, Valle del Cauca (Fernández et al. 1996, Fernández et al. 2019).

#### Labidus coecus

(Latreille, 1802)

2B87227A-2B99-5FE2-AA02-D13CDCF16BCD

##### Distribution

Amazonas, Antioquia, Bolívar, Boyacá, Caldas, Caquetá, Casanare, Chocó, Cundinamarca, Guaviare, Magdalena, Meta, Nariño, Quindío, Risaralda, Santander, Tolima, Valle del Cauca (Sanabria-Blandón and Achury 2011, Fernández et al. 2019).

#### Labidus praedator

(Smith, 1858)

1AFED619-7651-5431-8CB7-D24CA2F6C369

##### Distribution

Amazonas, Antioquia, Arauca, Bolívar, Boyacá, Caquetá, Cauca, Casanare, Chocó, Cundinamarca, Guaviare, Huila, Magdalena, Nariño, Quindío, Risaralda, Tolima, Valle del Cauca (Castro et al. 2018, Fernández et al. 2019).

#### Neivamyrmex clavifemur

Borgmeier, 1953

57617876-F31D-54F6-8195-6BB761C147F3

##### Distribution

Caquetá (Fernández et al. 2019).

#### Neivamyrmex punctaticeps

(Emery, 1894)

050EF97A-D782-5679-90A2-9A47F353F91A

##### Distribution

Amazonas, Boyacá, Caquetá, Cauca, Cundinamarca, Nariño, Santander, Tolima (Gracía-Cardenas et al. 2018, Fernández et al. 2019).

#### Nomamyrmex esenbeckii

(Westwood, 1842)

F581E3E9-91BA-59AB-874F-98E194F34B9A

##### Distribution

Amazonas, Antioquia, Arauca, Bolívar, Caldas, Caquetá, Chocó, Cundinamarca, Magdalena, Meta, Nariño, Santander, Tolima, Valle del Cauca (Fernández et al. 1996, Fernández et al. 2019).

#### Syscia austrella

Longino & Branstetter, 2021

8811F8FA-A540-52D3-B3F6-72035E709E6D

##### Distribution

Caquetá, Vaupés (Longino and Branstetter 2021).

#### Syscia minuta

Longino & Branstetter, 2021

B6DA848E-0352-5ADA-ABD0-08E14F1EF6E3

##### Distribution

Caquetá (Longino and Branstetter 2021).

#### Acanthoponera minor

(Forel, 1899)

A66F0BF2-5D8B-5C84-A40D-C2F336BA94EC

##### Distribution

Antioquia, Caquetá, Chocó, Magdalena, Norte de Santander, Risaralda, Valle del Cauca (Fernández et al. 2019).

#### Bazboltonia microps

(Borgmeier, 1957)

C0598FF7-6370-504E-B775-0DC5BA5CDEBC

##### Distribution

Amazonas, Antioquia, Caldas, Caquetá, Cauca, Huila, Quindío, Risaralda, Santander, Tolima, Valle del Cauca (Fernández et al. 2019).

#### Ectatomma brunneum

Smith, 1858

E9011AA7-3A0D-515E-8764-E7AD31FE1041

##### Distribution

Amazonas, Antioquia, Boyacá, Caquetá, Cundinamarca, Chocó, Guaviare, Meta, Quindío, Sucre, Valle del Cauca, Vichada (Fernández et al. 2019).

#### Ectatomma edentatum

Roger, 1863

C1B1B720-86C5-53A5-9B8C-8861ABA6E969

##### Distribution

Amazonas, Caquetá, Meta, Valle del Cauca (Fernández et al. 2019).

#### Ectatomma goninion

Kugler & Brown, 1982

C7726D95-EBDE-5B4D-B982-1A96A40F0F9E

##### Distribution

Caquetá, Chocó, Cauca, Nariño, Risaralda (Lozano-Zambrano et al. 2008, Fernández et al. 2019).

#### Ectatomma lugens

Emery, 1894

FA3DDF64-0991-5FDC-A089-EB50563E2069

##### Distribution

Amazonas, Caquetá, Cauca, Meta, Putumayo, Valle del Cauca (Kugler 1991, Lozano-Zambrano et al. 2008, Fernández et al. 2019).

#### Ectatomma ruidum

(Roger, 1860)

5ABCF9D9-0DDC-50A8-B30C-35A61AD00587

##### Distribution

Amazonas, Antioquia, Atlántico, Bolívar, Boyacá, Caquetá, Córdoba, Cundinamarca, Chocó, Huila, Magdalena, Meta, Santander, Sucre, Tolima, Valle del Cauca, Vichada (Fernández et al. 2019).

#### Ectatomma tuberculatum

(Olivier, 1792)

7B3D8E60-FF28-587A-9BAA-1FDE3C8A010E

##### Distribution

Amazonas, Antioquia, Caquetá, Casanare, Cauca, Cundinamarca, Chocó, Guaviare, Magdalena, Meta, Nariño, Risaralda, Santander, Valle del Cauca, Vichada (Castro et al. 2018, Fernández et al. 2019).

#### Gnamptogenys acuminata

(Emery, 1896)

1CFF7088-3350-51B3-B3A5-4C25422B86D7

##### Distribution

Caquetá, Meta, Caquetá, Nariño, Putumayo, Valle del Cauca (Fernández et al. 2019).

#### Gnamptogenys annulata

(Mayr, 1887)

2FA5F660-C870-5AC9-A68F-739B54DCF178

##### Distribution

Amazonas, Antioquia, Caquetá, Cauca, Cundinamarca, Meta, Nariño, Norte de Santander, Quindío, Valle del Cauca (Fernández et al. 2019).

#### Gnamptogenys concinna

(Smith, 1858)

89D17CB0-87CC-5A31-9EEF-67AB9175F9E0

##### Distribution

Amazonas, Caquetá, Meta, Nariño, Valle del Cauca (Fernández et al. 2019).

#### Gnamptogenys continua

(Mayr, 1887)

9E5BEE77-E19E-5A88-904A-A3F434995097

##### Distribution

Amazonas, Antioquia, Caldas, Caquetá, Cauca, Cundinamarca, Magdalena, Nariño, Valle del Cauca (Fernández et al. 2019).

#### Gnamptogenys ericae

(Forel, 1912)

C0C1160E-5CE2-5E19-9D8F-9A254D88CFA9

##### Distribution

Amazonas, Atlántico, Bolívar, Caquetá, Casanare, La Guajira, Magdalena, Meta, Putumayo, Sucre, Vichada (Fernández et al. 2019).

#### Gnamptogenys fernandezi

Lattke, 1990

5009E601-8A99-5696-BDD7-C8E4253AF1FC

##### Distribution

Caquetá, Cauca, Valle del Cauca, Vaupés (Fernández et al. 2019).

#### Gnamptogenys haenschi

(Emery, 1902)

DAEDFA5E-4568-593F-9C4B-DFDC73200585

##### Distribution

Amazonas, Antioquia, Caquetá, Cauca, Chocó, La Guajira, Magdalena, Meta, Risaralda, Valle del Cauca (Fernández et al. 2019).

#### Gnamptogenys hartmani

(Wheeler, 1915)

D009C6AE-DD9A-5C61-B356-D9F3F8427E5C

##### Distribution

Antioquia, Caquetá (Fernández et al. 2019).

#### Gnamptogenys horni

(Santschi, 1929)

A1CDFAF4-3656-55E9-9F97-63EA07570CA0

##### Distribution

Antioquia, Caquetá, Cauca, Chocó, Guaviare, Meta, Nariño, Putumayo, Risaralda, Valle del Cauca, Vaupés (Fernández et al. 2019).

#### Gnamptogenys kempfi

Lenko, 1964

90044296-65EB-5449-A49C-D57271510946

##### Distribution

Amazonas, Caquetá (Fernández et al. 2019).

#### Gnamptogenys mordax

(Smith F, 1858)

6E3B558F-B54A-51F3-8EA9-72A12AF981E7

##### Distribution

Amazonas, Antioquia, Caquetá, Chocó, Cundinamarca, Norte de Santander, Risaralda, Santander, Valle del Cauca (Fernández et al. 2019).

#### Gnamptogenys regularis

Mayr, 1870

8B4D88FA-AEE4-5881-888F-8D4F8D26E01B

##### Distribution

Amazonas, Bolívar, Boyacá, Caquetá, Cauca, Magdalena, Meta, Putumayo, Valle del Cauca (Fernández et al. 2019).

#### Gnamptogenys sulcata

(Smith F, 1858)

7EE9ABAD-4D71-590B-93B6-A0A923BDD237

##### Distribution

Amazonas, Antioquia, Atlántico, Caquetá, Casanare, Cauca, Chocó, Magdalena, Meta, Nariño, Putumayo, Sucre, Valle del Cauca, Vaupés (Fernández et al. 2019).

#### Gnamptogenys tortuolosa

(Smith F, 1858)

3150EDAD-5460-55DF-ACCE-41282A6E77EA

##### Distribution

Amazonas, Caquetá, Cundinamarca, Chocó, Guaviare, Meta, Putumayo (Lattke et al. 2004, Fernández et al. 2019).

#### Heteroponera monticola

Kempf & Brown, 1970

D80CE08B-D151-59C8-B9F2-523D7A13D8A3

##### Distribution

Antioquia, Caldas, Caquetá, Cundinamarca, Huila, Meta, Nariño, Quindío, Risaralda, Valle del Cauca (Fernández et al. 2019).

#### Holcoponera andina

(Lattke, 1995)

880BE332-02EB-5EB2-A606-4D007C8DB76D

##### Distribution

Amazonas, Antioquia, Caldas, Caquetá, Nariño, Norte de Santander, Quindío, Risaralda, Valle del Cauca, Vichada (Fernández et al. 2019).

#### Holcoponera mina

Brown, 1956

BC52712F-F404-58DC-B0CD-9B125616B1A4

##### Distribution

Amazonas, Caquetá, Nariño, Putumayo, Valle del Cauca (Calle et al. 2013, Fernández et al. 2019).

#### Holcoponera moelleri

Forel, 1912

DE614D0B-DA0A-5713-A1A7-73031EBCC55F

##### Distribution

Amazonas, Boyacá, Caquetá, Cauca, Chocó, Cundinamarca, Meta, Nariño, Norte de Santander, Putumayo, Vaupés (Fernández et al. 2019).

#### Holcoponera nigrivitrea

(Lattke, 1995)

79E0ADF2-3B0D-5A3E-B05E-5650272DE603

##### Distribution

Caldas, Caquetá, Cundinamarca, Huila, Nariño, Putumayo, Quindío, Risaralda, Santander, Valle del Cauca (Fernández et al. 2019).

#### Holcoponera pleurodon

(Emery, 1896)

1A2D1427-2A76-59BD-AC37-8CA8CB0B5109

##### Distribution

Amazonas, Caquetá, Guaviare, Magdalena, Nariño, Putumayo, Valle del Cauca (Fernández et al. 2019).

#### Holcoponera porcata

(Emery, 1896)

52E424A1-9DD6-5BB1-9B0D-DA98D7428263

##### Distribution

Antioquia, Caquetá, Chocó, Cundinamarca, La Guajira, Guaviare, Huila, Nariño, Norte de Santander, Santander, Valle del Cauca (Fernández et al. 2019).

#### Holcoponera striatula

(Mayr, 1884)

FD6A0851-7267-5295-81A8-804E886D61AB

##### Distribution

Amazonas, Antioquia, Atlántico, Bolívar, Caldas, Caquetá, Chocó, Cundinamarca, Guaviare, Meta, Nariño, Norte de Santander, Quindío, Risaralda, Valle del Cauca, Vaupés (Fernández et al. 2019).

#### Holcoponera strigata

(Norton, 1871)

FB62A79C-3FC2-5E84-96DE-35890EDE1D39

##### Distribution

Antioquia, Caldas, Caquetá, Cundinamarca, Huila, Nariño, Norte de Santander, Putumayo, Quindío, Risaralda, Valle del Cauca, Vichada (Fernández et al. 2019).

#### Typhlomyrmex clavicornis

Emery, 1906

EF0EAD32-7A98-5589-9646-9ABCB51340E9

##### Distribution

Caquetá, Putumayo (Castro et al. 2018, Fernández et al. 2019).

#### Typhlomyrmex major

Santschi, 1923

3C914348-2823-59BE-8311-BE52BC93E6EC

##### Distribution

Amazonas, Caquetá, Nariño (Castro et al. 2018, Fernández et al. 2019).

#### Typhlomyrmex meire

Lacau et al., 2004

08FCA402-982C-5BE7-A16D-CF4BFD230012

##### Distribution

Caquetá (Castro et al. 2018).

#### Typhlomyrmex prolatus

Brown, 1965

A730F975-9C9D-5F5D-9834-54835CDF3A94

##### Distribution

Caquetá (Fernández et al. 2023).

#### Typhlomyrmex pusillus

Emery, 1894

34166C56-4F8D-55B2-BCC9-E3B40245E4C1

##### Distribution

Antioquia, Caldas, Caquetá, Cauca, Nariño, Quindío, Risaralda, Valle del Cauca (Fernández et al. 2019).

#### Typhlomyrmex rogenhoferi

Mayr, 1862

0274AFDB-DDF0-5DCE-8BA0-3E2F3515E38D

##### Distribution

Amazonas, Caldas, Caquetá, Cauca, Cundinamarca, Huila, Meta, Valle del Cauca (Fernández et al. 2019).

#### Acropyga exsanguis

(Wheeler, 1909)

F276C46E-BD75-5881-9213-8A8A367C1785

##### Distribution

Antioquia, Caquetá, Cundinamarca, Risaralda, Valle del Cauca (Chacón de Ulloa et al. 2012, LaPolla 2004, Castro et al. 2018, Fernández et al. 2019).

#### Acropyga goeldii

Forel, 1893

91B77503-3823-5A6A-9586-4C5D169B1268

##### Distribution

Amazonas, Antioquia, Caquetá, Risaralda, Valle del Cauca (Fernández et al. 1996, Vergara-Navarro and Serna 2013, Castro et al. 2018, Fernández et al. 2019).

#### Brachymyrmex australis

Forel, 1901

3356C961-93DB-5E2A-9060-708AD60C90A0

##### Distribution

Bolívar, Caquetá, Cauca, Caldas, Cundinamarca, Huila, La Guajira, Meta, Quindío, Risaralda, Valle del Cauca, Vichada (Fernández et al. 2019, Ortiz-Sepuvelda et al. 2019).

#### Brachymyrmex cordemoyi

Forel, 1895

48F04C00-F488-5CFD-A82E-56D92FBB2C23

##### Distribution

Caquetá, Huila, Meta (Fernández et al. 2019).

#### Brachymyrmex pictus

Mayr, 1887

433424FA-0E3D-5EB8-85AD-7F63964959DE

##### Distribution

Caquetá, Cauca, Magdalena, Putumayo, Valle del Cauca (Calle et al. 2013, Fernández et al. 2019).

#### Camponotus amoris

Forel, 1904

17D2054A-C89C-516E-8392-736E4F6B6390

##### Distribution

Amazonas, Antioquia, Caquetá, Cundinamarca, Quindío, Valle del Cauca (Fernández et al. 2019).

#### Camponotus arboreus

(Smith, 1858)

7C17C448-9542-548F-B0F3-B045245EC04C

##### Distribution

Amazonas, Caquetá, Cundinamarca, Tolima, Huila, Caquetá (Fernández et al. 2019).

#### Camponotus blandus

(Smith, 1858)

81FDB2CB-06C2-5AAF-A5AB-C06921053176

##### Distribution

Amazonas, Antioquia, Arauca, Atlántico, Bolívar, Boyacá, Caldas, Caquetá, Casanare, Cesar, Cundinamarca, Guainía, Huila, La Guajira, Magdalena, Meta, Norte de Santander, Santander, Sucre, Tolima, Valle del Cauca (Fernández et al. 2019).

#### Camponotus bonariensis

Mayr, 1868

E25519F1-F017-5325-9DC9-BE126F3F4E0A

##### Distribution

Boyacá, Caquetá, Cundinamarca, Nariño (Fernández et al. 2019).

#### Camponotus cacicus

Emery, 1903

4D00C40D-1BB8-55F7-AE3F-786FED7082B9

##### Distribution

Amazonas, Caquetá, Quindío, Valle del Cauca (Castro et al. 2018, Fernández et al. 2019).

#### Camponotus canescens

Mayr, 1870

C32CF21E-3673-5F45-AA32-6A5047CBD3FA

##### Distribution

Amazonas, Antioquia, Chocó, Caquetá, Cundinamarca, Huila, Meta, Quindío, Risaralda, Valle del Cauca (Fernández et al. 2019).

#### Camponotus coloratus

Forel, 1904

022DECC6-3C6A-5B1E-B233-3FBDAE4622A6

##### Distribution

Antioquia, Bolívar, Boyacá, Caquetá Casanare, Cundinamarca, Huila, La Guajira, Magdalena, Meta, Santander, Sucre, Tolima, Vichada (Fernández et al. 2019).

#### Camponotus conspicuus

(Smith, 1858)

3FEBE530-ACF6-5623-9938-ACCC7EA96951

##### Distribution

Amazonas, Antioquia, Caquetá, Valle del Cauca (Fernández et al. 2019).

#### Camponotus excisus

Mayr, 1870

4E71720A-3B3C-5BA8-9C7B-1945B5CAB4BF

##### Distribution

Amazonas, Antioquia, Caquetá, Cauca, Chocó, Cundinamarca, Guaviare, Huila, Magdalena, Meta, Valle del Cauca (Fernández et al. 2019).

#### Camponotus fasciatellus

Dalla Torre, 1892

2AAE0E05-55C0-5D82-B564-B6BB024F656F

##### Distribution

Bolívar, Caquetá, Chocó, Cundinamarca, Guaviare, Huila, Meta, Tolima (Fernández et al. 2019).

#### Camponotus fastigatus

Roger, 1863

40252786-97CF-599E-90D1-58D504747CD6

##### Distribution

Caquetá, Huila (Fernández et al. 2019).

#### Camponotus femoratus

(Fabricius, 1804)

8B10174F-04BF-59F7-9C65-678E901C6B1C

##### Distribution

Amazonas, Caquetá, Guaviare, Meta, Putumayo, Vaupés (Fernández et al. 2019).

#### Camponotus indianus

Forel, 1879

86541EC2-AEEB-53FA-924A-DA14511D6766

##### Distribution

Antioquia, Bolívar, Boyacá, Caquetá, Caldas, Cauca, Chocó, Cundinamarca, Huila, La Guajira, Nariño, Norte de Santander, Magdalena, Meta, Quindío, Sucre, Tolima, Valle del Cauca (Chacón de Ulloa and Abadía 2014, Fernández et al. 2019).

#### Camponotus latangulus

Roger, 1863

03989152-5347-5B32-97C1-C67EB30AC127

##### Distribution

Amazonas, Caquetá, Meta (Castro et al. 2018, Fernández et al. 2019).

#### Camponotus leydigi

Forel, 1886

56EBC7F1-7509-5BB4-8425-D67D43DBBE82

##### Distribution

Amazonas, Caquetá, Cundinamarca, Guaviare, Huila, Meta, Nariño, Vichada, Valle del Cauca (Fernández et al. 2019).

#### Camponotus mus

Roger, 1863

17947F4C-C87E-5821-A9FD-0519E2C867D3

##### Distribution

Amazonas, Antioquia, Boyacá, Caquetá, Huila, Santander, Tolima, Valle del Cauca (Fernández et al. 2019).

#### Camponotus nitidior

(Santschi, 1921)

62460C6A-CDE0-57C2-8B56-B19C1A16E65D

##### Distribution

Amazonas, Antioquia, Caquetá, Chocó, Risaralda, Valle del Cauca (Castro et al. 2018, Fernández et al. 2019).

#### Camponotus novogranadensis

Mayr, 1870

BF88DD2D-F172-5FC5-9A18-0B840C55D8DB

##### Distribution

Amazonas, Antioquia, Caquetá, Casanare, Chocó, Córdoba, Cundinamarca, Guaviare, Huila, La Guajira, Magdalena, Meta, Quindío, Tolima, Valle del Cauca, Vichada (Fernández et al. 2019).

#### Camponotus rapax

(Fabricius, 1804)

54E66125-ECEE-5CCB-98F9-A9F1E641203C

##### Distribution

Amazonas, Caquetá, Guaviare, Meta (Fernández et al. 2019).

#### Camponotus renggeri

Emery, 1894

7B26FD66-EA29-54EB-B429-84019A4DDAFB

##### Distribution

Amazonas, Caquetá, Cundinamarca, Huila, Meta (Fernández et al. 1996, Fernández et al. 2019).

#### Camponotus rufipes

(Fabricius, 1775)

A39CA743-F14D-5978-9CBA-B8638995F858

##### Distribution

Amazonas, Boyacá, Caquetá, Cundinamarca, Huila, Guaviare, Meta, Nariño, Vichada (Fernández et al. 2019).

#### Camponotus senex

(Smith, 1858)

3795E185-91B6-5541-9C85-27FBE9D6CFB1

##### Distribution

Amazonas, Boyacá, Casanare, Caquetá, Cauca, Cundinamarca, Huila, Meta, Valle del Cauca (Arenas-Clavijo and Armbrecht 2019, Fernández et al. 2019).

#### Camponotus sericeiventris

(Guérin-Méneville, 1838)

A9B6E876-366C-5EE1-9232-68AB2AD55AF8

##### Distribution

Amazonas, Antioquia, Caquetá, Chocó, Huila, Meta, Nariño, Tolima, Valle del Cauca (Fernández et al. 2019).

#### Camponotus sexguttatus

(Fabricius, 1793)

5226A51C-74C4-5D25-96EA-C8C735439373

##### Distribution

Antioquia, Amazonas, Arauca, Caquetá, Huila, Magdalena, Meta, Nariño (Fernández et al. 2019).

#### Camponotus sphenoidalis

Mayr, 1870

65BA58F7-F1E9-5F10-9918-8BD454274433

##### Distribution

Antioquia, Boyacá, Caquetá, Meta, Nariño, Quindío, Risaralda, Tolima, Valle del Cauca (Fernández et al. 2019).

#### Gigantiops destructor

(Fabricius, 1804)

2868BA98-F64C-5502-8215-87C75CB7548F

##### Distribution

Amazonas, Caquetá, Meta, Nariño, Tolima, Vichada (Bustos 1994, Fernández et al. 1996, Castro et al. 2018, Fernández et al. 2019).

#### Myrmelachista schumanni

Emery, 1890

33EB88B3-D0D0-5B4C-8AFA-7C4EC206E175

##### Distribution

Caquetá (Fernández et al. 2019).

##### Notes

New record from Caquetá

#### Paratrechina longicornis

(Latreille, 1802)

767BDE5F-6A6B-51FF-A235-A9578D16A7A9

##### Distribution

Amazonas, Caquetá, Casanare, Huila, Cundinamarca, Meta (Fernández et al. 1996, Fernández et al. 2019).

#### Acromyrmex aspersus

(Smith, 1858)

9C78DF61-3F1C-5577-8880-AC9666735286

##### Distribution

Antioquia, Bolívar, Caldas, Caquetá, La Guajira, Guaviare, Huila, Norte de Santander, Nariño, Putumayo, Quindío, Valle del Cauca (Fernández et al. 2019).

#### Acromyrmex coronatus

(Fabricius, 1804)

A6CFAF24-AE18-5101-99C8-27A2C408BFC4

##### Distribution

Antioquia, Caquetá, Casanare, Cundinamarca, Magdalena, Meta, Nariño, Norte de Santander, Valle del Cauca (Fernández et al. 2019).

#### Acromyrmex hystrix

(Latreille, 1802)

64E8D3D1-1D5D-5AB9-932B-B4432E8A4D83

##### Distribution

Amazonas, Boyacá, Caquetá, Cauca, Cundinamarca, Meta, Nariño, Putumayo, Valle del Cauca, Vaupés, Vichada (Fernández et al. 2019).

#### Acromyrmex nobilis

Santschi, 1939

E3ADD255-6EE9-50AF-B546-0EF6AF753671

##### Distribution

Amazonas, Caquetá, Huila, Nariño, Vaupés, Vichada (Fernández et al. 2019).

#### Acromyrmex octospinosus

(Reich, 1793)

2EEB75E0-19B3-5E9C-AC4C-5E28B388B50E

##### Distribution

Amazonas, Antioquia, Atlántico, Bolívar, Boyacá, Caldas, Caquetá, Casanare, Cauca, Cesar, Chocó, Córdoba, Cundinamarca, Guainía, Guaviare, Huila, Magdalena, Nariño, Norte de Santander, Quindío, Risaralda, Santander, Tolima, Valle del Cauca, Vichada (Fernández et al. 2019).

#### Adelomyrmex striatus

Fernández, 2003

572B81CA-F0A5-5A12-BF05-85A9ABD7A99A

##### Distribution

Amazonas, Caquetá (Fernández et al. 2019).

##### Notes

New record from Caquetá.

#### Apterostigma angustum

Lattke, 1997

6B202651-A187-59FA-94B9-D2584B63EB7B

##### Distribution

Antioquia, Atlántico, Caquetá, Chocó, Sucre (Mera-Rodríguez et al. 2020).

#### Apterostigma auriculatum

Wheeler, 1925

37AC65D2-C36F-57B0-A50F-2F4F5D1CCA8D

##### Distribution

Present throughout the country (Fernández et al. 2019).

#### Apterostigma goniodes

Lattke, 1997

3AE63E69-2742-575D-B5C7-598F9278087E

##### Distribution

Antioquia, Caquetá, Cauca, Sucre (Castro et al. 2018, Fernández et al. 2019).

#### Apterostigma jubatum

Wheeler, 1925

D0704EA9-3C6F-5E5C-8DDA-A63134B98B2A

##### Distribution

Present throughout the country (Fernández et al. 2019).

#### Apterostigma manni

Weber, 1938

1ED71218-C324-5AA6-978C-A0BE28E6AE1E

##### Distribution

Present throughout the country (Fernández et al. 2019).

#### Apterostigma megacephala

Lattke, 1999

2754145C-D964-5BEB-83F8-A5B36B04F58F

##### Distribution

Caquetá, Meta (Castro et al. 2018, Fernández et al. 2019).

#### Apterostigma peruvianum

Wheeler, 1925

7A332871-4075-586A-9683-A9CAF8A008B0

##### Distribution

Caquetá, Valle del Cauca (Mera-Rodríguez et al. 2020).

#### Apterostigma urichii

Forel, 1893

9D50D36A-69C8-5E15-B721-FA3E8B91D3ED

##### Distribution

Present throughout the country (Fernández et al. 2019).

#### Atta cephalotes

(Linnaeus, 1758)

ABC135F1-C880-534B-A22F-DD7EB2FDE8DE

##### Distribution

Amazonas, Antioquia, Arauca, Bolívar, Boyacá, Caldas, Caquetá, Casanare, Cauca, Cesar, Chocó, Córdoba, Cundinamarca, Guainía, Guaviare, Huila, Magdalena, Meta, Nariño, Norte de Santander, Putumayo, Quindío, Risaralda, Santander, Tolima, Valle del Cauca, Vaupés, Vichada (Fernández et al. 2019).

#### Atta colombica

Guérin-Méneville, 1844

65784A2B-DF1D-5026-93B1-3B391CFEA512

##### Distribution

Antioquia, Boyacá, Caldas, Caquetá, Casanare, Chocó, Córdoba, Cundinamarca, Huila, La Guajira, Magdalena, Meta, Nariño, Putumayo, Quindío, Risaralda, Santander, Sucre, Tolima, Valle del Cauca, Vichada (Fernández et al. 2019).

#### Atta sexdens

(Linnaeus, 1758)

5BCC4153-75EA-57D7-B1AC-DD2EA23DC661

##### Distribution

Amazonas, Bolívar, Boyacá, Caldas, Caquetá, Casanare, Chocó, Cundinamarca, Guainía, La Guajira, Magdalena, Meta, Santander, Tolima, Valle del Cauca, Vaupés (Fernández et al. 1996, Fernández et al. 2019).

#### Basiceros conjugans

Brown, 1974

E4B51290-6636-5FC5-BDD6-6950A2D53C2A

##### Distribution

Amazonas, Caquetá, Meta, Nariño (Fernández et al. 2019).

##### Notes

New record from Caquetá.

#### Basiceros disciger

(Mayr, 1887)

12101A91-5388-563B-823B-FB4ED938EE67

##### Distribution

(Fernández et al. 2019).Caquetá, Meta

##### Notes

New record from Caquetá.

#### Basiceros scambognathus

(Brown, 1949)

28B6A16F-45D6-5CA6-83DD-97528CB5E21E

##### Distribution

Amazonas, Caquetá (Fernández et al. 2019).

##### Notes

New record from Caquetá.

#### Blepharidatta brasiliensis

Wheeler, 1915

A92FF36B-9002-5660-BB86-10349E6F51C3

##### Distribution

Amazonas, Caquetá, Vaupés (Fernández et al. 2019).

#### Cardiocondyla nuda

(Mayr, 1866)

02A33EA5-17DE-5865-9C7C-7AE4AF35CD04

##### Distribution

Amazonas, Caquetá, Cundinamarca, Huila, Meta, Santander, Valle del Cauca (Castro et al. 2018, Fernández et al. 2019).

#### Carebara brevipilosa

Fernández, 2004

DEBF2BE8-05A1-5ADD-B88F-CFF0FC702EC2

##### Distribution

Amazonas, Caquetá, Nariño (Castro et al. 2018, Fernández et al. 2019).

#### Carebara coeca

Fernández, 2004

312141B8-9503-57B8-9B3D-C889E150DD9B

##### Distribution

Caquetá, Putumayo (Fernández et al. 2019).

#### Carebara coqueta

Fernández, 2006

CFD33328-D05A-52D8-94D6-064228695AC7

##### Distribution

Caquetá (Fernández 2006).

#### Carebara globularia

Fernández, 2004

17564635-D6EF-557E-B2F3-B45E8CD57235

##### Distribution

Amazonas, Caquetá, Nariño (Fernández et al. 2019).

#### Carebara urichi

(Wheeler, 1922)

1870E429-E52D-5B35-AD81-578949A49C1D

##### Distribution

Amazonas, Caquetá, Chocó, Magdalena (Fernández et al. 2019).

#### Cephalotes atratus

(Linnaeus, 1758)

C8F4481A-6060-5E71-AA31-ABA460C8D896

##### Distribution

Amazonas, Antioquia, Atlántico, Arauca, Bolívar, Boyacá, Caldas, Caquetá, Casanare, Cauca, Cesar, Chocó, Córdoba, Cundinamarca, Guainía, Guaviare, La Guajira, Magdalena, Meta, Nariño, Norte de Santander, Putumayo, Santander, Sucre, Tolima, Vichada, Valle del Cauca, Vaupés (Fernández et al. 2019).

#### Cephalotes clypeatus

(Fabricius, 1804)

80B7216F-6AA1-544B-BDAB-DCF2D51B3B2C

##### Distribution

Amazonas, Caquetá, Casanare, Córdoba, Cundinamarca, Meta, Santander, Sucre, Valle del Cauca (Fernández et al. 2019).

#### Cephalotes cordatus

(Smith, 1853)

1BE7262E-CA51-5F3C-82E0-42598BC00B14

##### Distribution

Amazonas, Caldas, Caquetá, Meta, Putumayo, Vaupés (Fernández et al. 2019).

#### Cephalotes grandinosus

(Smith, 1860)

B1AA9EF2-F498-5B9F-B417-2777F760954B

##### Distribution

Amazonas, Bolívar, Caquetá, Chocó, Cundinamarca, Huila, Magdalena, Meta, Putumayo, Tolima, Vaupés, Vichada (Fernández et al. 2019).

#### Cephalotes inaequalis

(Mann, 1916)

69B8220A-1A10-597B-9A5D-55B7B82E6FC6

##### Distribution

Amazonas, Caquetá, Vaupés (Fernández et al. 2019).

#### Cephalotes laminatus

(Smith, 1860)

E6EE1464-2804-5F1F-9CFA-2EE0AF7AAC0C

##### Distribution

Amazonas, Caquetá, Guaviare, Putumayo (Fernández et al. 2019).

#### Cephalotes maculatus

(Smith, 1876)

457CE77E-D6A8-5D25-A681-3A8827A94455

##### Distribution

Amazonas, Antioquia, Arauca, Bolívar, Caldas, Caquetá, Casanare, Chocó, Cundinamarca, Guaviare, Huila, Magdalena, Meta, Nariño, Putumayo, Risaralda, Santander, Sucre, Tolima, Valle del Cauca, Vichada (Fernández et al. 2019).

#### Cephalotes manni

(Kempf, 1951)

E806C3F7-E681-5569-8329-43C63CEB50ED

##### Distribution

Amazonas, Caquetá, Meta, Vaupés (Fernández et al. 2019).

#### Cephalotes opacus

Santschi, 1920

6908AA5E-9B0D-5D12-BE72-97155164E9C4

##### Distribution

Amazonas, Caquetá, Guaviare, Meta, Putumayo (Fernández et al. 2019).

#### Cephalotes placidus

(Smith, 1860)

6F796F64-CC72-5B95-9135-C34F373E4F52

##### Distribution

Amazonas, Caquetá, Guainía, Guaviare, Meta, Putumayo, Vaupés, Vichada (Fernández et al. 2019).

#### Cephalotes pusillus

(Klug, 1824)

E62911FD-FE05-55F5-B46C-1C065D561B70

##### Distribution

Amazonas, Antioquia, Arauca, Atlántico, Bolívar, Boyacá, Caquetá, Casanare, Cesar, Córdoba, Guainía, La Guajira, Guaviare, Magdalena, Meta, Sucre (Fernández et al. 2019).

#### Cephalotes simillimus

(Kempf, 1951)

9CE819FC-E9FC-5B67-9EE7-D477B07D4CCC

##### Distribution

Amazonas, Caquetá, Putumayo, Vaupés (Fernández et al. 2019).

#### Cephalotes spinosus

(Mayr, 1862)

D12F5CA0-80B9-5E93-845A-326AC8767606

##### Distribution

Amazonas, Caquetá, Guaviare, Meta, Putumayo, Vaupés (Fernández et al. 2019).

#### Cephalotes umbraculatus

(Fabricius, 1804)

BC1124F4-6B75-52EE-BE42-BF04D88BAB3E

##### Distribution

Amazonas, Antioquia, Bolívar, Caquetá, Cesar, Chocó, Huila, Magdalena, Meta, Nariño, Risaralda, Tolima (Fernández et al. 2019).

#### Crematogaster abstinens

Forel, 1899

7C9EC333-4C89-5690-A9A4-D3A6A5A02A4A

##### Distribution

Amazonas, Antioquia, Caquetá, Casanare, Cundinamarca, Huila, La Guajira, Meta, Norte de Santander, Sucre, Tolima (Fernández et al. 1996, Vergara-Navarro and Serna 2013, Castro et al. 2018, Fernández et al. 2019).

#### Crematogaster brasiliensis

Mayr, 1878

3CA392FD-32E5-5DD5-A1FF-82728346A540

##### Distribution

Caquetá, Chocó, Meta (Castro et al. 2018, Fernández et al. 2019).

#### Crematogaster carinata

Mayr, 1862

29B50AB6-FF8E-5B1A-9555-2CCC6F36CB9B

##### Distribution

Amazonas, Caquetá, Chocó, Huila, Magdalena, Meta, Valle del Cauca (Castro et al. 2018, Fernández et al. 2019).

#### Crematogaster crinosa

Mayr, 1862

1963E059-7392-5FE5-89A7-61544B518D2C

##### Distribution

Antioquia, Atlántico, Caquetá, Cesar, Cundinamarca, La Guajira, Huila, Magdalena, Meta, Norte de Santander, Santander, Tolima, VAlle del Cauca (Morgan and Mackay 2017, Castro et al. 2018, Fernández et al. 2019).

#### Crematogaster curvispinosa

Mayr, 1862

EFE9A1CE-A94E-51A1-A225-9F1D562E9873

##### Distribution

Antioquia, Caquetá, Cauca, Cundinamarca, Huila, Magdalena, Meta, Risaralda, Valle del Cauca (Fernández et al. 1996, Morgan and Mackay 2017, Fernández et al. 2019).

#### Crematogaster erecta

Mayr, 1866

3C2897FF-9844-5CB6-89EB-129407697F88

##### Distribution

Caquetá, Cesar, Chocó, Cundinamarca, Meta, Huila, Valle del Cauca (Fernández et al. 1996, Longino 2003, Castro et al. 2018, Fernández et al. 2019).

#### Crematogaster limata

Smith, 1858

762F9BBF-7873-522F-942A-E18033E52350

##### Distribution

Antioquia, Caquetá, Cundinamarca, Huila, Magdalena, Meta, Putumayo, Risaralda, Valle del Cauca (Castro et al. 2018, Fernández et al. 2019).

#### Crematogaster longispina

Emery, 1890

5339451F-7602-5B0D-8B4D-EADCF4361C00

##### Distribution

Amazonas, Caquetá, Cundinamarca (Castro et al. 2018).

#### Crematogaster sotobosque

Longino, 2003

572B47DD-CD67-5EA5-A19B-CAE9FBB8EB16

##### Distribution

Caquetá, Chocó, Risaralda, Valle del Cauca (Castro et al. 2018, Fernández et al. 2019).

#### Cyphomyrmex hamulatus

Weber, 1938

E904CE4D-F086-5193-A8BD-2AAB5E02C7BE

##### Distribution

Antioquia, Caquetá, Sucre, Valle del Cauca (Mera-Rodríguez et al. 2020).

#### Cyphomyrmex kirbyi

Mayr, 1887

5B3F41EE-3D32-52E6-91CD-B1983CD31DC6

##### Distribution

Caquetá, Magdalena (Fernández et al. 2019).

#### Cyphomyrmex laevigatus

Weber, 1938

5E9844B3-B4C8-527C-8CA6-43F66D7424AA

##### Distribution

Amazonas, Caquetá, Meta (Fernández et al. 2019).

#### Cyphomyrmex minutus

Mayr, 1862

2B43B5DC-7C85-547A-9F0D-AF9FC9C1B9AE

##### Distribution

Caldas, Caquetá, Cauca, La Guajira, Risaralda (Castro et al. 2018, Fernández et al. 2019).

#### Cyphomyrmex peltatus

Kempf, 1966

320DF2C7-5F1C-5C6E-ABCC-AB1BD18807EF

##### Distribution

Caquetá, Huila, Putumayo, Quindío, Vichada (Castro et al. 2018, Fernández et al. 2019).

#### Cyphomyrmex rimosus

(Spinola, 1851)

02A9A931-C978-513D-93EB-0290957C8E8E

##### Distribution

Antioquia, Bolívar, Caquetá, Cauca, Chocó, Cundinamarca, La Guajira, Magdalena, Meta, Risaralda, Tolima, Valle del Cauca (Fernández et al. 2019).

#### Cyphomyrmex salvini

Forel, 1899

D2693E6F-9407-5D93-8D56-0C4F2C5D8764

##### Distribution

Caquetá, Cauca, Chocó, Magdalena, Meta, Quindío, Risaralda, Valle del Cauca (Fernández et al. 2019).

#### Daceton armigerum

(Latreille, 1802)

2122A7D6-8068-5C60-B804-BB936C22E4B0

##### Distribution

Amazonas, Caquetá, Cesar, Guainía, Guaviare, Meta, Vichada (Fernández et al. 2019).

#### Eurhopalothrix alopeciosa

Brown & Kempf, 1960

53F4D4C4-D525-508F-8B1B-245F342F24D7

##### Distribution

Present throughout the country (Fernández et al. 2019).

#### Hylomyrma blandiens

Kempf, 1961

FF710D22-633D-512C-8876-39D5BADE3013

##### Distribution

Amazonas, Caquetá, Meta (Ulysséa and Brandão 2021).

#### Hylomyrma dandarae

Ulysséa, 2021

3CF0ADE1-C413-559C-A7CB-0AD8979C53BC

##### Distribution

Caquetá, Putumayo (Ulysséa and Brandão 2021).

#### Hylomyrma dolichops

Kempf, 1973

C3197AE7-E5F3-54B7-B67D-DAD0E3F6D2C4

##### Distribution

Amazonas, Caquetá (Fernández et al. 1996, Fernández et al. 2019).

#### Hylomyrma immanis

Kempf, 1973

40EB5B98-D1E0-53E7-9CCA-4D81F914BC85

##### Distribution

Amazonas, Caquetá (Castro et al. 2018, Fernández et al. 2019).

#### Hylomyrma sagax

Kempf, 1973

250B1EF7-EB9B-596F-9D1E-38E927CC033A

##### Distribution

Amazonas, Caquetá (Castro et al. 2018, Fernández et al. 2019).

#### Hylomyrma transversa

Kempf, 1973

6F8B3452-9925-5F6F-86BA-B3B7FF5E7000

##### Distribution

Caquetá, Cauca, Guaviare (Ulysséa and Brandão 2021).

#### Hylomyrma versuta

Kempf, 1973

3E26C337-A187-58CB-AD80-BA74B82BF774

##### Distribution

Amazonas, Caquetá (Fernández et al. 1996, Fernández et al. 2019).

#### Kempfidris inusualis

(Fernández, 2007)

C424652E-1095-5F49-99D1-4A8EDF3E0720

##### Distribution

Amazonas, Caquetá (Fernández et al. 2019).

##### Notes

New record from Caquetá.

#### Lachnomyrmex pilosus

(Weber, 1950)

D5920BB2-AD53-59B0-9D85-EDD9F4D88F12

##### Distribution

Caquetá, Meta, Nariño (Fernández et al. 2019).

##### Notes

New record from Caquetá.

#### Lenomyrmex inusitatus

Fernández, 2001

A45217B8-9475-546B-A59F-8297CA62F16C

##### Distribution

Caquetá, Nariño (Fernández et al. 2019).

##### Notes

New record from Caquetá.

#### Megalomyrmex drifti

Kempf, 1961

FA3550FB-367B-5897-BE48-E2844D5BB1B4

##### Distribution

Amazonas, Caquetá, Magdalena, Meta, Putumayo (Fernández et al. 2019).

#### Megalomyrmex emeryi

Forel, 1904

A6685577-807F-5549-82C3-37987A32656C

##### Distribution

Caquetá, Putumayo (Fernández et al. 2019).

#### Megalomyrmex foreli

Emery, 1890

71A40A14-8BFD-5A34-B6FD-0B884A515520

##### Distribution

Antioquia, Caquetá, Meta, Putumayo (Fernández et al. 2019).

#### Megalomyrmex incisus

Smith, 1947

1B37D106-0AD5-5245-A8FC-6D7078EBCBE2

##### Distribution

Amazonas, Caquetá, Magdalena, Meta (Fernández et al. 2019).

#### Megalomyrmex leoninus

Forel, 1885

18237654-5D1A-5D3E-996B-5DF3F7C72FFF

##### Distribution

Boyacá, Caquetá, Guaviare, Santander, Valle del Cauca (Castro et al. 2018, Fernández et al. 2019).

#### Megalomyrmex megadrifti

Boudinot et al., 2013

338F252F-AED9-5F30-BF57-5F1EE4AA9D91

##### Distribution

Caquetá, Meta (Castro et al. 2018)

#### Megalomyrmex staudingeri

Emery, 1890

B318E75A-C1DB-5B8B-8530-9CF22EBBE2E2

##### Distribution

Amazonas, Caquetá (Fernández et al. 2019).

#### Mycetomoellerius farinosus

(Emery, 1894)

0159ECB1-AAE3-5AAB-A538-863AF92B2730

##### Distribution

Caquetá

##### Notes

New record from Colombia.

#### Mycetomoellerius gaigei

(Forel, 1914)

7CCCC6C0-3FB3-5AE7-A29F-95389570A920

##### Distribution

Present throughout the country (Fernández et al. 2019).

#### Mycocepurus smithii

Forel, 1893

33C83908-F973-54F9-BC6D-0142F5C48C21

##### Distribution

Amazonas, Antioquia, Caldas, Caquetá, Huila, Magdalena, Meta, Risaralda, Valle del Cauca (Castro et al. 2018, Fernández et al. 2019).

#### Myrmicocrypta longinoda

Weber, 1938

09795F46-DBED-59AA-9451-AC04AA6D395C

##### Distribution

Amazonas, Caquetá, Putumayo (Castro et al. 2018, Fernández et al. 2019).

#### Myrmicocrypta spinosa

Weber, 1937

150E991B-FA29-5F9F-ADD0-E44A1EC8EE97

##### Distribution

Caquetá (Fernández et al. 2019).

#### Myrmicocrypta urichi

Weber, 1937

3B498C39-FE97-5EC0-9E4E-EF4456C0D8A5

##### Distribution

Antioquia, Caquetá (Mera-Rodríguez et al. 2020).

#### Nesomyrmex asper

(Emery, 1897)

F13C559C-9CDD-5E39-A93F-53FD3E7EBD29

##### Distribution

Caquetá, Magdalena (Castro et al. 2018, Fernández et al. 2019).

#### Ochetomyrmex neopolitus

Fernández, 2003

E8C0267B-3BC3-584A-83A8-242B02E2B74C

##### Distribution

Present throughout the country (Fernández et al. 2019).

#### Ochetomyrmex semipolitus

Mayr, 1878

A0F29E98-AEE1-55B4-BFE5-9DA93321ED62

##### Distribution

Present throughout the country (Fernández et al. 2019).

#### Octostruma balzani

(Emery, 1894)

4FB28315-8BE5-5DD7-95BA-F8780162988A

##### Distribution

Amazonas, Antioquia, Bolívar, Caquetá, Chocó, Cundinamarca, Guaviare, Magdalena, Meta, Quindío, Risaralda, Tolima, Valle del Cauca (Fernández et al. 2019).

#### Octostruma impressa

Palacio, 1997

069EF99D-35E4-584B-830F-7B2B3842F123

##### Distribution

Antioquia, Caquetá, Risaralda, Magdalena, Valle del Cauca (Castro et al. 2018, Fernández et al. 2019).

#### Octostruma rugifera

(Mayr, 1887)

1AAB742D-0E11-5D90-9FFF-F8DFC6E3801A

##### Distribution

Present throughout the country (Fernández et al. 2019).

#### Oxyepoecus ephippiatus

Albuquerque & Brandão, 2004

1B35EA57-3AF1-585A-98FB-6772061CCC42

##### Distribution

Caquetá

##### Notes

New record from Colombia.

#### Paratrachymyrmex diversus

(Mann, 1916)

CC9C4969-AB45-5BE1-B9F1-2A27FA475824

##### Distribution

Caquetá (Mera-Rodríguez et al. 2020).

#### Pheidole arachnion

Wilson, 2003

49D4258E-C94B-553C-85E9-CFDB324BD333

##### Distribution

Caquetá (Guerrero et al. 2022)

#### Pheidole gertrudae

Forel, 1886

291A2404-1CEF-5C51-8EBC-9ED14D2988F9

##### Distribution

Amazonas, Caquetá (Castro et al. 2018, Fernández et al. 2019).

#### Pogonomyrmex naegelii

Emery, 1878

D7147D50-5020-524C-AFA8-4ABB0FD934FB

##### Distribution

Amazonas, Caquetá, Meta (Fernández et al. 1996, Fernández et al. 2019).

#### Procryptocerus convexus

Forel, 1904

708DDD3C-8184-5509-A84F-53AC0F22B795

##### Distribution

Caquetá (Fernández et al. 2019).

#### Procryptocerus scabriusculus

Forel, 1899

C582268A-2A89-5B45-9406-D507AD05CF5D

##### Distribution

Antioquia, Caquetá, Caldas, Cundinamarca, Quindío, Risaralda (Castro et al. 2018, Fernández et al. 2019).

#### Procryptocerus schmitti

Forel, 1901

C657FDB7-6092-5B88-A560-F482BE4C87B4

##### Distribution

Caquetá (Fernández et al. 2019).

#### Procryptocerus spiniperdus

Forel, 1899

DD104938-35D8-53E5-87CF-6E584BF5EC82

##### Distribution

Caquetá, Vaupés (Fernández et al. 2019).

#### Sericomyrmex amabilis

Wheeler, 1925

72B18D46-377A-5F03-96C8-6BE471A1F2BA

##### Distribution

Amazonas, Antioquia, Caquetá, Cauca, Chocó, Cundinamarca, Magdalena, Tolima, Risaralda, Valle del Cauca (Mera-Rodríguez et al. 2020).

#### Sericomyrmex bondari

Borgmeier, 1937

9C085B48-C0C8-5B5D-A95A-A08D1E25FED4

##### Distribution

Amazonas, Caquetá, Meta, Putumayo, Vaupés, Vichada (Fernández et al. 2019).

#### Sericomyrmex mayri

Forel, 1912

3A5DFEB0-194D-5A2C-8DE3-0531F3012AF0

##### Distribution

Amazonas, Caquetá, Meta, Putumayo, Vaupés, Vichada (Fernández et al. 2019).

#### Sericomyrmex parvulus

Forel, 1912

99BCCCB4-7D7E-5AC2-A2D1-7CDA233E0FED

##### Distribution

Amazonas, Caquetá (Fernández et al. 2019).

#### Solenopsis geminata

(Fabricius, 1804)

CA202759-5085-589B-842E-D4888A0E3D85

##### Distribution

Antioquia, Bolívar, Caquetá, Chocó, La Guajira, Santander, Risaralda, Valle del Cauca (Castro et al. 2018, Fernández et al. 2019).

#### Stegomyrmex manni

Smith, 1946

95312F88-EAE0-5593-9A27-C85F9606000D

##### Distribution

Antioquia, Bolivar, Caquetá, Sucre (Fernández et al. 2019, Camargo-Vanegas et al. 2024).

##### Notes

New record from Caquetá.

#### Strumigenys cassicuspis

(Bolton, 2000)

2C4B05A9-8889-5AA5-B56B-B3D46D75509A

##### Distribution

Caquetá (Fernández et al. 2019).

#### Strumigenys cordovensis

Mayr, 1887

E1F3EC9E-7FE1-5473-B403-77C7815308D4

##### Distribution

Arauca, Caquetá, Cundinamarca, Magdalena, Meta, Valle del Cauca (Fernández et al. 2019).

#### Strumigenys decipula

(Bolton, 2000)

37496F37-6742-5918-B56F-8F8272EE23FA

##### Distribution

Boyacá, Caquetá (Fernández et al. 2019).

#### Strumigenys denticulata

Mayr, 1887

5F725D29-33B6-51C0-9B56-65729A507BCB

##### Distribution

Amazonas, Antioquia, Arauca, Caldas, Caquetá, Cauca, Cundinamarca, La Guajira, Magdalena, Meta, Nariño, Putumayo, Sucre, Valle del Cauca, Vaupés, Vichada (Fernández et al. 2019).

#### Strumigenys enopla

(Bolton, 2000)

02E1FE85-02F8-53E1-9DE1-153DAA418957

##### Distribution

Boyacá, Caquetá, Cauca, Huila, Magdalena, Nariño, Norte de Santander, Santander (Fernández et al. 2019).

#### Strumigenys fridericimuelleri

Forel, 1886

DE55762E-66D1-5506-A174-C0DEC05FD0C7

##### Distribution

Caquetá (Fernández et al. 2019).

#### Strumigenys gundlachi

(Roger, 1862)

75673DFE-2EE6-5E5B-B476-EC6A357B651A

##### Distribution

Antioquia, Caldas, Caquetá, Cauca, Chocó, Cundinamarca, Huila, Magdalena, Meta, Nariño, Norte de Santander, Putumayo, Quindío, Risaralda, Santander, Tolima, Valle del Cauca (Fernández et al. 2019).

#### Strumigenys incuba

Bolton, 2000

4F5B3690-6C9A-5510-BD7F-B7181D92659B

##### Distribution

Caquetá, Cauca, Putumayo (Fernández et al. 2019).

##### Notes

New record from Caquetá.

#### Strumigenys interfectiva

Lattke & Goitía, 1997

738B45AB-5C28-505C-B6C8-A54D11BC1C7F

##### Distribution

Antioquia, Caquetá, Nariño (Castro et al. 2018, Fernández et al. 2019).

#### Strumigenys nubila

Lattke & Goitía, 1997

D1E513FB-97C8-57A7-B3C0-0589D5EFFE25

##### Distribution

Caquetá, Caldas, Quindío, Risaralda, Valle del Cauca (Fernández et al. 2019).

#### Strumigenys precava

Brown, 1954

37ECCF7C-91CC-51BD-B838-75EC596F60C8

##### Distribution

Amazonas, Caquetá, Cundinamarca, Meta, Putumayo (Fernández et al. 2019).

#### Strumigenys prospiciens

Emery, 1906

1A27079C-9102-55FC-9F51-70E648A635B8

##### Distribution

Caquetá

##### Notes

New record from Colombia.

#### Strumigenys raptans

(Bolton, 2000)

2B45B4B6-64F7-5F6A-A752-C2CB6679E061

##### Distribution

Caldas, Caquetá, Cundinamarca, Huila, Meta, Nariño, Quindío, Risaralda, Santander, Sucre, Valle del Cauca, Vichada (Fernández et al. 2019).

#### Strumigenys trinidadensis

Wheeler, 1922

5C0AA8B8-F752-5C1D-8C3F-545B2454097D

##### Distribution

Caquetá, Guaviare, Nariño, Quindío, Vaupés, Valle del Cauca (Fernández et al. 2019).

#### Strumigenys trudifera

Kempf & Brown, 1969

2DB90CBC-0C15-5C17-89EC-56C59269FC84

##### Distribution

Amazonas, Caquetá, Putumayo, Nariño, Meta, Vaupés (Fernández et al. 2019).

#### Strumigenys zeteki

(Brown, 1959)

50E78E6D-3B3D-58DE-8B52-7F6FAA940FB3

##### Distribution

Amazonas, Caldas, Caquetá, Cauca, Cundinamarca, Chocó, Magdalena, Nariño, Quindío, Santander, Tolima, Valle del Cauca (Fernández et al. 2019).

#### Talaridris mandibularis

Weber, 1941

68B66D6F-364F-590A-990E-F9F7E71A89CF

##### Distribution

Caquetá (García et al. 2020).

#### Tranopelta gilva

Mayr, 1866

FA7CD190-2DDF-53B3-AD66-E0F546572CB8

##### Distribution

Antioquia, Caldas, Caquetá, Cauca, Chocó, La Guajira, Magdalena, Nariño, Tolima, Valle del Cauca (Fernández et al. 1996, Fernández 2003b, Castro et al. 2018, Fernández et al. 2019).

#### Wasmannia auropunctata

(Roger, 1863)

F018D283-FB51-5184-8F08-F9075AB67733

##### Distribution

Amazonas, Antioquia, Bolívar, Caldas, Caquetá, Cauca, Cesar, Chocó, Cundinamarca, Huila, La Guajira, Magdalena, Meta, Putumayo, Risaralda, Tolima, Valle del Cauca (Castro et al. 2018, Fernández et al. 2019).

#### Paraponera clavata

(Fabricius, 1775)

A5480E27-1409-518A-99EA-11C46D922766

##### Distribution

Amazonas, Antioquia, Caldas, Caquetá, Casanare, Cauca, Chocó, Cundinamarca, Meta, Nariño, Putumayo, Vaupés, Valle del Cauca (Fernández et al. 2019).

#### Anochetus bispinosus

(Smith, 1858)

B63B7B03-0D30-5479-80C6-17151C02BA38

##### Distribution

Amazonas, Caquetá, Guaviare, Magdalena, Meta, Nariño, Putumayo, Valle del Cauca, Vaupés (Fernández et al. 2019).

#### Anochetus diegensis

Forel, 1912

F3123F43-6613-5F84-BE02-515678AD72C5

##### Distribution

Antioquia, Bolívar, Caquetá, La Guajira, Magdalena, Meta, Valle del Cauca (Castro et al. 2018, Fernández et al. 2019).

#### Anochetus emarginatus

(Fabricius, 1804)

3142F547-0121-583D-A632-239AA09246D9

##### Distribution

Amazonas, Bolívar, Caquetá, Chocó, La Guajira, Magdalena, Meta, Nariño, Putumayo, Valle del Cauca, Vichada (Fernández et al. 2019).

#### Anochetus horridus

Kempf, 1964

533EBB19-693D-5B3E-A8F8-A94099534A34

##### Distribution

Amazonas, Caquetá, Valle del Cauca (Lozano-Zambrano et al. 2008, Fernández et al. 2019).

#### Anochetus inermis

André, 1889

885AD15D-3DC2-5BF8-B162-C4FEA34F5539

##### Distribution

Amazonas, Caquetá, Meta, Putumayo, Risaralda, Valle del Cauca (Fernández et al. 2019).

#### Anochetus mayri

Emery, 1884

007C6381-3FB9-5DA2-B78C-1841A64C02B2

##### Distribution

Amazonas, Caquetá, Meta, Putumayo, Risaralda, Valle del Cauca (Fernández et al. 2019).

#### Centromyrmex alfaroi

Emery, 1890

E493939D-DCB8-5472-AA19-61F197D70CE4

##### Distribution

Caquetá (Fernández et al. 2019).

#### Centromyrmex brachycola

(Roger, 1861)

A9D7E39A-1188-5495-B87E-762CD9C2D45C

##### Distribution

Amazonas, Caquetá, Meta (Fernández et al. 2019).

#### Centromyrmex gigas

Forel, 1911

5ECB38FA-1152-5DDE-A889-A5939B3FA6F3

##### Distribution

Caquetá

##### Notes

New record for Colombia.

#### Dinoponera longipes

Emery, 1901

BE545A83-5DA5-5105-98F7-E45CCF2B753E

##### Distribution

Amazonas, Caquetá (Dias and Lattke 2021).

#### Hypoponera distinguenda

(Emery, 1890)

69D21CB5-58E7-515B-9A8D-92DC250BB5EF

##### Distribution

Amazonas, Caquetá, Cundinamarca, La Guajira, Magdalena, Meta, Putumayo, Valle del Cauca (Castro et al. 2018, Fernández et al. 2019).

#### Leptogenys amazonica

Borgmeier, 1930

A59473E3-68D4-5678-B2CB-231BB929D49F

##### Distribution

Caquetá (Sanabria-Blandón and Chacón de Ulloa 2012, Fernández et al. 2019).

#### Leptogenys amu

Lattke, 2011

104DC9C0-8290-55B0-AC60-3BF5FEDCE1A5

##### Distribution

Caquetá (Fernández et al. 2019).

#### Leptogenys gaigei

Wheeler, 1923

D2E15E33-3477-5DC6-98B3-18728A642693

##### Distribution

Amazonas, Nariño, Caquetá (Fernández et al. 2019).

#### Leptogenys langi

Wheeler, 1923

81BC2D59-00F2-57B8-871B-59B60EF97EE7

##### Distribution

Caquetá, Nariño (Fernández et al. 2019).

#### Leptogenys linearis

(Smith, 1858)

0C6155C9-A767-5261-AF77-41EC650CEFA4

##### Distribution

Amazonas, Caquetá (Fernández et al. 2019).

#### Leptogenys phylloba

Lattke, 2011

DB26DF49-6E1A-56AA-AFBC-915A93226098

##### Distribution

Amazonas, Caquetá (Fernández et al. 2019).

#### Leptogenys ritae

Forel, 1899

E3FCC74F-0281-5FEA-A683-8A8D0E859280

##### Distribution

Bolívar, Caquetá, Magdalena, Nariño, Santander, Tolima, Vaupés (Fernández et al. 2019).

#### Leptogenys unistimulosa

Roger, 1863

4FAF9272-FD61-567B-AD3B-2C24098674BC

##### Distribution

Caquetá, Cundinamarca, Meta (Fernández et al. 2019).

##### Notes

New record from Caquetá.

#### Mayaponera arhuaca

(Forel, 1901)

743D7124-FB9D-5615-A97D-4AEB88C35356

##### Distribution

Amazonas, Bolívar, Cauca, Caquetá, Cundinamarca, Guaviare, Magdalena, Nariño, Putumayo, Valle del Cauca (Fernández et al. 2019).

#### Mayaponera becculata

(MacKay & MacKay, 2010)

198F359E-E879-50A3-9833-24E44C06D6C7

##### Distribution

Caquetá, Magdalena (Castro et al. 2018, Fernández et al. 2019).

#### Mayaponera constricta

(Mayr, 1884)

C17447FE-2934-5153-822B-5A171CF5853A

##### Distribution

Amazonas, Antioquia, Bolívar, Boyacá, Caldas, Caquetá, Cesar, Chocó, Huila, Magdalena, Meta, Nariño, Putumayo, Quindío, Risaralda, Valle del Cauca, Vaupés, Vichada (Fernández et al. 2019).

#### Neoponera apicalis

(Latreille, 1802)

F9A3FC07-4FC8-5D4A-9AEA-0B03C489A94B

##### Distribution

Amazonas, Antioquia, Bolívar, Caquetá, Casanare, Cesar, Chocó, La Guajira, Magdalena, Meta, Norte de Santander, Valle del Cauca, Vaupés (Fernández et al. 1996, Fernández et al. 2019).

#### Neoponera carbonaria

(Smith, 1858)

A7615BA0-26FA-55BC-9EF1-C1491C80FF62

##### Distribution

Antioquia, Boyacá, Caldas, Caquetá, Cauca, Cundinamarca, Huila, Nariño, Putumayo, Risaralda, Valle del Cauca (Fernández et al. 2019).

#### Neoponera carinulata

(Roger, 1861)

6D201D93-F94E-59E1-AACB-0B3FA8EAA064

##### Distribution

Amazonas, Antioquia, Atlántico, Caquetá, Cauca, Chocó, Guainía, Guaviare, Huila, Nariño, Risaralda, Valle del Cauca, Vaupés (Fernández et al. 2019).

#### Neoponera commutata

(Roger, 1860)

13D64A29-433D-5F62-9980-1D263186CC42

##### Distribution

Amazonas, Caquetá, Cauca, Guaviare, Meta, Nariño, Putumayo, Vichada (Fernández et al. 2019).

#### Neoponera crenata

(Roger, 1861)

BF05EBE8-F9F5-57FC-83E4-CAA445117884

##### Distribution

Amazonas, Boyacá, Caldas, Caquetá, Cauca, Chocó, Huila, Meta, Putumayo, Quindío, Risaralda, Sucre, Valle del Cauca, Vaupés (Fernández et al. 2019).

#### Neoponera fauveli

(Emery, 1895)

EBB5FD61-138C-5A60-B679-E04466DC77CE

##### Distribution

Antioquia, Caquetá, Cauca, Chocó, Magdalena, Nariño, Risaralda, Valle del Cauca (Fernández et al. 2019).

#### Neoponera foetida

(Linnaeus, 1758)

02B85287-9124-5908-BE4E-DA10FC8680F1

##### Distribution

Amazonas, Antioquia, Caquetá, Cauca, Meta, Valle del Cauca (Fernández et al. 2019).

#### Neoponera laevigata

(Smith, 1858)

A52829AB-8D4F-5037-830B-CEA398662E12

##### Distribution

Amazonas, Caquetá, Chocó, Cundinamarca, Putumayo, Valle del Cauca (Fernández et al. 2019).

#### Neoponera obscuricornis

(Emery, 1890)

E432297B-01FC-5086-A9AC-BC82958D915C

##### Distribution

Amazonas, Antioquia, Caquetá, Chocó, Meta, Valle del Cauca, Vichada (Fernández et al. 1996, Fernández et al. 2019).

#### Neoponera rostrata

(Emery, 1890)

FC6DC6FF-0A1A-558B-95C6-B2BF3868C50B

##### Distribution

Amazonas, Caquetá (Fernández et al. 2019).

#### Neoponera striatinodis

(Emery, 1890)

D6A66DA8-713C-5CE8-B02F-78133FF97EBA

##### Distribution

Antioquia, Caquetá, Cauca, Chocó, Meta, Putumayo, Valle del Cauca (Chacón de Ulloa et al. 2012, Fernández et al. 2019).

#### Neoponera unidentata

(Mayr, l862)

3FF7F8B2-615A-5A61-A474-EE29ED2172CB

##### Distribution

Amazonas, Caquetá, Cauca, Cundinamarca, Guaviare, Huila, Magdalena, Meta, Nariño, Norte de Santander, Putumayo, Quindío, Risaralda, Valle del Cauca (Fernández et al. 2019).

#### Neoponera verenae

(Forel, 1922)

F83344D4-7428-5141-BBAF-566B976E1AE7

##### Distribution

Amazonas, Antioquia, Bolívar, Boyacá, Caquetá, Casanare, Cauca, Chocó, Guaviare, Huila, Magdalena, Meta, Nariño, Putumayo, Risaralda, Valle del Cauca (Fernández et al. 2019).

#### Neoponera villosa

(Fabricius, 1804)

D2E0A0BF-CE16-5C17-A51E-2B15B81D59C8

##### Distribution

Amazonas, Antioquia, Atlántico, Bolívar, Boyacá, Casanare, Caquetá, Cauca, Chocó, Cundinamarca, Guaviare, Magdalena, Meta, Nariño, Putumayo, Risaralda, Santander, Vaupés, Vichada, Valle del Cauca (Fernández et al. 2019).

#### Odontomachus bauri

Emery, 1892

616E382A-D59E-5885-A398-095E108C3FD6

##### Distribution

Caquetá, Meta, Nariño, Putumayo, Quindío, Tolima, Valle del Cauca, Vichada (Castro et al. 2018).

#### Odontomachus bradleyi

Brown, 1976

95F8801C-FC65-547E-87CA-4FC766197B76

##### Distribution

Boyacá, Caquetá, Cauca, Nariño, Norte de Santander (Fernández et al. 2019).

#### Odontomachus brunneus

(Patton, 1894)

201D3F09-BAF5-543A-AB91-B6AA2EEF0B7B

##### Distribution

Amazonas, Bolívar, Caquetá, Casanare, Magdalena, Meta, Putumayo, Santander, Vaupés (Fernández et al. 2019).

#### Odontomachus chelifer

(Latreille, 1802)

4910475D-13A2-5DE0-9732-9E99C3BE8FCC

##### Distribution

Antioquia, Bolívar, Boyacá, Caquetá, Casanare, Cesar, Huila, Magdalena, Meta, Norte de Santander, Quindío, Risaralda, Santander, Sucre, Valle del Cauca (Fernández et al. 2019).

#### Odontomachus haematodus

(Linnaeus, 1758)

6054B293-3C85-596D-9900-90A31670C8D6

##### Distribution

Amazonas, Antioquia, Bolívar, Boyacá, Caquetá, Cauca, Córdoba, Chocó, Guaviare, Magdalena, Meta, Norte de Santander, Putumayo, Vaupés (Fernández et al. 2019).

#### Odontomachus hastatus

(Fabricius, 1804)

C22A7A72-9C9C-5C90-BBDD-7E542451D76A

##### Distribution

Amazonas, Caquetá, Cauca, Meta, Nariño, Putumayo, Valle del Cauca, Vaupés (Fernández et al. 1996, Fernández et al. 2019).

#### Odontomachus laticeps

Roger, 1861

5C255F54-2DC5-525E-B94B-BD1D0A7E1537

##### Distribution

Caquetá, Cundinamarca (Lozano-Zambrano et al. 2008, Fernández et al. 2019)

#### Odontomachus meinerti

Forel, 1905

21343C8D-2A80-57D8-86EE-780E3EA413C3

##### Distribution

Amazonas, Boyacá, Caquetá, Chocó, Meta, Nariño, Norte de Santander, Putumayo, Valle del Cauca, Vaupés, Vichada (Fernández et al. 2019).

#### Odontomachus opaciventris

Forel, 1899

E54394EE-3C4B-5E5C-B683-E9F27F7949C4

##### Distribution

Caquetá, Cundinamarca, Nariño, Tolima, Santander, Valle del Cauca (Castro et al. 2018, Fernández et al. 2019).

#### Odontomachus panamensis

Forel, 1899

E248E0BB-AC28-578F-A5AE-6ABF74A2D87D

##### Distribution

Caquetá, Nariño (Fernández et al. 2019).

#### Odontomachus scalptus

Brown, 1978

F5B10E53-240D-5C5B-8291-9E748CD746E4

##### Distribution

Amazonas, Caquetá, Cundinamarca (Castro et al. 2018, Fernández et al. 2019).

#### Odontomachus yucatecus

Brown, 1976

3D3CB4D5-D870-5593-85A4-A87430D07CF5

##### Distribution

Caquetá, Nariño (Fernández et al. 2019).

#### Pachycondyla crassinoda

(Latreille, 1802)

3CD8F595-9E9D-5E15-9ACA-B49857D1F2F2

##### Distribution

Amazonas, Antioquia, Arauca, Boyacá, Caquetá, Casanare, Cauca, Chocó, Cundinamarca, Guaviare, Meta, Nariño, Putumayo, Santander, Valle del Cauca, Vaupés, Vichada (Fernández et al. 2019).

#### Pachycondyla harpax

(Fabricius, 1804)

D1905A1E-823F-5ABC-93BA-423A6E26BEDA

##### Distribution

Amazonas, Antioquia, Atlántico, Bolívar, Boyacá, Caldas, Caquetá, Cauca, Chocó, Cundinamarca, Guaviare, Huila, La Guajira, Magdalena, Meta, Nariño, Norte de Santander, Sucre, Valle del Cauca, Magdalena, Putumayo, Risaralda, Santander, Tolima, Vaupés, Vichada (Fernández et al. 2019).

#### Pachycondyla impressa

(Roger, 1861)

D02A9450-21DF-5DE7-A65D-721DCC287F14

##### Distribution

Amazonas, Antioquia, Atlántico, Bolívar, Boyacá, Caldas, Caquetá, Cauca, Chocó, Huila, Magdalena, Meta, Nariño, Norte de Santander, Putumayo, Quindío, Risaralda, Santander, Tolima, Valle del Cauca (Fernández et al. 2019).

#### Platythyrea punctata

(Smith, 1858)

1B3A2B4B-F485-553B-A886-707440BA88CA

##### Distribution

Amazonas, Bolívar, Caquetá, Meta (Fernández et al. 1996, Fernández et al. 2019).

#### Pseudoponera stigma

(Fabricius, 1804)

29C114CB-C9BC-5A1D-9C17-693F644998CF

##### Distribution

Amazonas, Antioquia, Bolívar, Boyacá, Nariño, Caquetá, Cauca, Chocó, Guaviare, Huila, Magdalena, Meta, Norte de Santander, Putumayo, Quindío, Risaralda, Tolima, Valle del Cauca, Vaupés, Vichada (Fernández et al. 2019).

#### Rasopone ferruginea

(Smith, 1858)

18DD7C6E-13A6-5B8B-BFDF-729ED04CCBEE

##### Distribution

Amazonas, Antioquia, Caldas, Caquetá, Cauca, Magdalena, Nariño, Norte de Santander, Putumayo, Quindío, Risaralda, Valle del Cauca (Fernández et al. 2019).

#### Simopelta fernandezi

Mackay & Mackay, 2008

4E3E21D9-1580-5C90-BF14-FDC9F813A3F2

##### Distribution

Antioquia, Caquetá (Vergara-Navarro and Serna 2013, Fernández et al. 2019).

#### Thaumatomyrmex atrox

Weber, 1939

040ABB2C-5452-5850-9CBF-04598145BE81

##### Distribution

Amazonas, Atlántico, Bolívar, Caquetá, Cundinamarca, Magdalena, Sucre, Valle del Cauca (Fernández et al. 2019).

##### Notes

New record from Caquetá.

#### Wadeura guianensis

Weber, 1939

4E12A820-7805-5A75-8579-5574D9E4298E

##### Distribution

Amazonas, Caquetá, Cauca, La Guajira, Meta (Castro et al. 2018, Fernández et al. 2019).

#### Wadeura holmgreni

(Wheeler, 1925)

86014151-2205-5EF9-BF33-F6D736CEA794

##### Distribution

Antioquia, Caquetá, Meta (Vergara-Navarro and Serna 2013, Castro et al. 2018, Branstetter and Longino 2022)

#### Wadeura holmgrenita

Branstetter & Longino, 2022

5EA1CDB5-581B-57A9-8987-A08ADC3F7744

##### Distribution

Caquetá

##### Notes

New record from Colombia.

#### Wadeura pauli

(Fernandes & Delabie, 2019)

010D2E41-7FD7-5AC8-A981-EA17E9796989

##### Distribution

Caquetá

##### Notes

New record from Colombia.

#### Discothyrea neotropica

Bruch, 1919

79126E33-55A1-56FC-98D2-8BC484056F40

##### Distribution

Caquetá, Bolívar, Magdalena (Fernández et al. 2019).

#### Discothyrea denticulata

Weber, 1939

0D754263-AA83-5FC1-A8AB-5D9DA43960E9

##### Distribution

Amazonas, Caquetá, Cauca, Nariño, Norte de Santander, Putumayo, Valle del Cauca (Fernández et al. 2019).

#### Discothyrea horni

Menozzi, 1927

154717BB-DCC0-547D-AD74-6D305B77135A

##### Distribution

Bolívar, Caldas, Caquetá, Quindío, Risaralda, Valle del Cauca (Chacón de Ulloa et al. 2012, Fernández et al. 2019).

#### Discothyrea humilis

Weber, 1939

D72432A5-5195-5B4A-994A-ED7E85AC1146

##### Distribution

Amazonas, Caquetá (Fernández et al. 2019).

#### Discothyrea sexarticulata

Borgmeier, 1954

43177EC4-1E8C-597E-9BBA-724FA39497BB

##### Distribution

Amazonas, Caquetá, Magdalena, Risaralda (Fernández et al. 2019).

#### Probolomyrmex kelleri

Oliveira & Feitosa, 2019

2E0E06C1-B6B0-5569-8A9E-62AF3F7619F1

##### Distribution

Caquetá

##### Notes

New record from Colombia.

#### Proceratium mancum

Mann, 1922

5B4DBB29-F5DD-5E68-847F-106C44272A62

##### Distribution

Antioquia, Caquetá, Quindío (Fernández et al. 2019).

#### Proceratium transitionis

de Andrade, 2003

2F709E9D-607F-5110-937F-164DB36F64F6

##### Distribution

Caquetá, Santander (Castro et al. 2018, Fernández et al. 2019).

#### Pseudomyrmex atripes

(Smith, 1860)

BB708381-EDD2-56F7-8CCC-F82227873DB1

##### Distribution

Caquetá, Chocó, Cundinamarca, Magdalena, Meta (Fernández et al. 1996, Fernández et al. 2019).

#### Pseudomyrmex boopis

(Roger, 1863)

E61391F2-7B93-595C-8EF6-D201100D9C0E

##### Distribution

Antioquia, Bolívar, Caquetá, Casanare, Cauca, Chocó, La Guajira, Magdalena, Meta, Nariño, Putumayo, Risaralda, Santander, Sucre, Tolima, Valle del Cauca (Fernández et al. 2019).

#### Pseudomyrmex cubaensis

(Forel, 1901)

25B3B0CF-EAD3-504D-91B9-0ED530D3A188

##### Distribution

Amazonas, Antioquia, Caquetá, Cauca, Magdalena, Meta (Fernández et al. 2019).

#### Pseudomyrmex dendroicus

(Forel, 1904)

BA8ED4AC-BC9A-53A6-BCC1-83DB5B63341B

##### Distribution

Antioquia, Arauca, Boyacá, Caquetá, Casanare, Cundinamarca, Meta, Putumayo (Fernández et al. 2019).

#### Pseudomyrmex depressus

(Forel, 1906)

1B36C949-B091-5B67-A1C3-ABC65598957F

##### Distribution

Antioquia, Caquetá, Magdalena (Fernández et al. 2019).

#### Pseudomyrmex elongatus

(Mayr, 1870)

2C166647-ECE6-50BE-ADE2-899FA590B345

##### Distribution

Amazonas, Antioquia, Bolívar, Caldas, Caquetá, Casanare, Cauca, Cesar, Chocó, Cundinamarca, Huila, La Guajira, Magdalena, Meta, Santander, Sucre, Valle del Cauca (Fernández et al. 2019).

#### Pseudomyrmex filiformis

(Fabricius, 1804)

A4AD3A7F-5ECD-538F-AF5E-AE25CD1DDFCE

##### Distribution

Antioquia, Caquetá, Chocó, Cundinamarca, Huila, La Guajira, Magdalena, Meta, Risaralda, Santander, Sucre, Tolima, Valle del Cauca (Fernández et al. 2019).

#### Pseudomyrmex flavidulus

(Smith, 1858)

A2D351F9-045A-5622-8242-F6FCB5B2893F

##### Distribution

Amazonas, Bolívar, Caquetá, Cundinamarca, Meta (Fernández et al. 1996, Fernández et al. 2019).

#### Pseudomyrmex godmani

(Forel, 1899)

5F77CD3B-70F6-5B90-B2B2-15CFBC6EF862

##### Distribution

Caquetá (Fernández et al. 2019).

#### Pseudomyrmex gracilis

(Fabricius, 1804)

6F95B5F5-6AC2-5A0D-BB66-A93C2F56BC13

##### Distribution

Present throughout the country (Fernández et al. 2019).

#### Pseudomyrmex maculatus

(Smith, 1855)

6E983D50-F418-5196-AEA4-8F16E901F362

##### Distribution

Caquetá, Casanare (Fernández et al. 2019).

#### Pseudomyrmex oculatus

(Smith, 1855)

5A523697-F8DF-5342-843C-FD58B1280772

##### Distribution

Amazonas, Antioquia, Arauca, Caquetá, Chocó, Cundinamarca, Magdalena, Meta, Santander, Tolima, Valle del Cauca, Vaupés, Vichada (Fernández et al. 2019).

#### Pseudomyrmex pallens

(Mayr, 1870)

E49A3068-0046-5998-B511-47DC08EC3A98

##### Distribution

Antioquia, Caquetá, Cundinamarca, Huila, La Guajira, Magdalena, Meta, Quindío, Risaralda, Valle del Cauca (Fernández et al. 2019).

#### Pseudomyrmex perboscii

(Guérin-Méneville, 1844)

7BBED293-2BFF-508A-8231-4D2F58A240C2

##### Distribution

Amazonas, Bolívar, Caquetá, Cundinamarca, Huila, Magdalena, Meta (Fernández et al. 1996, Fernández et al. 2019).

#### Pseudomyrmex rubiginosus

(Stitz, 1913)

9B53B99F-B536-5A23-805F-0FEBFFB3F7B1

##### Distribution

Amazonas, Caquetá (Fernández et al. 1996, Fernández et al. 2019).

#### Pseudomyrmex spiculus

Ward, 1989

8960654C-D122-5299-AA88-E3615333F3FE

##### Distribution

Amazonas, Caquetá, Chocó, Magdalena (Fernández et al. 2019).

#### Pseudomyrmex tenuis

(Fabricius, 1804)

23B4D51E-C9F0-5DB7-91D4-6016C39546B1

##### Distribution

Amazonas, Antioquia, Caquetá, Chocó, Cundinamarca, Meta, Putumayo, Vaupés (Fernández et al. 2019).

#### Pseudomyrmex tenuissimus

(Emery, 1906)

C628EBB3-9158-51D2-87CA-EB6F2D96ABDD

##### Distribution

Antioquia, Caquetá, Cundinamarca, Huila, Magdalena, Meta, San Andrés y Providencia, Santander, Sucre, Valle del Cauca (Fernández et al. 2019).

#### Pseudomyrmex terminalis

(Smith, 1877)

7D458091-4CB0-5594-863C-2D15BA6393EA

##### Distribution

Amazonas, Caquetá (Fernández et al. 1996, Fernández et al. 2019).

#### Pseudomyrmex termitarius

(Smith, 1855)

22BCFBFD-7928-53E9-82E2-4F985ADD5BF0

##### Distribution

Amazonas, Antioquia, Caquetá, Casanare, Cauca, Cesar, Córdoba, Cundinamarca, Guainía, Huila, La Guajira, Magdalena, Meta, Nariño, Norte de Santander, Putumayo, Santander, Sucre, Tolima, Valle del Cauca, Vichada (Fernández et al. 2019).

## Discussion

All the ants recorded here come from the Andean-Amazonian transition zone located in the Department of Caquetá. Soil sampling in this transition zone using both TSBF and Winkler sacs methods allowed us to collect seven species of ants previously unknown for Colombia and 14 species with no previous records for the Department of Caquetá. These ant species are distributed in five subfamilies and 16 genera, as follows: Myrmicinae (12 species), Ponerinae (5), Dolichoderinae (2), Formicinae (1) and Proceratiinae (1). For Caquetá, approximately 75% of the genera (82) and 26% of the species (322) known in Colombia are recorded. Our data combined with others (Castro et al. 2018, Castro et al. 2023) obtained within the AATZ suggest that this region presents a relatively unique ant fauna, that is, a high turnover of species (% similarity in parentheses) compared to Departments located in relatively close biogeographic regions, such as Amazonas (53%, Amazon Region), Meta (48%, Orinoquía Region) and Huila (21% Andean Region). The similarity of ants from Caquetá is also low compared to that of those Departments with a relatively known fauna, for example, Valle del Cauca (43%), Antioquia (35%) and Magdalena (30%). The pattern of replacement or dissimilarity between the ant fauna of Caquetá and other regions of Colombia suggests that AATZ could be playing a fundamental role in the establishment of Andean and Amazonian ant communities that take advantage of the diversity of microhabitats for the establishment of their colonies, promoting the co-existence of a high diversity of ant species (Longino and Colwell 1997), as well as some biogeographically remarkable taxa in this transition zone.

Soil and subsoil ants have been one of the least represented groups in edaphic diversity inventories (Wong and Guénard 2021), which has led to large gaps in information on the distribution of the species that are part of these communities. The application of the TSBF method to sample ants in AATZ made it possible to fill part of these gaps, since genera such as *Stegomyrmex* and *Oxypoecus* are recorded for the first time for the Colombian Amazon Region. *Stegomyrmexmanni* is the only species of the genus recorded in Colombia with populations showing a cis-Andean distribution; the record here from AATZ (trans-Andean distribution), however, suggests that the populations of this species have a wider distribution ranging from Andean forests (Serna 2002), dry forest (Camargo-Vanegas et al. 2024) to Ecuadorian Amazonian rainforest (Ryder et al. 2007). On the other hand, although the genus *Oxyepoecus* has been previously recorded for Colombia (Fernández et al. 2019), *Oxyepoecusephippiatus* is recorded for the first time in Colombia. The discovery of *O.ephippiatus* in the Colombian Amazon is expected considering its previous record in the Brazilian and Ecuadorian Amazon rainforest (Ryder et al. 2010b).

The application of the TSBF method to sample ants living on the ground not only allows us to complement the inventories and distributions of ant species, but this technique becomes important to increase the understanding of the biology of the species that can be collected on the monoliths. We provide information for five species of ants recorded for the first time living inside the soil. *Stegomyrmexmanni* is a common soil inhabitant and is collected infrequently using Winkler bags; however, this is the first record of the species showing hypogeic habits. The *Strumigenysprospiciens* queen collected 0 to 10 cm deep in the soil suggests a hypogeic lifestyle. For *Centromyrmexgigas*, there are few natural history details, but the hypogeic style recorded here could be a strategy to find termite mounds on which they feed, as suggested by Delabie et al. (2000). All specimens of the two *Wadeura* species recorded here were collected 20 cm deep within the soil. The few known records of *Wadeuraholmgrenita* come from variable habitats (Branstetter and Longino 2022), but our data suggest that it is an abundant species in the cloud forest of the Andean-Amazonian transition zone. On the other hand, *Wadeurapauli* appears to be a species restricted to the Amazon (Brazil, Colombia and Guyana), but with few records probably due to its hypogeic habits (Fernandes and Delabie 2019); like the type series of the species, the three specimens collected here also come from interior soil, contrasting with a single record from Guyana extracted from sifted leaf-litter (Fernandes and Delabie 2019).

Biodiversity research in Caquetá has been limited due to social problems arising from the armed conflict. This has created a gap in knowledge of its biodiversity, making it difficult to understand and conserve local ecosystems. However, the peace agreements in Colombia (2016), have opened the possibility of sampling in areas that were previously in armed conflict, this having a positive effect on myrmecological studies in the country (Guerrero et al. 2018, García et al. 2020). New species records for the country and the distribution expansion of other species offered here provide insight into the diversity of ants in little explored areas, adding information that complements the inventory of ant diversity in Colombia. On the latter, no updated information on ant species richness is available, other than that of García et al. (2020). Based on the records presented here and recent taxonomic work of genera for the Neotropical Region including specimens from Colombia (Ortiz-Sepuvelda et al. 2019, Camargo-Vanegas and Guerrero 2020, Ladino and Feitosa 2020, Mera-Rodríguez et al. 2020, Sozanski et al. 2020, Dias and Lattke 2021, Longino and Branstetter 2021, Ulysséa and Brandão 2021, Branstetter and Longino 2022, Fiorentino et al. 2022, Guerrero et al. 2022, Sanabria-Blandón and Achury 2022, Troya and Lattke 2022, Fernández et al. 2023, França et al. 2024, Guerrero et al. 2024, Arredondo and Guerrero 2025), we provide an update on ant richness in Colombia. In this sense, the current number of species in Colombia is 1280 and 110 genera, which corresponds to 41% of the species and nearly 80% of the genera from Neotropical Region (Fernández et al. 2021).

Finally, the records in this study can serve as a primary resource for the development of research that aims to understand the effect of natural barriers such as mountain ranges limiting the dispersal of species, as well as on the evolution of ants that inhabit areas within the ground.
